# Supplementary material for: FutureMS cohort profile: a Scottish multicentre inception cohort study of relapsing-remitting multiple sclerosis
Source: BMJ Open. 2022 Jun 29;12(6):e058506. doi: 10.1136/bmjopen-2021-058506 (PMC9244691; doi:10.1136/bmjopen-2021-058506)
Supplement: Supplementary data [file bmjopen-2021-058506supp001.pdf]

SUPPLEMENTARY FIGURES AND TABLES

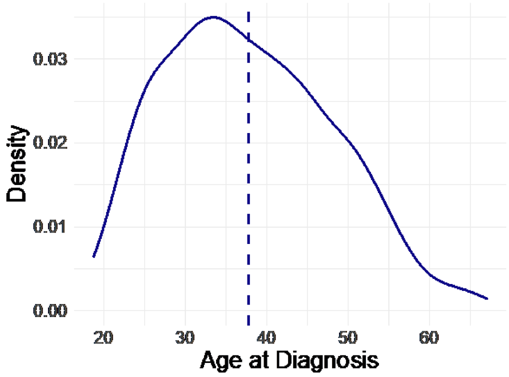

Figure S1. Age at diagnosis profile of the FutureMS participants. Demonstrating monomodal distribution and truncation at extremes of age (particularly <18) due to inclusion criteria. Dotted line is the mean age at diagnosis.

ASSESSMENT	Baseline	Year 1	Year 5
Informed Consent	**X**		**X**
Demographics	**X**		
Medical History	**X**	**X**	**X**
Social History	**X**	**X**	**X**
Family History	**X**	**X**	**X**
Migration History			**X**
Relapse/Progression History	**X**	**X**	**X**
Medication History	**X**	**X**	**X**
Infectious Mononucleosis History			**X**
Structured Neurological Examination	**X**	**X**	**X**
BMI, Height, Weight, BP	**X**	**X**	**X**
Measures of Physical Disability (EDSS, T25FTWT, 9HPT)	**X**	**X**	**X**
Cognitive tests (PASAT-3, SDMT)	**X**	**X**	**X**
PHQ-9 and GAD-7	**X**	**X**	**X**
MSIS-29	**X**	**X**	**X**
Fatigue Severity Score	**X**	**X**	**X**
Patient Derived Disease Severity Score	**X**	**X**	**X**
Visual Acuity	**X**	**X**	**X**
Standard MRI Protocol	**X**	**X**	**X**
Disease Modifying Therapy History	**N/A**	**X**	**X**
Lymphocyte subsets stored (CD3+CD4+, CD3+CD8+, CD19+, CD14+)	**X**		
Serum stored	**X**		**X**
Saliva stored			**X**
DNA Genotyping	**X**		
Advanced MRI Imaging (SS3)	**X**	**X**	**X**
Optical Coherence Tomography (SS4)	**X**	**X**	**X**

Table S1. Summary of measures at each study visit. SS3 = Substudy 3. SS4 = Substudy 4.

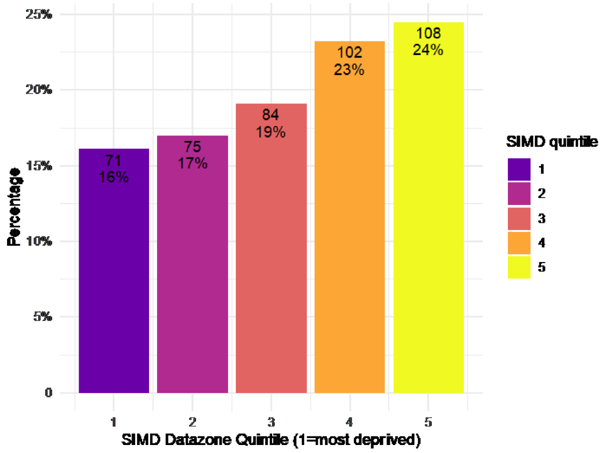

Figure S2. Scottish Index of Multiple Deprivation (SIMD) quintile of study participants.

	Parent/Grandparent	% of Parent				% of Grandparent			
		Mean	SD	LQ10	UQ10	Mean	SD	LQ10	UQ10
Single cells	Events	56.05	15.98	35.60	75.00				
Viable (7AAD -ve)	Single cells	91.77	10.11	97.80	84.93.				
CD14+ Monocyes	CD3-/viable cells	22.03	11.67	34.30	10.40				
CD19+ B cells	CD3-/viable cells	8.11	3.69	4.08	12.9				
CD4+ T cells	CD3+/viable cells	69.44	11.67	81.47	57.00	38.97	10.48	51.27	26.56
CD8+ T cells	CD3+/viable cells	20.27	8.31	30.37	11.22	20.27	8.31	30.37	11.22

Table S2. Proportion of PBMCs expressing markers for monocytes (CD14+), B/plasma cells (CD19+), helper (CD3+CD4+) and cytotoxic (CD3+CD8+) t cells in the peripheral blood mononuclear cells donated by patients to the study.

	**With PBMCs**	**FACs sorted**	**Total**
Edinburgh (01)	184	184	185
Glasgow (02)	163	137	165
Dundee (03)	46	46	46
Aberdeen (04)	35	32	35
Inverness (05)	8	6	8
Controls	100	78	103
**Totals (patients only)**	**436**	**405**	**439**
**Totals**	**536**	**483**	**542**

Table S3: Summary of numbers of FutureMS study subjects and controls with peripheral blood mononuclear cells available for analysis.

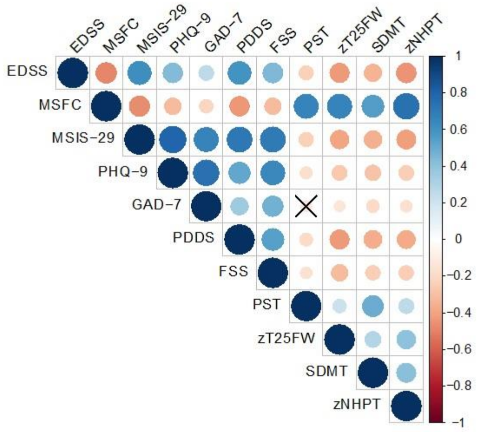

Figure S3. Correllelogram of clinical measures at baseline visit. zT25FW is the z-score of the timed 25-foot walk test. zNHPT is the z-score of the 9-hole peg test. Other acronyms as defined in the main text.

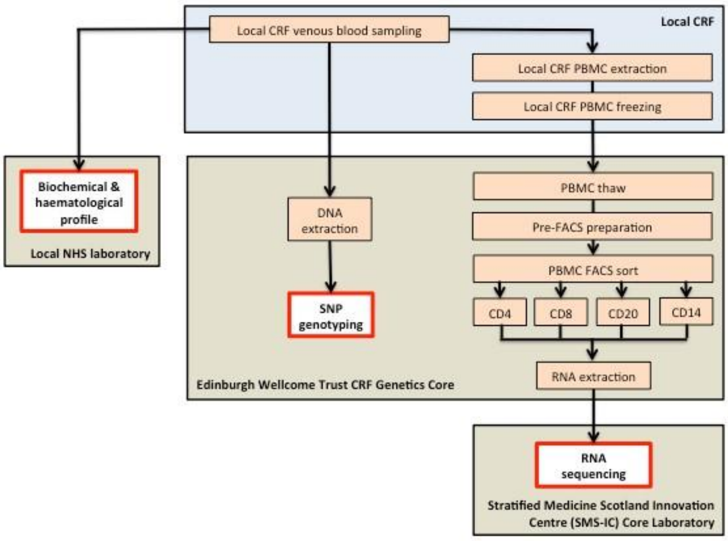

Figure S4: Schematic explaining laboratory pathway for tissue collection for biomarker processing.

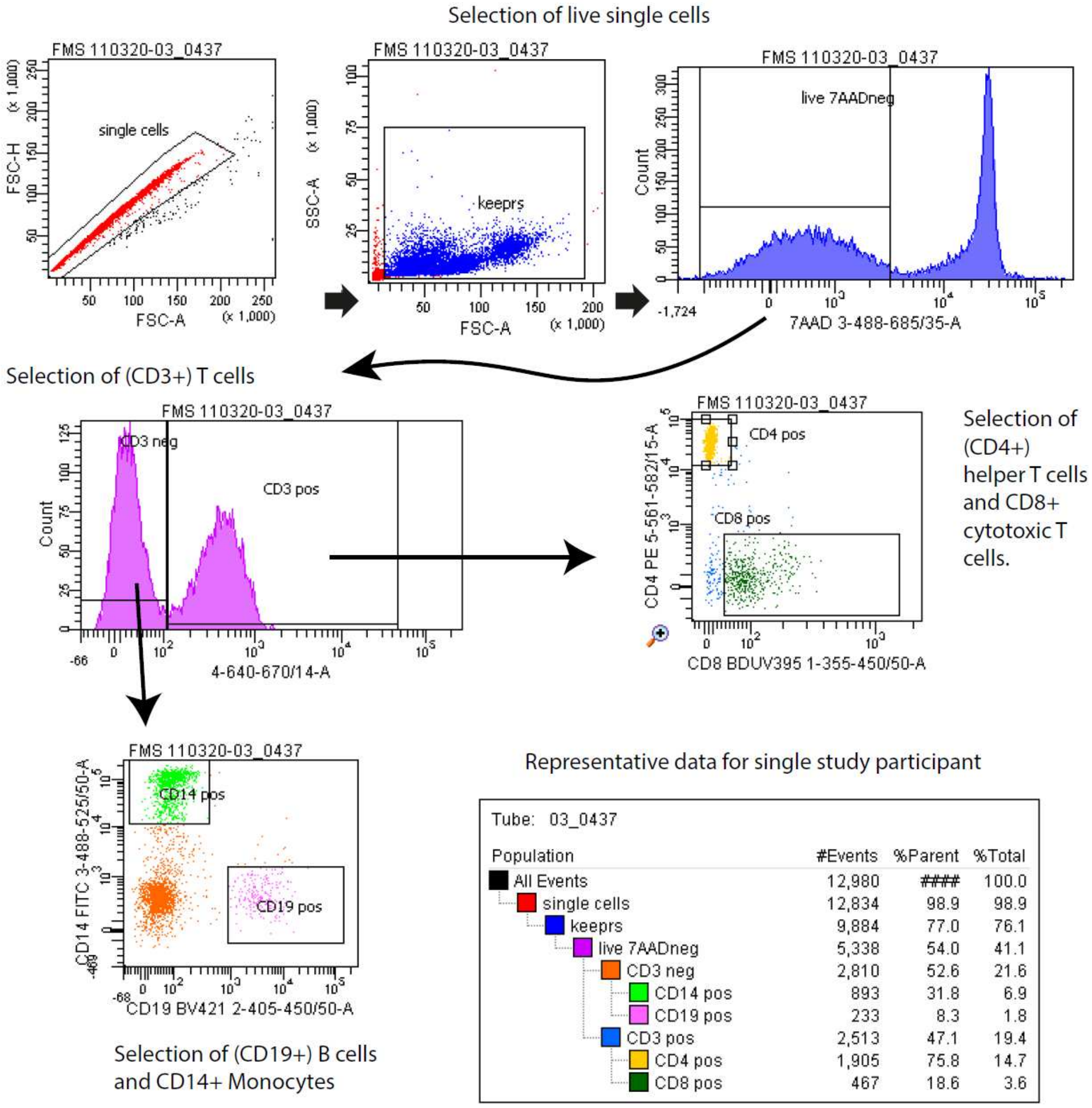

Population	#Events	%Parent	%Total
All Events	12,980	####	100.0
single cells	12,834	98.9	98.9
keeprs	9,884	77.0	76.1
live 7AADneg	5,338	54.0	41.1
CD3 neg	2,810	52.6	21.6
CD14 pos	893	31.8	6.9
CD19 pos	233	8.3	1.8
CD3 pos	2,513	47.1	19.4
CD4 pos	1,905	75.8	14.7
CD8 pos	467	18.6	3.6

Figure S5. Example of the fluorescence-activated cell sorting undertaken for a representative member of the FMS cohort. CD3 – T cell marker. CD4+ helper T cells, CD8+ cytotoxic T cells. CD19+ B cell marker and CD14+ monocyte marker. 7AAD – 7-Aminoactinomycin D uptake associated with loss of cell viability.

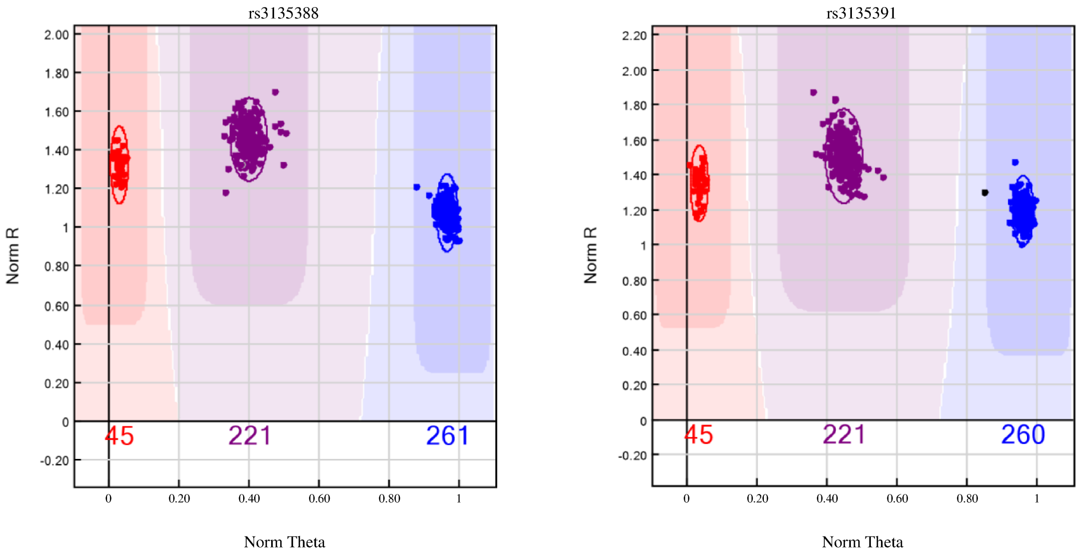

*Figure S6. Demonstration of two SNPs in linkage disequilibrium from the FutureMS cohort demonstrating clear separation of clusters resulting in high confidence of genotype calls. These two SNPs mark HLA-DRB1*15:01, which contributes the largest single gene effect for multiple sclerosis.*

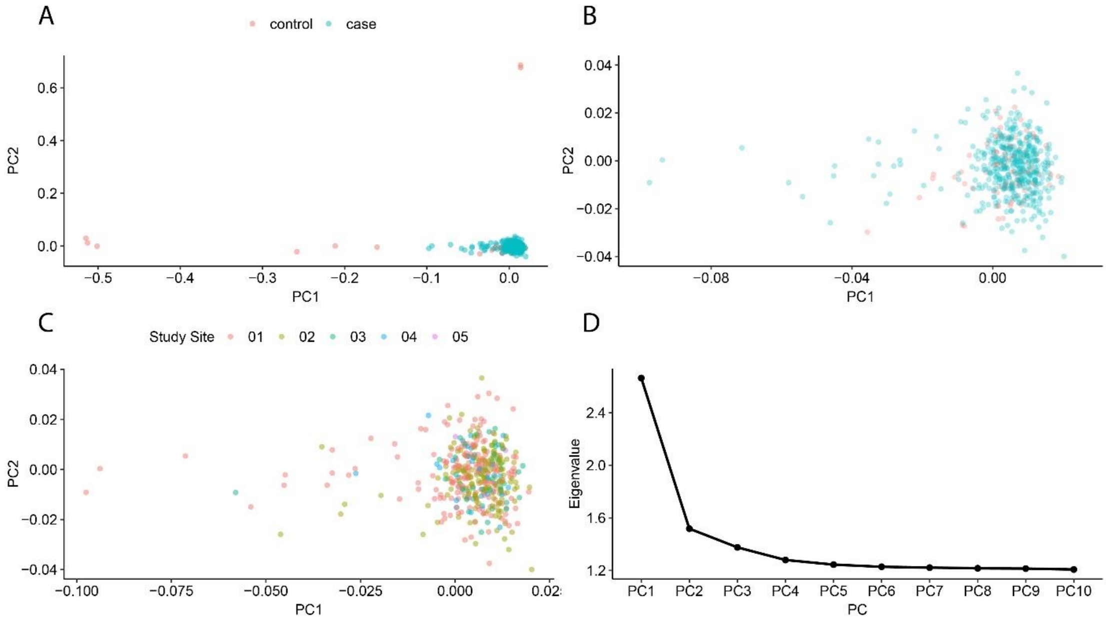

Figure S7. Genetic stratification analysis of the FutureMS cohort and controls. A – plot of first two principal components, comparing cases and controls. B – enlarged region of A with most cases and controls tightly clustered. Note difference of scale in PCs in A&B and A&C. C – Cases demonstrate little evidence of genetic stratification within the cohort based on the study site they were recruited from (scaled as in B). D – Scree plot of Eigenvalues of first ten principal components.
