# Supplementary material for: FutureMS cohort profile: a Scottish multicentre inception cohort study of relapsing-remitting multiple sclerosis
Source: BMJ Open. 2022 Jun 29;12(6):e058506. doi: 10.1136/bmjopen-2021-058506 (PMC9244691; doi:10.1136/bmjopen-2021-058506)
Supplement: Author's manuscript [file bmjopen-2021-058506.draft_revisions.pdf]

BMJ Open is committed to open peer review. As part of this commitment we make the peer review history of every article we publish publicly available.

When an article is published we post the peer reviewers' comments and the authors' responses online. We also post the versions of the paper that were used during peer review. These are the versions that the peer review comments apply to.

The versions of the paper that follow are the versions that were submitted during the peer review process. They are not the versions of record or the final published versions. They should not be cited or distributed as the published version of this manuscript.

BMJ Open is an open access journal and the full, final, typeset and author-corrected version of record of the manuscript is available on our site with no access controls, subscription charges or pay-per-view fees (http://bmjopen.bmj.com).

If you have any questions on BMJ Open's open peer review process please email info.bmjopen@bmj.com

BMJ Open

FutureMS Cohort Profile: A Scottish Multi-Centre Inception Cohort Study of Relapsing-Remitting Multiple Sclerosis.

Journal:	*BMJ Open*
Manuscript ID	bmjopen-2021-058506
Article Type:	Cohort profile
Date Submitted by the Author:	27-Oct-2021

Complete List of Authors:	Kearns, Patrick; The University of Edinburgh Centre for Clinical Brain Sciences, Anne Rowling Regenerative Neurology Clinic; The University of Edinburgh MRC Human Genetics Unit, Chromatin Lab, Genome Regulation Section<br>Martin, Sarah; The University of Edinburgh Centre for Clinical Brain Sciences; NHS Greater Glasgow and Clyde, Institute of Clinical Neurosciences<br>Chang, Jessie; The University of Edinburgh Centre for Clinical Brain Sciences, Anne Rowling Regenerative Neurology Clinic<br>Meijboom, Rozanna; The University of Edinburgh Centre for Clinical Brain Sciences, Anne Rowling Regenerative Neurology Clinic<br>York, Elizabeth; The University of Edinburgh Centre for Clinical Brain Sciences, Anne Rowling Regenerative Neurology Clinic<br>Chen, Yingdi; The University of Edinburgh Centre for Clinical Brain Sciences, Anne Rowling Regenerative Neurology Clinic<br>Weaver, Christine; The University of Edinburgh Centre for Clinical Brain Sciences, Anne Rowling Regenerative Neurology Clinic<br>Stenson, Amy; The University of Edinburgh Centre for Clinical Brain Sciences, Anne Rowling Regenerative Neurology Clinic<br>Hafezi, Katarzyna; Wellcome Trust Clinical Research Facility Edinburgh<br>Thomson, Stacey; The University of Edinburgh MRC Institute of Genetics and Molecular Medicine<br>Freyer, Elizabeth; The University of Edinburgh MRC Institute of Genetics and Molecular Medicine<br>Murphy, Lee; Wellcome Trust Clinical Research Facility Edinburgh<br>Harroud, Adil; Weill Institute of Clinical Neuroscience, Department of Neurology<br>Foley, Peter; The University of Edinburgh Centre for Clinical Brain Sciences, Anne Rowling Regenerative Neurology Clinic<br>Hunt, David; The University of Edinburgh Centre for Clinical Brain Sciences, Anne Rowling Regenerative Neurology Clinic<br>McLeod, Margaret; Aberdeen Royal Infirmary, Department of Neurology<br>O'Riordan, Jonathon; University of Dundee Division of Neuroscience, Tayside Centre for Clinical Neurosciences<br>Carod-Artal, F.J.; Raigmore Hospital, Department of Neurology<br>MacDougall, Niall; Wishaw General Hospital, Department of Neurology<br>Baranzini, Sergio; Weill Institute of Clinical Neuroscience, Department of Neurology<br>Waldman, Adam; The University of Edinburgh Centre for Clinical Brain Sciences<br>Connick, Peter; University of Edinburgh, Centre for Clinical Brain

	Sciences<br>Chandran, Siddharthan ; The University of Edinburgh Centre for Clinical Brain Sciences, Anne Rowling Regenerative Neurology Clinic

Keywords:	Neurology < INTERNAL MEDICINE, EPIDEMIOLOGY, Neurogenetics < NEUROLOGY, Multiple sclerosis < NEUROLOGY, Neuroradiology < NEUROLOGY, INTERNAL MEDICINE

SCHOLARONE™
Manuscripts

I, the Submitting Author has the right to grant and does grant on behalf of all authors of the Work (as defined in the below author licence), an exclusive licence and/or a non-exclusive licence for contributions from authors who are: i) UK Crown employees; ii) where BMJ has agreed a CC-BY licence shall apply, and/or iii) in accordance with the terms applicable for US Federal Government officers or employees acting as part of their official duties; on a worldwide, perpetual, irrevocable, royalty-free basis to BMJ Publishing Group Ltd ("BMJ") its licensees and where the relevant Journal is co-owned by BMJ to the co-owners of the Journal, to publish the Work in this journal and any other BMJ products and to exploit all rights, as set out in our licence.

The Submitting Author accepts and understands that any supply made under these terms is made by BMJ to the Submitting Author unless you are acting as an employee on behalf of your employer or a postgraduate student of an affiliated institution which is paying any applicable article publishing charge ("APC") for Open Access articles. Where the Submitting Author wishes to make the Work available on an Open Access basis (and intends to pay the relevant APC), the terms of reuse of such Open Access shall be governed by a Creative Commons licence – details of these licences and which Creative Commons licence will apply to this Work are set out in our licence referred to above.

Other than as permitted in any relevant BMJ Author's Self Archiving Policies, I confirm this Work has not been accepted for publication elsewhere, is not being considered for publication elsewhere and does not duplicate material already published. I confirm all authors consent to publication of this Work and authorise the granting of this licence.

For peer review only

FutureMS Cohort Profile: A Scottish Multi-Centre Inception Cohort Study of Relapsing-Remitting Multiple Sclerosis.

AUTHORS:

P.K.A. Kearns*[1,2,3,4], S.J. Martin[2,5], J. Chang[1,2,3], R. Meijboom[2], E.N. York[2], Y. Chen[1,2], C. Weaver[1,2], A. Stenson[1,2], K. Hafezi[6], S. Thomson[3], E. Freyer[3], L. Murphy[6], A. Harroud[7], P.Foley[1,2], D. Hunt[1,2], M. McLeod[7], J. O'Riordan[6], F.J. Carod-Artal[7], N.J.J. MacDougall[8], S.E. Baranzini[7], A.D. Waldman[2], P. Connick[1,2,4] S. Chandran*[1,2,4]

Affiliations:

1. Anne Rowling Regenerative Neurology Clinic, University of Edinburgh, UK
2. Centre for Clinical Brain Sciences, University of Edinburgh, UK
3. Institute of Genetics and Cancer, University of Edinburgh, UK
4. Department of Clinical Neurosciences, Edinburgh Royal Infirmary, NHS Lothian, UK
5. Department of Neurology, NHS Greater Glasgow and Clyde, UK
6. Wellcome Trust Clinical Research Facility, University of Edinburgh, UK
7. Department of Neurology, Aberdeen Royal Infirmary, NHS Grampian, UK
8. Tayside Centre for Clinical Neurosciences, University of Dundee, UK
9. Department of Neurology, Raigmore hospital, NHS Highlands, UK
10. Department of Neurology, Wishaw General Hospital, NHS Lanarkshire, UK
11. Weill Institute for Clinical Neuroscience, Department of Neurology, University of California San Francisco, USA.

Corresponding authors*: Dr Patrick Kearns (P.Kearns@ed.ac.uk) & Prof S.Chandran (Siddharthan.chandran@ed.ac.uk)

ABSTRACT:

Purpose: Multiple sclerosis (MS) is an immune-mediated, neuroinflammatory disease of the central nervous system and in industrialised countries is the most common cause of progressive neurological disability in working age persons. However, there is significant inter-individual heterogeneity in disease activity and response to treatment. Currently, the ability to predict at diagnosis who will have a benign, intermediate, or aggressive disease course is very limited. There is therefore a need for integrated predictive tools to inform individualised treatment decision-making.

Participants: FutureMS is a nationally representative, prospective observation cohort study comprising of 440 participants with a new diagnosis of relapsing remitting MS living in Scotland between May 2016 and March 2019. Established with the aim of addressing this need for individualised predictive tools, the cohort is designed to combine detailed clinical phenotyping with imaging, genetic and biomarker metrics of disease activity and progression.

Findings: Recruitment, baseline assessment and follow up at year one is complete and longer term follow up is planned, beginning at five years after first visit. The study aims to explore the pathobiology and determinants of disease heterogeneity in MS. Here we describe the cohort design and present a profile of the participants at baseline and one year of follow up.

Main strengths and limitations:

Strengths:

- To our knowledge, this is the largest inception cohort (n=440) of individuals prospectively recruited with Relapse-Remitting MS before starting on immunotherapies and is resourced to continue follow up to at least 10 years with deep genetic, clinical and radiological phenotyping.
- Efforts have been made to ensure participation of any eligible patient diagnosed with MS across Scotland during this time to ensure recruitment and retention of a representative cohort and patients and public stakeholders have been involved in study design and adaptation since the outset.
- State-of-the-art radiological and genetic/transcriptomic profiling has been conducted on all participants and serum biobanked for biomarker studies which offers a powerful resource for hypothesis testing and the discovery of the biological basis of disease.
- Deep clinical phenotyping measures objective and patient-reported outcomes and important socio-economic and psychosocial outcomes allowing for a holistic assessment of participant health and well-being.

Limitations

- Follow-up time at this point is short (each participant has been followed for only one year).
- Observational (cohort) design carries limitations.
- Follow up for a subset of participants was interrupted by COVID-19 restrictions to normal research practice (although these participants will still be invited to participate in subsequent study waves).
- Date presented here are a descriptive cohort profile. As only two time points are available over a short period, longer term trends in disease trajectory will not yet be apparent.

For peer review only

INTRODUCTION, BACKGROUND AND RATIONALE

Multiple sclerosis (MS) is the leading cause of progressive neurological disability in young and working age persons in middle- and high-income countries, and is a paradigm of neuroinflammation, autoimmunity and neurodegeneration[1]. 85-90% of incident cases have a relapsing-remitting disease course (RRMS) at onset, characterised by periods of clinical symptoms emerging and resolving. After a median of approximately 20-years, the disease moves into a phase of progressively accumulating irreversible disability called secondary progressive MS. The other 10-15% of incident cases experience this progressive phase from onset (primary progressive MS). In the RRMS group, both inflammation and neuronal injury are present throughout the disease course, with multifocal inflammatory demyelination dominant in the RR phase and neurodegeneration the key pathological substrate of the progressive phase[1]. Whilst the disease remains incurable, and untreated typically results in accumulation of substantial disability and a 5-15 year reduction in life expectancy, the emergence of effective disease modifying therapies (DMTs) for the early phase of disease has transformed the outlook for people living with RRMS in recent years[2,3].

rMS has a markedly heterogenous natural history; cases of aggressive progression and relatively indolent disease occur on a spectrum even in untreated individuals. The ability to predict future disease activity for an individual is very limited and still reactive in practice, extrapolating from radiological or clinical evidence of past disease activity. Informed treatment and lifestyle decision-making by people newly diagnosed with MS requires predictive tools available at or close to the point of diagnosis. Increasing DMT options, with attendant side effect profiles, highlights the urgent need for accurate and personalised prognostic tools.

DMTs do not appear to treat all biological aspects of MS pathology equally. They are more effective at preventing neuroinflammation than at halting neurodegeneration. Neurodegeneration is also hard to measure over short time periods, complicating early prediction. It may be essential for personalised predictive tools to be capable of discriminating between different biological contributions to disability progression. Early predictors and determinants of neuroinflammation may differ from those predicting the rate of neurodegenerative disease activity. In order to unpick this complexity, long-term longitudinal follow up of adequately-powered and representative clinical cohorts, starting as early as possible in disease course, which are resourced to “deeply phenotype” participants, could be a vital contribution towards achieving this personalised decision making.

One such cohort, FutureMS, is now fully recruited in Scotland and the first follow-up wave at one year has been completed. FutureMS is a large (n = 440) prospective inception cohort study recruiting newly diagnosed persons with relapsing-remitting MS (RRMS) living in Scotland at the time of their diagnosis. With a high incidence of MS, a stable population of 5.4. million, low rates of migration, and a national single-payer universal healthcare system free at the point of use, Scotland offers an ideal setting for a long-term longitudinal study of pwMS[4].

The FutureMS study hypothesis is that inter-individual variability in disease activity in RRMS is determined and will be predictable by a combination of clinical, laboratory, imaging and genetic parameters. The primary aim is to develop predictive tools for focal neuroinflammatory disease activity based on clinical, laboratory, magnetic resonanceimaging (MRI) and genomic assessment in patients with RRMS. Secondary outcomes include the development of predictive tools for a) neurodegenerative disease activity, b) clinical measure of disease activity, and c) clinical measures of quality of life. The study is structured in waves at baseline (within six months of diagnosis), at month 12 (baseline + 12 months), and future follow-up is planned at five years (baseline +5 years), and subsequently thereafter. Given that MS is a chronic long-term condition, it is expected that further insights will emerge from study of the cohort over time. Future MS aims to reduce uncertainty in disease trajectory and to allow for more tailored and personalized care for pwMS. This paper is intended to provide an overview of the study design and introduce a profile of the study participants.

For peer review only

PARTICIPANTS: Nationally representative cohort study

Between May 2016 and March 2019, 440 adult patients (age ≥ 18 years) were recruited as a nationally representative incidence sample within six months of their diagnosis (median time since diagnosis at first study visit: 60 days, IQR: 61 days). To ensure national representativeness, the study was designed to support inclusion of any person newly diagnosed with RRMS wherever they may live in Scotland, aiming to establish both geographically and socioeconomically representative coverage of the Scottish mainland and islands.

[Figure 1 approx. here]

Participants were recruited from the five main tertiary Scottish clinical neurology centres: 185 from Edinburgh (42.0%), 164 from Glasgow (37.3%), 46 from Dundee (10.5%), 35 from Aberdeen (8.0%), and 8 from Inverness (1.8%). This roughly reflects the geographic distribution of the population of Scotland, and the geographic incidence burden of MS[4] (see. Fig 1.). Analysis of the Scottish Multiple Sclerosis Register (SMSR)), a national incidence register with mandatory-reporting for MS specialist nurses at the time of referral for newly diagnosed persons, reveals that 45% of all persons diagnosed with RRMS in Scotland over this period were recruited to FutureMS. Comparison with the demographic characteristics from the SMSR suggests a broadly representative sample was recruited (table 1). FutureMS participants were slightly younger on average, less represented at the extremes of age distribution (Fig S1.) and more likely to be female. As has been observed in the SMSR data, there was a significant excess of persons living in affluent Scottish Index of Multiple Deprivation[5] (SIMD) quintiles relative to deprived quintiles (SIMD: $X^2 = 14.06$, 4d.f., $p<0.01$; and FutureMS: $X^2 = 12.2$, 4 d.f., $p <0.05$) (Fig S2). This has been recognized in multiple epidemiological studies in Scotland[6,7] and is not apparently explained -- in fact paradoxical -- given the burden of established MS environmental risk factors (e.g. Vitamin D deficiency, obesity in adolescence and smoking), which are strongly all associated with deprivation in Scotland[8–11].

Amongst FutureMS participants who listed their ethnicity, 426/440 (96.8%) recorded their primary ethnicity as White Scottish/British. For those with recorded ethnicity in the first eight years of the SMSR (2010-2018), the proportion recorded as Scottish, British or Irish was similar 862/919 (93.8%).

Patient and Public Involvement

Between May 2016 and March 2019, 440 adult patients (age ≥ 18 years) were recruited as a nationally representative incidence sample within six months of their diagnosis (median time since diagnosis at first study visit: 60 days, IQR: 61 days). To ensure national representativeness, the study was designed to support inclusion of any person newly diagnosed with RRMS wherever they may live in Scotland, aiming to establish both geographically and socioeconomically representative coverage of the Scottish mainland and islands.

[Table 1 approximately here]

Diagnostic inclusion criteria

Diagnosis in all cases was confirmed by the treating consultant neurologist as fulfilling the most recent McDonald Criteria. Participants must not have commenced on DMT prior to baseline assessment, had

capacity to give informed consent and have had no contraindication to MR brain imaging at the time of their baseline visit.

Only patients with a diagnosis of RRMS were eligible for inclusion in FutureMS. We excluded those with progression at onset. Diagnosis of progressive forms of MS is typically delayed relative to RRMS, requiring a period of observation of sustained progression, and recent studies have suggested that the distinction between relapsing and progressive forms of MS are not clearly demarcated clinical entities. Rather that they reflect different stages of the same disease[12]., inclusion was limited to persons with RRMS.

CONTROLS FOR LABORATORY AND BIOMARKER STUDIES

103 healthy volunteers were recruited from the Lothian area to donate blood and DNA for the biomarker and genetic analyses. These persons were age- and sex- frequency matched to the study population. All were recruited in the Anne Rowling Regenerative Neurology Clinic in Edinburgh, and so are mainly drawn from the surrounding Edinburgh and Lothian areas.

STUDY VISITS

Study visits were in addition to, and not in place of, standard neurological care. Consequently, the timing of the first visit is likely to be associated with temporal fluctuations in the participants' disease activity, as diagnosis is more likely following clinical relapse. Subsequent visits should be independent of this potential bias being at fixed intervals after first visit and not triggered by clinical disease activity. We consider it a strength that the study design reduces clinically-triggered follow up biases.

All clinical management decisions were reserved to the treating team. Participation in FutureMS is not a barrier to participating in any other research study including interventional trials, and we anticipate that a substantial number of participants will choose to engage with other research studies. **[Figure 2 approximately here]**

Demographic and clinical variables collected at baseline included date of birth, sex, ethnicity, occupation, co-morbidities, medication history (including 'over the counter' and supplements), and family history. Data pertaining to the diagnosis of RRMS (description of initial symptoms, number of clinical relapses, hospitalisations, and steroid use) were recorded at baseline visit, and all data were also updated at the twelve-month review (Table S1).

At the time of writing, 392 of 440 participants (89.1%) had completed the return visit at year one. Six patients had withdrawn consent (1.4%), 23 (5.2%) were lost to follow up but will be invited to participant in subsequent visits. 19 (4.3%) participants had their return visit at one year prevented by the COVID-19 pandemic (visits due in March or April 2020) but these participants will be invited for future follow up.

Sub-studies

Alongside the main study, four additional 'opt in' sub-studies allowed deeper phenotyping of participants. Sub-studies included consenting participants to approach them with opportunities for future research/cross-linkage with other studies (sub-study 1); biobanking an additional large volume blood sample at baseline visit (sub-study 2); retinal imaging with optical coherence tomography at baseline and twelve-month follow-up visit (sub-study 3); and additional advanced MRI imaging sequences (sub-study 4, baseline n = 78, follow up n = 74, complete pairs n = 67).

For peer review only

CLINICAL OBSERVATIONS

Patient-reported and assessor-measured clinical observations were collected at each study visit. Source data from clinical assessments at local sites was captured using a web-based electronic case report form (eCRF). Both participants and study staff entered data directly. Clinical data were entered by participants via questionnaires. There was a high level of engagement with these questionnaires and assessments. Data completeness was >99% across all clinical measured and reported variables at both the baseline and year 1 visits.

Questionnaires included the multiple sclerosis impact scale (MSIS-29)[13], NICE domain activity & impairment, CDC Health Related Quality of Life (HRQOL-4)[14], Patient Determined Disease Steps (PDDS)[15], Fatigue Severity Scale (FSS)[16], Generalized Anxiety Disorder Assessment (GAD-7)[17], depression assessment scoring (PHQ-9)[18], Baecke Habitual Physical activity, cognitive, leisure, social and lifestyle questionnaires. Clinical measures included the Expanded Disability Severity Score (EDSS)[19], components of the Multiple Sclerosis Functional Composite score (MSFC)[20], mean arterial blood pressure (BP) and Body Mass Index (BMI).

On some measures participants improve on average over the first year. This is not unexpected as diagnosis often coincides with a clinical event/relapse and we expect some regression toward the mean over the course of the first year which may be amplified by effective treatment for some participants. Many of these clinical measures and questionnaires capture overlapping phenomena and are correlated. This provides both opportunities and challenges for asking causal questions of longitudinal repeated measures (see Fig S4).

[Table 2 approximately here]

RESULTS

Lifestyle and Social Factors

[Figure 3 approximately here]

Lifestyle and social factors are known to influence MS disease course, and whilst some of these factors have been identified, there is much non-heritable variability that remains unexplained. Amongst the strongest known environmental factor is smoking[21]. There is strong evidence that smoking both modifies the risk of MS incidence and affects the rate of disability progression. Smoking has also been demonstrated to interact statistically with disease risk loci[22]. In our cohort, 14.7% of participants were current smokers at the baseline visit and more than half (50.7%) declared themselves to be "ever smokers". By year one, there has only been a modest reduction in participants smoking (13%). Figure 3A demonstrates that even at baseline, the distribution of disability differs by smoking status.

As MS is associated with significant disability in working-age persons, it has the potential to impact on employment and the quality of working life. Figure 3B demonstrates that at baseline the distribution of depression scores (PHQ-9) for persons who are employed and unemployed is quite different. The bimodal distribution of those who are unemployed, suggests that a significant proportion of pwMS who are unemployed at baseline are at risk of depression as assessed by this score.

Physical disability

For peer review only - http://bmjopen.bmj.com/site/about/guidelines.xhtml

[Figure 4 approximately here]

The distribution of measures of physical disability follow similar patterns across the two waves of the study cohort and there is little difference between these over this early period. This demonstrates the relatively insensitive nature of these measures early in disease course and over shorter study periods, at least at population level. However, FutureMS is sufficiently powered to allow meaningful comparison of sub-cohorts and of outlier individuals whose measured scores have worsened or improved in the first year. Figure 5 demonstrates one such analysis: the group who have worsened over the first year appear older and very few have low fatigue severity scores at baseline. Further analyses of these patterns may define groups that explain some of the heterogeneity in disease course.

[Figure 5 approximately here]

Fatigue, cognition and mood

[Figure 6 approximately here]

Fatigue has been described as the most disabling MS symptom by as many as 60% of patients in some studies[23]. The fatigue severity scale is therefore an important component of the study assessment of MS disease impact. The biological basis of fatigue is poorly understood. The distribution of participants suffering fatigue changes between the baseline and month 12 in our cohort. At follow up, the group does not appear to be monomodal which may reflect underlying biological heterogeneity or discrete sub-populations. Previous work has attempted to stratify fatigue into central or peripheral fatigue, and it may be that fatigue is a composite symptom with multiple pathogenic mechanisms. Investigation of the natural history, burden and biology of fatigue will be a focus of study in the FutureMS cohort.

We observed a significant burden of depression in the study cohort, as measured by PHQ-9, highlighting the important contribution of mental health to MS burden early in the natural history of the MS. Median depression scores improved statistically significantly over the course of the first year from 7 to 4 ($p<10^{-12}$, two-tailed Wilcoxon signed-rank test).

Cognitive impairment measured by SDMT and PASAT-3 tests revealed marked heterogeneity in the burden of impairment and heterogeneity in the trajectory of these measures between study waves. Whilst the SDMT and PASAT-3 scores are significantly correlated (Spearman's rho 44.7 at baseline and 48.7 at follow up, both $p<10^{-15}$), we found some participants struggled with PASAT-3 with those who fail the trial run recorded as zero contributing to a distribution of scores that was far from normal. Cognitive scores at baseline and follow up correlated statistically significantly ($p<10^{-15}$) for both tests: SDMT (rho = 0.8) and PASAT-3 (rho = 0.76).

Adjusted measures of MS Severity

[Figure 7 approximately here]

The multiple sclerosis severity score (MSSS) and age-related multiple sclerosis severity scale (ARMSS) have been validated on large independent cohorts to attempt to standardize physical MS-related disability for disease duration. The MSSS does this by normalising the EDSS for patient-reported disease duration. However, factors that influence recall of disease duration and EDSS may confound this measure. Therefore, ARMSS normalises EDSS for patient age which is correlated with disease duration imperfectly but is not susceptible to recall biases. These measures, ARMSS and MSSS,

For peer review only

correlate strongly and statistically significantly (all comparisons $p<10^{-15}$) in both study waves: rho = 0.69 at baseline and rho = 0.71 at year one (figure 7). The age-adjusted measures show good overall agreement with the participant-reported measure (MSSS).

MR IMAGING

MR brain image protocols and processing have been described in detail elsewhere [Meijboom et al, pre-print.], but in brief, participants from all centres were invited to undergo a standard protocol of structural 3T MRI sequences, including T1-weighted, T2-weighted and fluid attenuated inversion recovery (FLAIR) images. The study was powered to detect changes in brain imaging outcomes - not necessarily changes in clinical measures - at year one, as MR brain imaging measures have higher sensitivity over short time frames compared to clinical measures (e.g. annual number of relapses). The primary endpoint was new and/or enlarging T2 hyperintense lesions, as qualitatively (visually) assessed by expert neuroradiologists using brain imaging software. The secondary endpoint was automated measurement of global brain volume change. In addition to the standard structural sequences, participants in Edinburgh were invited to undergo an advanced MR imaging protocol, comprising diffusion MRI (dMRI) and magnetisation transfer imaging (MTI). These measures allow for quantitative assessment of brain microstructure and therefore provide the opportunity to study brain myelin and axonal damage, which are prominent features of MS. dMRI and MTI metrics were used as exploratory endpoints of microstructural change in MS.

OPTICAL COHERENCE TOMOGRAPHY

Participants of FutureMS were offered the opportunity to enrol in a sub-study of retinal imaging and optical coherence tomography. Proof of concept has been established in MS for the utility of retinal imaging with optical coherence tomography (OCT) to measure thinning of the retinal nerve fibre layer (RNFL), inner nuclear layer (INL), and the ganglion cell and inner plexiform layer (GCIP), all of which have been shown to correlate with clinical activity and disability[24].

LABORATORY INVESTIGATIONS

Blood sampling was performed at the baseline visit for routine laboratory testing, genetic testing, cell subsets, and biobanking for future studies. 'Routine' analysis included eGFR, HbA1c, CRP, vitamin D, albumin, cholesterol, high-density lipoprotein (HDL), low-density lipoprotein (LDL), very-low density lipoprotein (VLDL), haemoglobin concentration (Hb), white cell count (WCC), and platelet count. All clinical and laboratory (blood test) assessments were performed in a standard sequence by the assessing neurologist or clinical research nurse. All samples were transported to laboratories for analysis immediately after venepuncture. Routine laboratory predictors were analysed in local NHS labs using their standard local protocols (Fig S3.).

Blood was collected at baseline for DNA extraction and PBMC isolation. DNA was extracted from 9ml EDTA whole blood using Nucleon BACC3 kit. DNA samples were re-suspended in 1ml TE buffer pH 7.5 (10mM Tris-Cl pH 7.5, 1mM EDTA pH 8.0). Peripheral blood mononuclear cells (PBMC) were isolated from Lithium Heparin blood at each hub and samples shipped to the Edinburgh CRF Genetics Core Laboratory for storage.

For fluorescence activated cell sorting (FACS), samples and sorted PBMC populations were kept on ice at all times. Prior to sorting on the BD FACSAriaII SORP cell sorter, the instrument was set-up using the internal Cytometer Set-Up and Tracking (CS&T) system, the drop delay was set to >99.9% with Accudrop beads to ensure sort quality. The Aria was set up with the 85um nozzle and 45psi pressure. Single stained controls were analysed with every run before a sort and compensation adjusted if necessary. The sample chamber and collection tube holder were cooled to 4°C. Collection tubes were

For peer review only

pre-coated with 500uL cold medium. According to protocol, 5uL of 7-AAD were added to the cell sample 5 minutes before sorting, and the samples were filtered through 35um nylon mesh cell-strainers to avoid the risk of clumping samples interfering with the sort. Gates were set on FSC-H and FSC-A to determine single cells, SSC-A and FSCA to exclude debris and 7-AAD negative population to exclude dead cells 7-AAD+. The populations sorted were CD3+ CD4+ T-cells, CD3+ CD8+ T-cells, CD14+ Monocytes and CD19+ B-cells. The cells were run with a flowrate of 6.000 – 8.000 events per second. The maximum number of cells sorted per population was set to 1.5 x 10^6 for the larger populations, and as many as possible for the smaller populations. Upon completion of the sort, a different sorted population from each sample was reanalysed on the instrument to evaluate the post-sort purity of the fractions across the samples. The number of cells sorted were recorded and sorted populations passed for RNA extraction.

The fluorescent channels used were: 7-AAD excitation laser 488nm, 685/35nm BP filter, CD3-APC excitation laser 640nm, 670/14nm BP filter, CD14-FITC excitation laser 488nm, 525/50nm BP filter, CD19-BV excitation laser 405nm, 450/50nm BP filter, CD4-PE excitation laser 561nm, 582/15nm BP filter and CD8-BUV excitation laser 355nm, 450/50nm BP filter. RNA was extracted from sorted cell fractions using Qiagen miRNeasy. Yield and RIN were measured by Qubit RNA HS and Agilent Fragment Analyser. 1ng of total RNA sample was fragmented and first-strand cDNA was generated using the SMARTer Stranded Total RNA-Seq Kit - Pico Input Mammalian kitIllumina-compatible adapters and indexes were added via 5 cycles of PCR. AMPure XP beads (Beckman Coulter) were used to purify the cDNA library followed by ribosomal RNA depletion using ZapR and R-Probes. Uncleaved fragments were enriched by 15 cycles of PCR before a final library purification using AMPure XP beads and sequencing on an Illumina NovaSeq.

Summary results for cell proportions suggested significant inter-individual variability in the proportion of viable cells that were B cells, monocytes, or T cells (supplementary materials) despite the lack of DMTs in this cohort. Future work is intended to explore whether these are meaningful parameters predictive of current or future neuroinflammatory activity.

Additional (fluid) biomarkers of neuroinflammation have been analysed at baseline and will be described in detail elsewhere. These include neurofilament light chains (NfL), GFAP, Tau, UCH-L1 measured using digital ELISA/Single Molecule Array (SIMOA). CSF biomarkers have also been analysed for a subset of study participants.

[Figure 8 approximately here]

SNP GENOTYPING

Although environmental factors (particularly EBV infection, smoking, obesity during adolescence) are known to make important contributions to MS risk, there is an important heritable component evidenced from correlation between relatives[25]. The strongest known contribution to this heritability is for the HLA region of chromosome 6[26]. Despite the remarkable allelic heterogeneity observed at this region, HLADRB1*15:01 (marked by rs3135388[27] and several SNPs in high linkage disequilibrium) is known to dominate the contribution to this risk. However, in addition, over 200 non-HLA loci are associated with disease risk[28]. Less is known about genetic contributions to the variance of disease course. Figure S5 demonstrates the high confidence in calling (discriminating between) SNP genotypes linked to HLADRB1*15:01 in FutureMS and table 3 demonstrates the expected finding of significant overrepresentation of the HLA DRB1*15:01 risk loci.

[Table 3 approximately here] [Figure 10 approximately here]

For peer review only - http://bmjopen.bmj.com/site/about/guidelines.xhtml

However, as shown in Figure 10, despite dominating the contribution to MS risk, the HLA-DRB1*15:01 genotype does not explain much, if any, of the baseline heterogeneity in the age at diagnosis, measured disability severity, or participant reported disease impact in the FutureMS cohort. This underscores that risk genes (HLA-DRB1*15:01) may not necessarily intersect with the gene set that influences disease course. 713,026 SNPs are available for genome-wide analyses in the FutureMS cohort from successful genotyping of 427/428 cases and 100/100 controls for whom PBMCs were available for DNA extraction (see supplemental table and detailed methods). Investigation of the genetic and gene-environment interactions that explain heterogeneity and personal disease trajectories is a focus of ongoing analyses.

GENOTYPING MATERIALS AND METHODS

Extracted DNA was normalised to 50ng/ul after quantification using Qubit. Samples were genotyped using Infinium HTS chemistry and Infinium Global Screening Array-24 kit. Arrays were scanned on an Illumina iScan system and genotypes were called using GenomeStudio v2.0.3. Genotype calls using GenCall (v6.3.0) with a cut-off specified at 0.15, were then manually reviewed within Genome Studio, using a rigorous multi-step appraisal of cluster fit based on cluster separation score, call frequency, heterozygous excess, heterozygous mean normalized intensity and theta, and minor allele frequency. This was in line with manufacturer published instructions. Further QC was performed using PLINK v1.07 and R v3.5.2.

Data Management

Participants were identified with a unique non-identifiable study number, which was used to label all paperwork, biological samples and imaging obtained throughout the duration of the study. Questionnaires and clinical data were entered in real time to a FutureMS electronic case report form via an online platform. Data were managed in accordance with the Data Protection Act (DPA 1998), NHS Scotland, and University of Edinburgh policies (supplemental figure).

Missing Data Handling

Most (395/440) participants in the study recorded entirely complete (100%) baseline records comprising 189 variables in the core clinical dataset. Similar completeness of data was observed for month 12: >99% across all clinical measured and reported variables at both baseline and month 12 follow up. Where missing, source data were carefully inspected for clinical measures/variables and the likely cause for missingness was appraised by a multidisciplinary study team (study nurses and neurologists). Where data were missing at random, multiple imputation with chained equations by predictive mean matching was used to impute baseline measures from across the cohort. Data missing not at random (e.g. due to disability) were left missing where appropriate (e.g. for smoking status) or substituted where appropriate (e.g. when missing timed 25ft walk test due to EDSS > 6 a low z-score was substituted to reflect this disability based on previous cohorts).

Data Retention

Data acquired in FutureMS may be of potential long-term scientific value. All data collected will therefore be retained for a minimum of 30 years after study completion. Collected data will also be retained after the withdrawal of participants for any reason including loss of capacity. No identifiable data will be shared with third parties, but proposals for collaborative ethically approved research projects utilising these data will be welcomed and proposals considered.

Statistical analyses

For peer review only

Mixed effects regression models, latent class/transition models, network-based analyses are planned for subsequent investigation of relationships between variables and will be explained in detail elsewhere.

Ethics

The study is conducted in conformity with the declaration of Helsinki, ICH guidelines for Good Clinical Practice (CPMP/ICH/135/95), and with ethical approval granted from the National Health Service (NHS) South East Scotland Research Ethics Committee (02) (Reference: 15/SS/0233) and approval granted from individual NHS Board Research and Development departments (IRAS Project ID: 169955). Control samples were collected under approval granted from the NHS East of Scotland Research Ethics committee (REC01) as part of the Scottish Regenerative Neurology Tissue Bank Project (Reference: 15/ES/0094).

DISCUSSION, STRENGTHS AND LIMITATIONS

We have designed and recruited a large cohort of persons with RRMS across Scotland. The prospective nature of FutureMS enables longitudinal assessment of clinical, imaging, genomic and fluid biomarkers in all participants prior to and during disease modifying treatments. As the number of available treatment options increases, so too must our understanding of the heterogeneity of disease course for persons living with RRMS. Substantial effort has been made to ensure that the study has recruited a geographically, socioeconomically, and clinically as representative a cohort as possible. Results presented here give us confidence that this has been achieved such that findings from this study may generalize to the clinic and real world.

Scotland has long been recognized as having a high incidence of multiple sclerosis, for reasons that remain unknown despite long-running speculation[7]. The Scottish northern isles for many decades have been recognized as particularly burdened[30]. Our early exploration of genetic results confirms expected findings of an excess of HLA-DRB1*15:01 (OR 3.90, 95% CI: 2.50 – 6.34) in the Scottish MS population. This provides a useful prevalence benchmark by which this (and other) genetic loci can be assessed and compared to other MS populations. The risk allele frequency (RAF) in the FutureMS cases of 0.34 is high by previously published standards[31–34], but not extremely so with numerous historical case control studies reporting higher frequencies of this gene[35]. The frequency in controls (0.12), is similarly high, but not excessively so. Taken together, the excess frequency in cases underscores the highly probable importance of this gene's contribution to MS risk in Scotland (as elsewhere), but leaves room for other genetic or environmental factors to explain why Scotland has a high rate of MS. Substantial further exploration is required and intended to address this issue.

Recent and historical studies have noted regional variation in the distribution of this burden of MS across Scotland[4,36–38], consistent with findings in many other countries where regional analyses have been performed[39,40]. A strength of this study is that in being geographically representative of the national population it may be well positioned to investigate genetic and environmental hypotheses for this spatial heterogeneity in disease burden.

The exploratory analyses presented here demonstrate that our cohort can be considered nationally representative. That said, we suggest caution generalizing any findings from this population to individuals who fall outside of he remit of our study. For example, those who experience such aggressive disease at onset that DMT is initiated emergently (as these people would not have been eligible for recruitment had they been commenced on DMTs emergently at the time of diagnosis), or to those diagnosed at extremes of the age distribution (particularly <18). Similarly, caution may be necessary if attempting to generalize to populations with more heterogenous recent ancestry and to those whose initial presentation is with progressive disease.

Multiple sclerosis is a clinically heterogenous disease, presenting with variable symptoms affecting different parts of the central nervous system, which may be interspersed by prolonged periods without overt disease. This heterogeneity makes the diagnosis challenging and often delayed. Variability between clinicians can compound this heterogeneity. Although we used six months as a proxy for

For peer review only

'newly diagnosed' this does not necessarily equate to 'early' disease from a pathophysiological view, and this is an important limitation of our study. This is shown by the time taken from first symptom to diagnosis ranging from a single day to 33.5 years in the FutureMS cohort. It is perhaps inevitable, therefore that biological markers taken at baseline research will often not be reflect true disease initiation. However, a strength of this study is that participants were enrolled as early as possible after diagnosis. Whilst date of disease diagnosis will not be equivalent to date of disease onset, it is a best practical compromise.

Our early exploration of the association with disability severity and demographic and lifestyle factors highlighted an obvious difference, observable at baseline, in measures of physical disability between current and non-smokers. Importantly, the proportion of current smokers barely changed over the first year of the study, despite the wealth of evidence of the risk of smoking worsening disease control. This brings into focus the need to counsel all persons newly-diagnosed with MS who smoke, as early as possible, and to provide information on the benefits of cessation and the MS-specific harm of smoking, including passive smoking (which we didn't capture). It is likely that for some pwMS, particularly those for whom smoking is compounded with genetic pre-disposition, the effect of stopping smoking may be very substantial.

Fortunately, depression as measured by PHQ-9 is one of the clinical measures that improves most in the first year following diagnosis. However, we noted a high burden of high PHQ-9 scores at baseline, and particularly in persons diagnosed with MS who are also unemployed. Numerous possible explanations for the relationship between MS-depression and employment are possible, and further work will be necessary and is intended to delineate the causal structure of this relationship in order to guide effective treatment. However, these findings underscore the importance of considering the burden of mental health conditions in MS. It is reassuring that these scores improve on average in the early phase of the condition, and this may be of reassurance to some patients, and may encourage mental health treatment where mood is not improving.

In conclusion, we anticipate that long-term follow up of the FutureMS cohort will lead to the development and implication of clinical tools for predicating future disability in patients with multiple sclerosis.

Statements

Contributorship

P.K.A.K. performed data cleaning (assisted by C.W.,) performed the analyses and wrote the first draft of the paper. P.K.A.K., S.J.M., J.C., R.M., E.N.Y., contributed figures and/or analyses to the paper. P.K.A.K., S.J.M., Y.C., C.W., A.S., P.C., S.C., conducted patient visits and collected primary data. K.H., S.T., E.F., L.M., analysed laboratory data and performed genetics experiments. R.M., E.N.Y., Y.C., A.D.W., analysed radiological/imaging data. P.K.A.K., S.J.M., R.M., E.N.Y., C.W., A.S., A.H., P.F., D.H., A.D.W., P.C., S.C., planned analyses and managed data collection. P.C., M.McL., J.O.R., F.J.C-A., N.J.J. MacD., S.C., were responsible and oversaw study sites. S.E.B., A.H., L.M., planned and conducted genetics and transcriptomics experiments. P.C., A.W., S.C., conceived idea for cohort, designed, secured funding, approvals and established the study. All authors contributed to revising drafts of the initial manuscript. All authors satisfy ICMJE 2018 criteria for authorship.

Conflicts

The authors declare no conflicts of interest.

For peer review only - http://bmjopen.bmj.com/site/about/guidelines.xhtml

Funding

FutureMS has been funded by Precision Medicine Scotland Innovation Centre (PMS_IC: R44346 & R44800), the Rowling Clinic, and Biogen Idec. Ltd. Insurance provided by the Co-Sponsors: NHS Lothian and the University of Edinburgh. Funders had no role in the design of the study, collection of the data, or analysis of the data.

Data sharing

Data (and/or samples) are available after approval of a research proposal via an established sub-committee. The study team welcomes enquiries for collaboration or external research proposals (with appropriate approvals) using the cohort data and/or samples.

REFERENCES

1. Compston, A. *et al. McAlpine's Multiple Sclerosis*. (Churchill Livingstone Elsevier, 2006). doi:10.1016/B978-0-443-07271-0.X5001-0.

2. Kappos, L. *et al.* Ocrelizumab in relapsing-remitting multiple sclerosis: a phase 2, randomised, placebo-controlled, multicentre trial. *Lancet* **378**, 1779–1787 (2011).

3. Hauser, S. L. *et al.* B-Cell Depletion with Rituximab in Relapsing–Remitting Multiple Sclerosis. *N. Engl. J. Med.* **358**, 676–688 (2008).

4. Kearns, P. K. A. *et al. Regional variation in the incidence rate and sex ratio of multiple sclerosis in Scotland 2010–2017: findings from the Scottish Multiple Sclerosis Register. Journal of Neurology* http://link.springer.com/10.1007/s00415-019-09413-x (2019) doi:10.1007/s00415019-09413-x.

5. Ralston, K., Dundas, R. & Leyland, A. H. A comparison of the Scottish Index of Multiple Deprivation (SIMD) 2004 with the 2009 + 1 SIMD: does choice of measure affect the interpretation of inequality in mortality? *Int. J. Health Geogr.* **13**, 27 (2014).

6. Murray, S., Bashir, K., Penrice, G. & Womersley, S. J. Epidemiology of multiple sclerosis in Glasgow. *Scott. Med. J.* **49**, 100–4 (2004).

7. Bramwell, B. The Relative Frequency of Disseminated Sclerosis in this country (Scotland and the North of England) and in America. *Rev. Neurol. Psychiatry R. Coll. Physicians Edinburgh* **1**, 12 (1903).

8. Ascherio, A. & Munger, K. L. Environmental risk factors for multiple sclerosis. Part II: Noninfectious factors. *Ann. Neurol.* **61**, 504–513 (2007).

9. Tod, E. *et al.* Obesity in Scotland: a persistent inequality. *Int. J. Equity Health* **16**, 135 (2017).

10. NHS Health Scotland & ISD Scotland. *An Atlas of Tobacco Smoking In Scotland: A REPORT PRESENTING ESTIMATED SMOKING PREVALENCE AND SMOKING-ATTRIBUTABLE DEATHS WITHIN SCOTLAND.* http://www.healthscotland.com/uploads/documents/7882AnAtlasOfTobaccoSmokingInScotland2.pdf (2007).

11. Purdon, G., Comrie, F., Rutherford, L. & Marcinkiewicz, A. *Vitamin D status of Scottish adults: Results from the 2010 & 2011 Scottish Health Surveys*. *Scottish Health Surveys* https://www.foodstandards.gov.scot/downloads/Report_Final.pdf (2013).

For peer review only

12. Tremlett, H., Zhao, Y. & Devonshire, V. Natural history of secondary-progressive multiple sclerosis. *Mult. Scler.* **14**, 314–324 (2008).

13. Hobart, J., Lamping, D., Fitzpatrick, R., Riazi, A. & Thompson, A. The multiple sclerosis impact scale (MSIS-29) a new patient-based outcome measure. *Brain* **124**, 962–973 (2001).

14. Richardson, L. C., Wingo, P. A., Zack, M. M., Zahran, H. S. & King, J. B. Health-related quality of life in cancer survivors between ages 20 and 64 years: Population-based estimates from the behavioral risk factor surveillance system. *Cancer* **112**, 1380–1389 (2008).

15. Learmonth, Y. C., Motl, R. W., Sandroff, B. M., Pula, J. H. & Cadavid, D. Validation of patient determined disease steps (PDDS) scale scores in persons with multiple sclerosis. *BMC Neurol.* **13**, (2013).

16. Krupp, L. B., Larocca, N. G., Muir Nash, J. & Steinberg, A. D. The fatigue severity scale:

Application to patients with multiple sclerosis and systemic lupus erythematosus. *Arch. Neurol.* **46**, 1121–1123 (1989).

17. Spitzer, R. L., Kroenke, K., Williams, J. B. W. & Löwe, B. A brief measure for assessing generalized anxiety disorder: The GAD-7. *Arch. Intern. Med.* **166**, 1092–1097 (2006).

18. Kroenke, K., Krebs, E. E. & Bair, M. J. Pharmacotherapy of chronic pain: a synthesis of recommendations from systematic reviews. *Gen. Hosp. Psychiatry* **31**, 206–19 (2009).

19. Cohen, R. A., Kessler, H. R. & Fischer, M. The extended disability status scale (EDSS) as a predictor of impairments of functional activities of daily living in multiple sclerosis. *J. Neurol. Sci.* **115**, 132–135 (1993).

20. Polman, C. H. & Rudick, R. A. The multiple sclerosis functional composite a clinically meaningful measure of disability. *Neurology* vol. 74 (2010).

21. O 'gorman, C., Lucas, R. & Taylor, B. Environmental Risk Factors for Multiple Sclerosis: A Review with a Focus on Molecular Mechanisms. *Int. J. Mol. Sci* **13**, 11718–11752 (2012).

22. Hedström, A. K. *et al.* The interaction between smoking and HLA genes in multiple sclerosis: replication and refinement. *Eur. J. Epidemiol.* **32**, 909–919 (2017).

23. Newland, P., Starkweather, A. & Sorenson, M. Central fatigue in multiple sclerosis: a review of the literature. *Journal of Spinal Cord Medicine* vol. 39 386–399 (2016).

24. Petzold, A. *et al.* Optical coherence tomography in multiple sclerosis: A systematic review and meta-analysis. *The Lancet Neurology* vol. 9 921–932 (2010).

25. Dyment, D. A., Yee, I. M. L., Ebers, G. C., Sadovnick, A. D. & Canadian Collaborative Study Group. Multiple sclerosis in stepsiblings: recurrence risk and ascertainment. *J. Neurol. Neurosurg. Psychiatry* **77**, 258–9 (2006).

26. Sawcer, S. *et al.* Genetic risk and a primary role for cell-mediated immune mechanisms in multiple sclerosis. *Nature* **476**, 214–219 (2011).

27. De Bakker, P. I. W. *et al.* A high-resolution HLA and SNP haplotype map for disease association studies in the extended human MHC. *Nat. Genet.* **38**, 1166–1172 (2006).

28. Baranzini, S. E. & Oksenberg, J. R. The Genetics of Multiple Sclerosis: From 0 to 200 in 50 Years. *Trends Genet.* **33**, 960–970 (2017).

29. Goris, A. *et al.* Genetic variants are major determinants of CSF antibody levels in multiple sclerosis. *Brain* **138**, 632–643 (2015).

For peer review only

30. Poskanzer, D. C., Prenney, L. B., Sheridan, J. L. & Kondy, J. Y. *Multiple sclerosis in the Orkney and Shetland Islands I: Epidemiology, clinical factors, and methodology. Journal of Epidemiology and Community Health* vol. 34 http://jech.bmj.com/ (1980).

31. International Multiple Sclerosis Genetics Consortium *et al.* Risk Alleles for Multiple Sclerosis Identified by a Genomewide Study. *N. Engl. J. Med.* **357**, 851–862 (2007).

32. Alcina, A. *et al.* Multiple sclerosis risk variant HLA-DRB1*1501 associates with high expression of DRB1 gene in different human populations. *PLoS One* **7**, (2012).

33. Benešová, Y. *et al.* Association of HLA-DRB1*1501 tagging rs3135388 gene polymorphism with multiple sclerosis. *J. Neuroimmunol.* **255**, 92–96 (2013).

34. George, M. F. *et al.* Multiple sclerosis risk loci and disease severity in 7,125 individuals from 10 studies. *Neurol. Genet.* **2**, (2016).

35. Schmidt, H., Williamson, D. & Ashley-Koch, A. Human Genome Epidemiology (HuGE) Review HLA-DR15 Haplotype and Multiple Sclerosis: A HuGE Review. doi:10.1093/aje/kwk118.

36. Dean, G., Goodall, J. & Downie, A. The prevalence of multiple sclerosis in the Outer Hebrides compared with north-east Scotland and the Orkney and Shetland Islands. *J. Epidemiol. Community Health* **35**, 110–113 (1981).

37. Roberts, D. F., Roberts, M. J. & Poskanzer, D. C. Genetic analysis of multiple sclerosis in Shetland. *J. Epidemiol. Community Health* **37**, 281–285 (1983).

38. Shepherd, D. I. & Downie, A. W. Prevalence Of Multiple Sclerosis In North-East Scotland. *BMJ* **2**, 314–316 (1978).

39. Bihrmann, K. *et al.* Small-scale geographical variation in multiple sclerosis: A case-control study using Danish register data 1971-2013. *Mult. Scler. Relat. Disord.* **23**, 40–45 (2018).

40. Kurtzke, J. F. Geography in Multiple Sclerosis. *J. Neurol* **215**, 1–26 (1977).

ACKNOWLEDGEMENTS

This study is indebted to the FutureMS participants. We would like to thank non-author contributors of the FutureMS Consortium and clinical collaborators within neurology departments across NHS Scotland. With thanks to FutureMS, hosted by Precision Medicine Scotland Innovation Centre [PMSIC] and funded by a grant from the Scottish Funding Council to Precision Medicine Scotland Innovation Centre (PMS IC) and Biogen Idec Ltd Insurance. PK is supported by ECAT/Wellcome fellowship. Many thanks to Olivia Fleming for critical review of the manuscript.

For peer review only

TABLES

	FutureMS	Scottish Multiple Sclerosis Register	p-value
**Female (n, %)**	325 (73.9%)	1916 (71.3%)	
**Male (n, %)**	115 (26.1%)	772 (28.7%)	.26
**SIMD Quintile**			
**1 (most deprived)**	71 (16.1%)	671 (18.2%)	
**2**	75 (17.0%)	718 (19.5%)	
**3**	84 (19.1%)	719 (19.5%)	
**4**	102 (23.2%)	802 (21.8%)	
**5 (least deprived)**	108 (24.5%)	770 (20.9%)	.29
**Age at symptom onset (mean (range))**	33.8 (50.69)	Data not available	
**Age at diagnosis (mean (range))**	37.7 (48.3)	38.1 (64.8)	.49

Table 1. Comparison of the baseline demographics. Data for persons with RRMS recorded in between Jan 1st 2010 and Dec 31st 2017 Scottish MS Register[4]. P-values for test hypothesis that there is no difference in proportions or means, between the two study populations, calculated by Chi-squared test (proportions) or two-sided t-test (age).

For peer review only

Variable	Baseline (n = 440)	Month 12 (n = 392)
**BMI (mean, SD)**	27.9 (6.9)	28.0 (6.8)
**In employment (%)**	82.9%	82.9%
**Taking Vitamin Supplements (n, (%))**	353 (81.3%)	340 (89.2%)
**Smoking status**		
**Current (n, (%))**	64 (14.7%)	51/391 (13.0%)
**Ever (n, (%))**	220 (50.7%)	196/391 (50.1%)
**Never (n, (%))**	214 (49.3%)	195/391 (49.9%)
**Unknown**	6	1
**PASAT-3 (mean, (SD))**	42.4 (14.5)	44.7 (14.2)
**SDMT (mean, (SD))**	59.0 (11.5)	60.6 (12.8)
**9 hole peg test, seconds (mean, max)**	21.2 (64.8)	20.5 (121.1)
**Timed 25-foot walk, seconds (mean, max)**	5.63 (19.1)	5.55 (40.0)
**EDSS (median, (IQR))**	2.0 (1.5)	2.5 (1)
**MSFC (mean, (SD))**	-0.04 (0.86)	0.13 (0.94)
**PHQ-9 (median, IQR)**	7 (9)	4 (7)
**GAD-7 (median, IQR)**	4 (6)	4 (6)
**FSS (median, IQR)**	35 (23)	35 (30)
**MSIS-29 (median, IQR)**	47 (28)	44 (27)
**MSSS (median, range)**	5.58 (9.76)	5.58 (9.78)
**ARMSS (median, range)**	4.55 (9.3)	4.93 (9.15)
**T2 white matter hyperintensities (Median (range))**	0.69% (0.04 – 8.5)	0.82% (0.07 - 7.2)

Table 2 – Summary of baseline and month 12 clinical, radiological, and lifestyle measures.

For peer review only

SNP	Gene	Chr	Risk Allele		AA n (%)	AG n (%)	GG n (%)	RAF	Adjusted OR (95% CI)*	p-value
Rs3135388	HLA-DRB1*15:01	6	**A** (A/G)	Cases (n = 428)	42 (9.8)	204 (47.7)	182 (42.5)	0.34	3.90 (2.50 – 6.34)	8.8×10^{-9}
				Controls (n = 100)	3	18	79	0.12		

*Additive model, adjusted for sex.

*Table 3: Frequency of HLA-DRB1*15:01 in FutureMS Cases and Controls. OR assume an additive logistic regression model. P-values adjusted for multiple testing by Bonferroni method. RAF = Risk Allele Frequency. SNPs named relative to forward strand Gb37.*

For peer review only

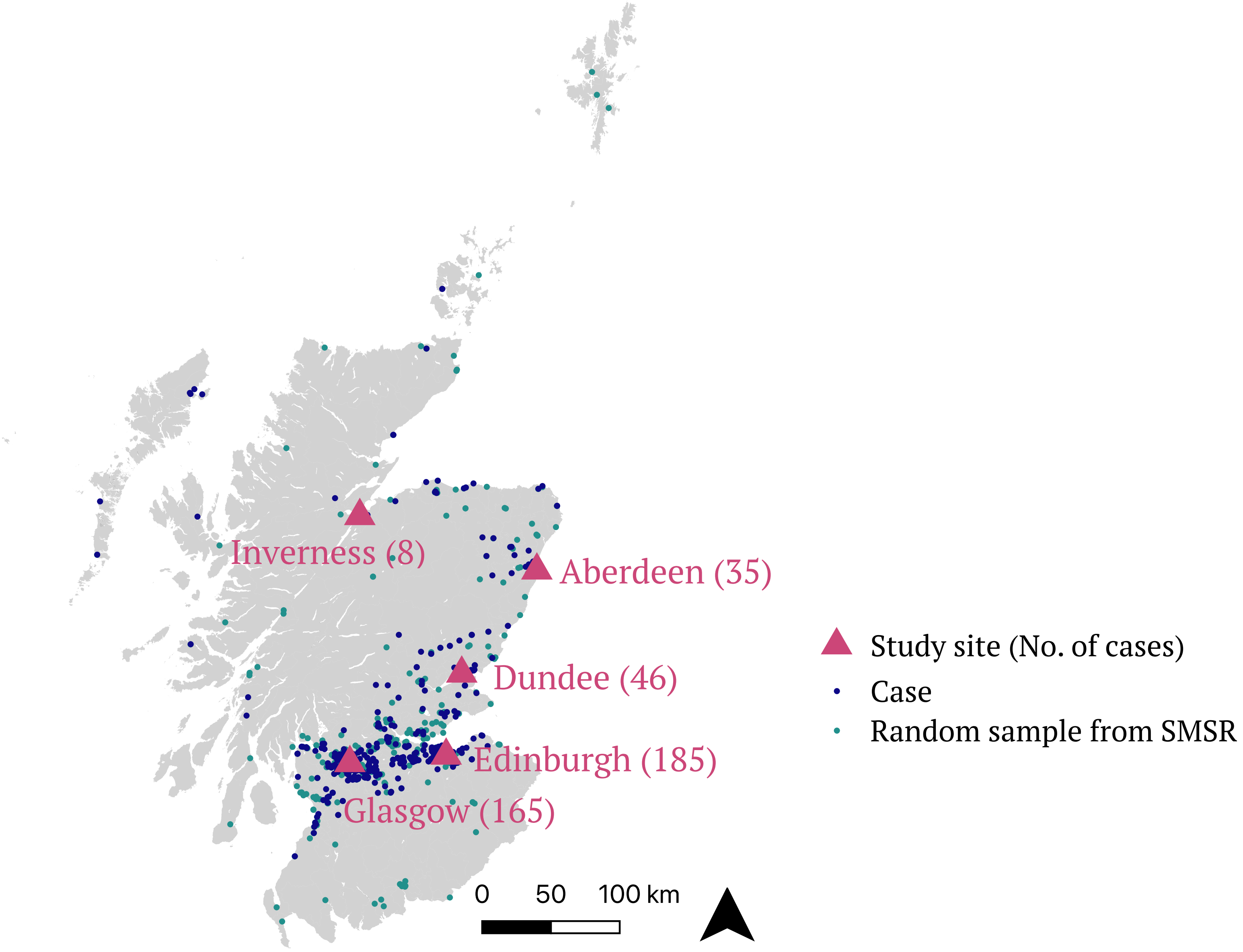
Inverness (8)
Aberdeen (35)
Dundee (46)
Edinburgh (185)
Glasgow (165)
0 50 100 km
Study site (No. of cases)
Case
Random sample from SMSR

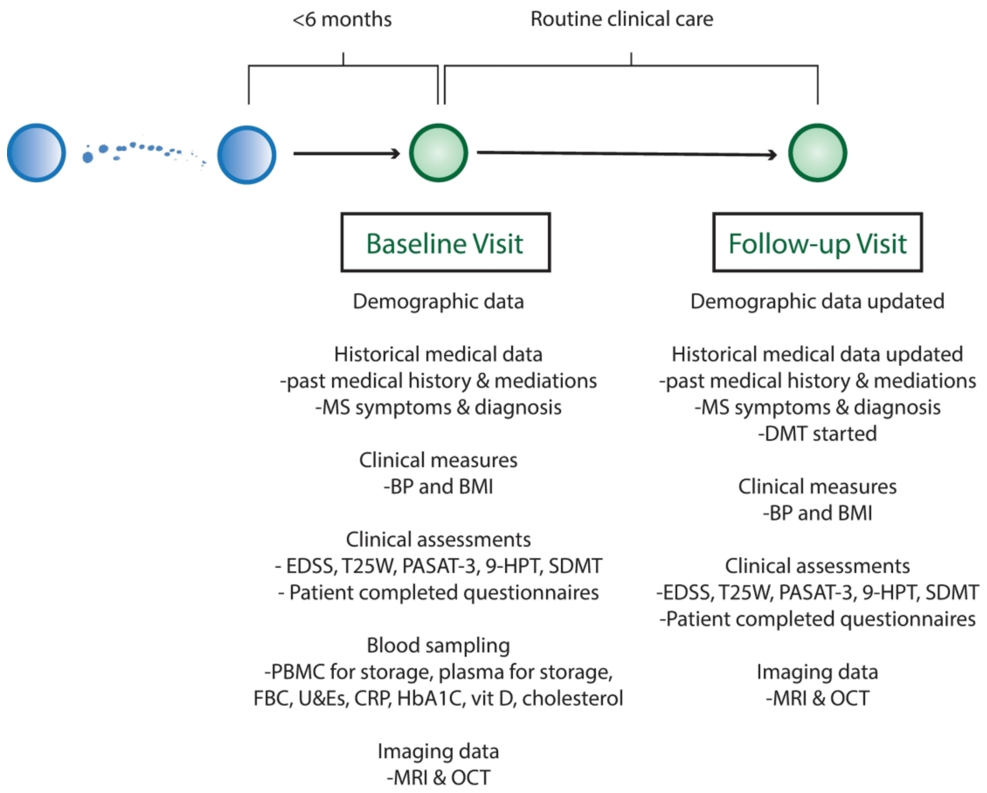

Figure 2: FutureMS cohort design. BP – blood pressure; BMI – Body mass index; EDSS – Extended disability status scale; T25W – Timed 25foot walk test; PASAT – paced auditory serial addition test 3; 9HPT – nine hole peg test; SDMT – symbol digit modality test; PBMC – Peripheral blood mononuclear cells; FBC – full blood count; U&E – urea and electrolytes and renal function tests; LFT – liver function test panel; CRP – C-Reactive protein; HbA1C – glycosylated haemoglobin A1C; vit D – 25-OH-vitamin D; MRI – magnetic resonance imaging; OCT – optical coherence tomography.

239x192mm (300 x 300 DPI)

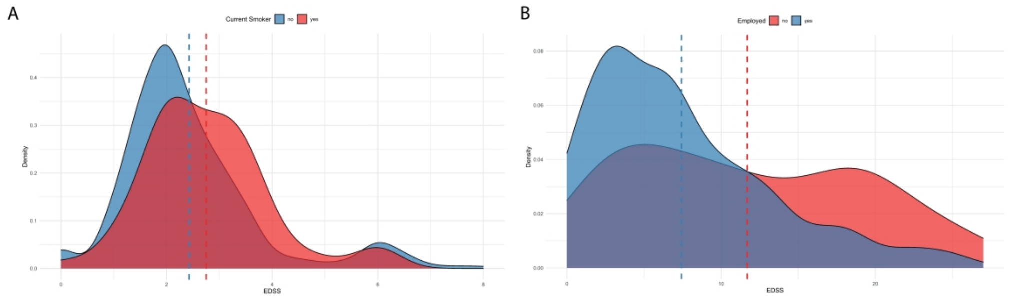

Figure 3: Density plots stratifying the cohort at baseline visit. A – Distribution of EDSS (a measure of physical disability) by smoking status. B – Evidence of greater burden of depression as detected by PHQ9 in those who are unemployed at baseline.

443x130mm (300 x 300 DPI)

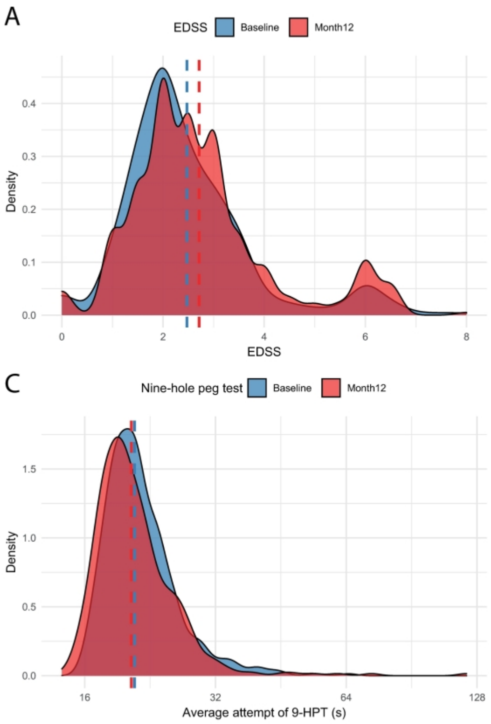

Figure 4: Physical measures of disability across the cohort at baseline and month 12. T25FtWT X axis is in seconds, 9-HPT is the mean between hands of the mean of two attempts with each hand and is a measure of upper limb disability measured in seconds (longer time reflects less dexterity). EDSS is an ordinal scale where higher scores reflect greater disability. MSFC is a continuous scale(z-score), participants who are unable to walk are arbitrarily attributed very low Z-scores for the walking component of their test (-13.7) as per published instructions. This gives a long negative tail to the distribution as the -13.7 is chosen to allow for the cohort to progress in disability with time and still capture variance in walking ability.

310x227mm (300 x 300 DPI)

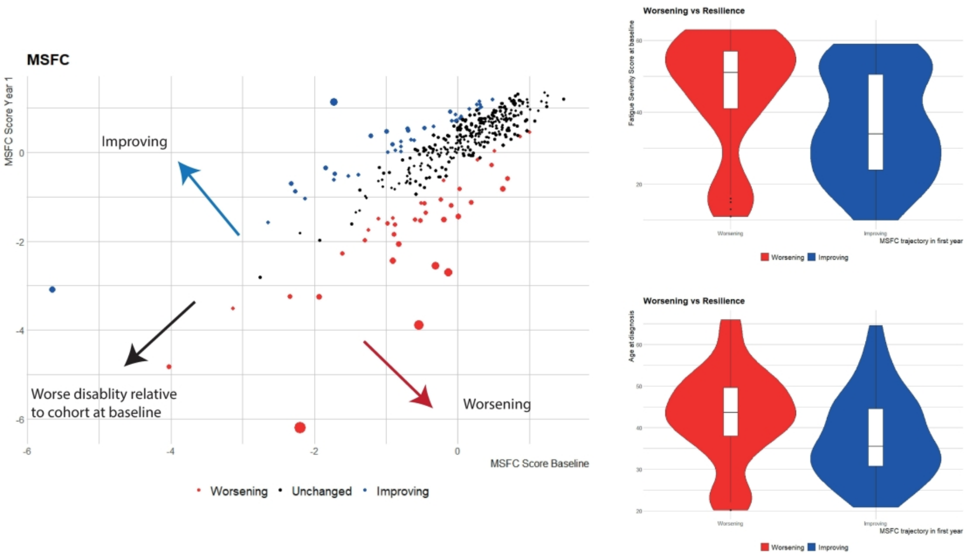

Figure 5: Individual level change in physical disability between the waves. Circle size reflects size of difference between MSFC measurements between study visits (squared residual from least squares regression line of MSFC at year one on MSFC at year two). Outlier groups defined as above the 90th and below the 10th centile for regression residual.

278x170mm (300 x 300 DPI)

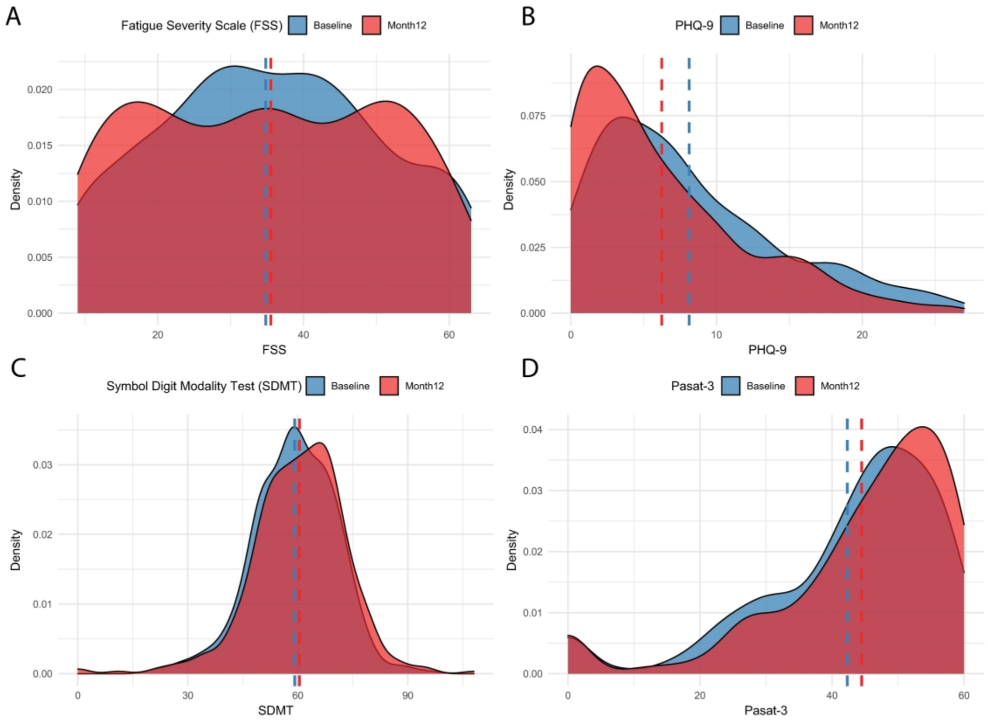

Figure 6 – Fatigue Severity Score, PHQ-9 screening tool for depression, Symbol Digit Modality Tool, Paced Serial Addition Tool. Higher scores on Fatigue Severity Scale indicate worse fatigue. Higher scores on PHQ-9 indicate risk of depression. Higher scores on PASAT and SDMT indicate better performance on cognition testing and so less impairment.

310x227mm (300 x 300 DPI)

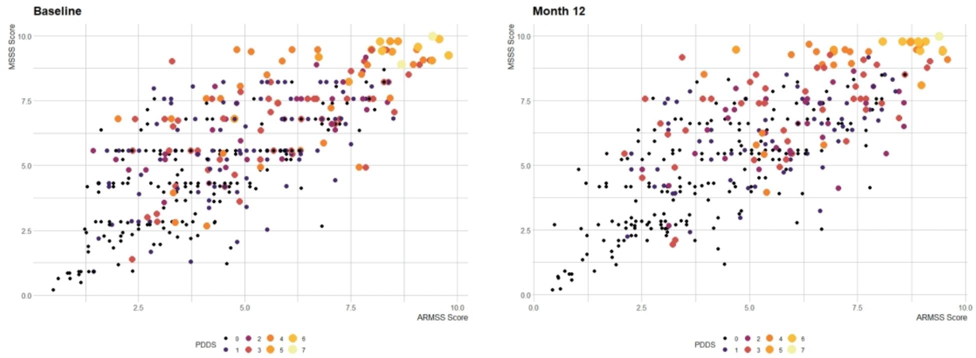

Figure 7: size and colour of the points reflects the patient determined diseases steps. In these figures, points are study individuals, and the size and colour of the points are scaled using the PDDS (range 0 – 7, where 7 is most severe).

535x210mm (300 x 300 DPI)

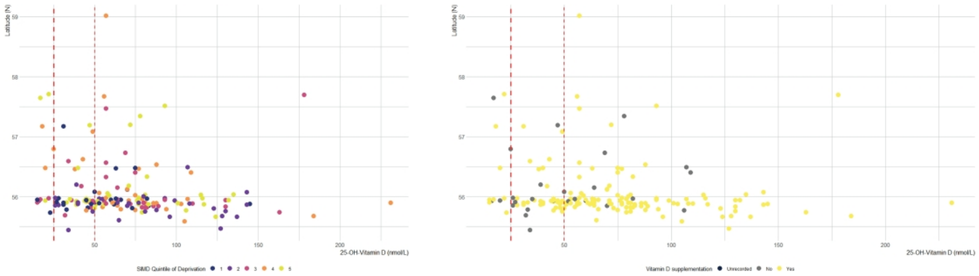

Figure 8: Vitamin D and Latitude of residence show no relationship for the subset (n = 186) of participants with 25-OH-Vitamin D measured at baseline visit (>80% of participants recorded as taking vitamin D supplements). Univariate linear regression shows no relationship with latitude of residence or quintile of socioeconomic deprivation. SIMD quintile of deprivation (1 = most deprived, 5 = least deprived).

697x210mm (300 x 300 DPI)

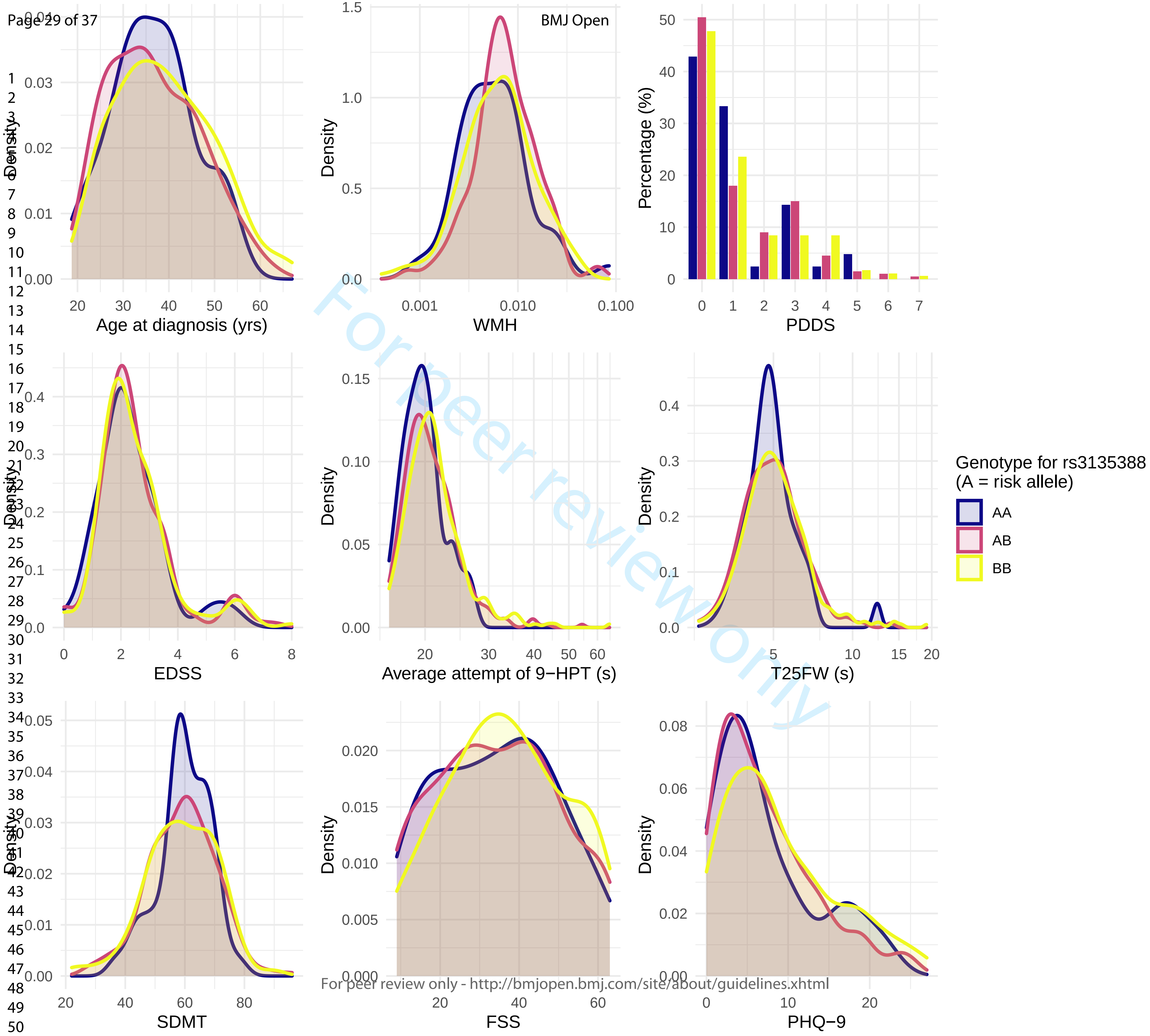

For peer review only

SUPPLEMENTARY FIGURES AND TABLES

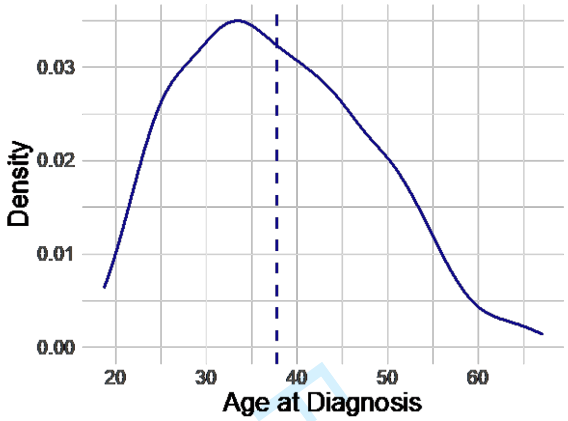

Figure S1. Demonstrating truncation at extremes of age (particularly <18) due to inclusion criteria. Will mention in limitations.

For peer review only

ASSESSMENT	Baseline	Year 1	Year 5 (in planning)
Informed Consent	X		X
Demographics	X		
Medical History	X	X	X
Social History	X	X	X
Family History	X	X	X
Migration History			X
Relapse/Progression History	X	X	X
Medication History	X	X	X
Structured Neurological Examination	X	X	X
BMI, Height, Weight, BP	X	X	X
Measures of Physical Disability (EDSS, T25FTWT, 9HPT)	X	X	X
Cognitive tests (PASAT-3, SDMT)	X	X	X
PHQ-9 and GAD-7	X	X	X
MSIS-29	X	X	X
Fatigue Severity Score	X	X	X
Patient Derived Disease Severity Score	X	X	X
Visual Acuity	X	X	X
Standard MRI Protocol	X	X	X
Disease Modifying Therapy History	N/A	X	X
Lymphocyte subsets stored (CD4, CD8, CD20, CD??)	X		
DNA Genotyping	X		
Advanced MRI Imaging (SS3)	X	X	X

For peer review only

Optical Coherence Tomography (SS4)	X	X	X

Table S1. Summary of study visit.

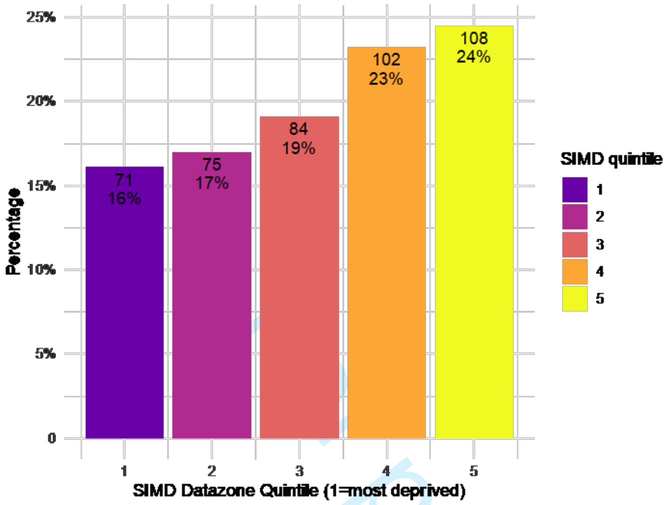

Figure S2. SIMD quintiles.

For peer review only

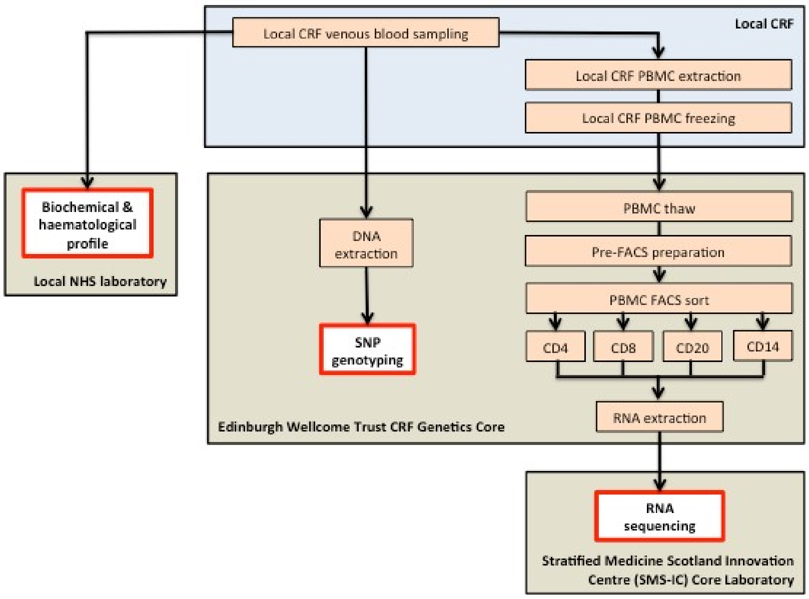

Figure S3: Schematic explaining laboratory pathway for tissue collection for biomarker processing.

For peer review only

	With PBMCs	FACs sorted	Total
Edinburgh (01)	184	184	185
Glasgow (02)	163	137	165
Dundee (03)	46	46	46
Aberdeen (04)	35	32	35
Inverness (05)	8	6	8
Controls	100	78	103
**Totals (patients only)**	**436**	**405**	**439**
**Totals**	**536**	**483**	**542**

Supplementary table 2: Summary of numbers of FutureMS study subjects and controls with peripheral blood mononuclear cells available for analysis.

For peer review only

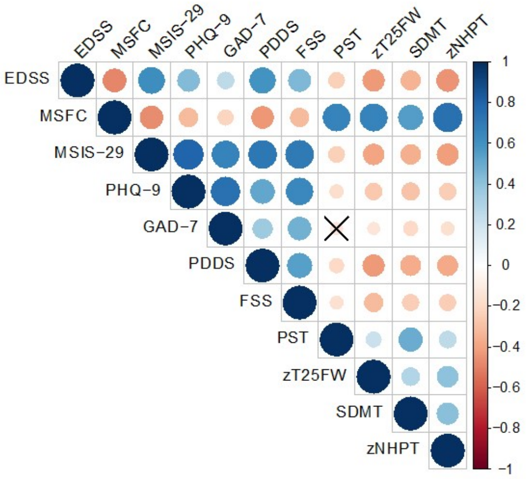

Figure S4. Correllelogram of clinical measures at baseline visit.

For peer review only

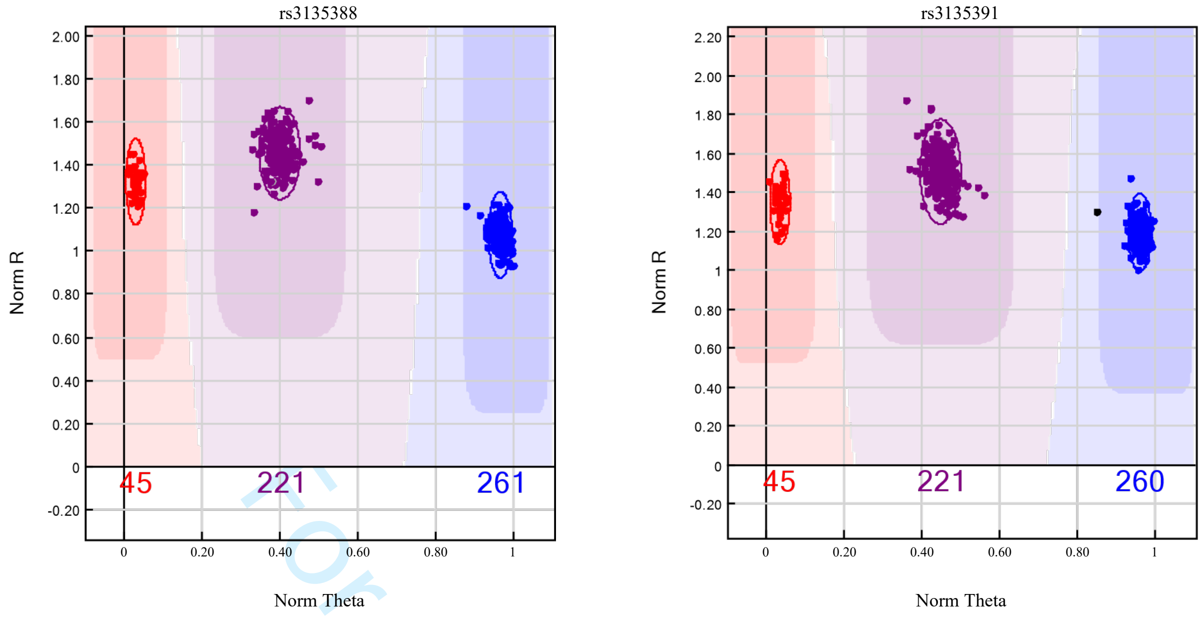

*Supplementary Figure 5. Demonstration of two SNPs in near perfect linkage disequilibrium from the FutureMS cohort demonstrating clear separation of clusters resulting in high confidence of genotype calls. These two SNPs mark HLA-DRB1*15:01, which contributes the largest single gene effect for multiple sclerosis.*

	Parent/Grandparent	% of Parent				% of Grandparent			
		Mean	SD	LQ10	UQ10	Mean	SD	LQ10	UQ10
Single cells	Events	56.05	15.98	35.60	75.00				
Viable (7AAD -ve)	Single cells	91.77	10.11	97.80	84.93.				
CD14+ Monocyes	CD3-/viable cells	22.03	11.67	34.30	10.40				
CD19+ B cells	CD3-/viable cells	8.11	3.69	4.08	12.9				
CD4+ T cells	CD3+/viable cells	69.44	11.67	81.47	57.00	38.97	10.48	51.27	26.56
CD8+ T cells	CD3+/viable cells	20.27	8.31	30.37	11.22	20.27	8.31	30.37	11.22

Supplementary table 3. Proportion of PBMCs expressing markers for monocytes, B cells, helper and cytotoxic t cells in the peripheral blood mononuclear cells donated by patients to thestudy.

For peer review only

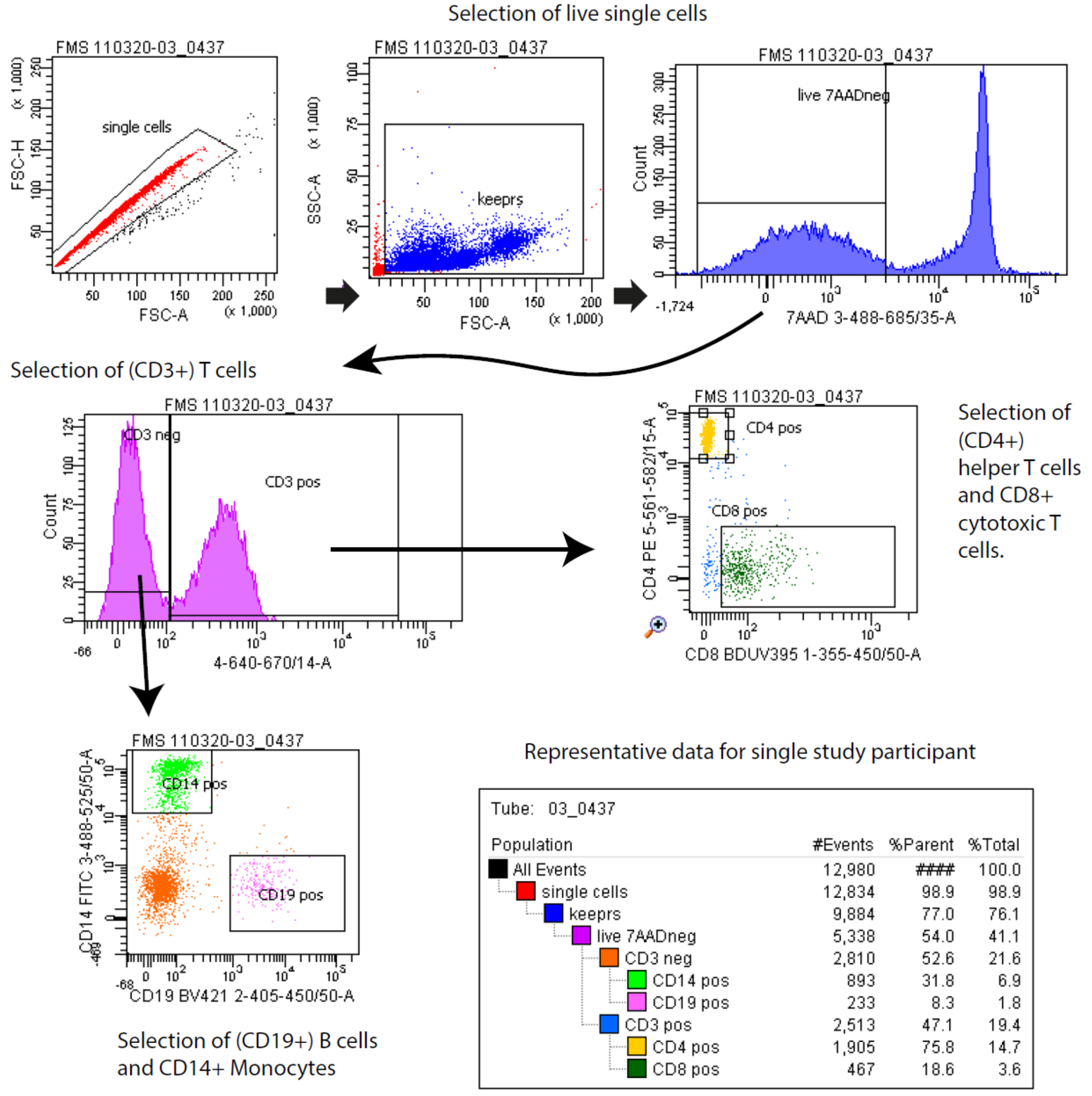

Population	#Events	%Parent	%Total
All Events	12,980	####	100.0
single cells	12,834	98.9	98.9
keeprs	9,884	77.0	76.1
live 7AADneg	5,338	54.0	41.1
CD3 neg	2,810	52.6	21.6
CD14 pos	893	31.8	6.9
CD19 pos	233	8.3	1.8
CD3 pos	2,513	47.1	19.4
CD4 pos	1,905	75.8	14.7
CD8 pos	467	18.6	3.6

Supplementary figure 6. Example of the fluorescence-activated cell sorting undertaken for a representative member of the FMS cohort. CD3 – T cell marker. CD4+ helper T cells, CD8+ cytotoxic T cells. CD19+ B cell marker and CD14+ monocyte marker. 7AAD – 7-Aminoactinomycin D uptake associated with loss of cell viability.

STROBE Statement—Checklist of items that should be included in reports of ***cohort studies***

	Item No	Recommendation	Page No
**Title and abstract**	1	(*a*) Indicate the study's design with a commonly used term in the title or the abstract	1
		(*b*) Provide in the abstract an informative and balanced summary of what was done and what was found	1
**Introduction**			
Background/rationale	2	Explain the scientific background and rationale for the investigation being reported	2
Objectives	3	State specific objectives, including any prespecified hypotheses	2
**Methods**			
Study design	4	Present key elements of study design early in the paper	3
Setting	5	Describe the setting, locations, and relevant dates, including periods of recruitment, exposure, follow-up, and data collection	3
Participants	6	(*a*) Give the eligibility criteria, and the sources and methods of selection of participants. Describe methods of follow-up	3
		(*b*) For matched studies, give matching criteria and number of exposed and unexposed	N/A
Variables	7	Clearly define all outcomes, exposures, predictors, potential confounders, and effect modifiers. Give diagnostic criteria, if applicable	3
Data sources/ measurement	8*	For each variable of interest, give sources of data and details of methods of assessment (measurement). Describe comparability of assessment methods if there is more than one group	3
Bias	9	Describe any efforts to address potential sources of bias	5
Study size	10	Explain how the study size was arrived at	3
Quantitative variables	11	Explain how quantitative variables were handled in the analyses. If applicable, describe which groupings were chosen and why	5
Statistical methods	12	(*a*) Describe all statistical methods, including those used to control for confounding	6
		(*b*) Describe any methods used to examine subgroups and interactions	6
		(*c*) Explain how missing data were addressed	6
		(*d*) If applicable, explain how loss to follow-up was addressed	6
		(*e*) Describe any sensitivity analyses	6
**Results**			
Participants	13*	(a) Report numbers of individuals at each stage of study—eg numbers potentially eligible, examined for eligibility, confirmed eligible, included in the study, completing follow-up, and analysed	4
		(b) Give reasons for non-participation at each stage	4
		(c) Consider use of a flow diagram	Fig2
Descriptive data	14*	(a) Give characteristics of study participants (eg demographic, clinical, social) and information on exposures and potential confounders	Tab1
		(b) Indicate number of participants with missing data for each variable of interest	5
		(c) Summarise follow-up time (eg, average and total amount)	Fig2
Outcome data	15*	Report numbers of outcome events or summary measures over time	Results

For peer review only

For peer review only - http://bmjopen.bmj.com/site/about/guidelines.xhtml

Main results	16	(*a*) Give unadjusted estimates and, if applicable, confounder-adjusted estimates and their precision (eg, 95% confidence interval). Make clear which confounders were adjusted for and why they were included	N/A
		(*b*) Report category boundaries when continuous variables were categorized	N/A
		(*c*) If relevant, consider translating estimates of relative risk into absolute risk for a meaningful time period	N/A
Other analyses	17	Report other analyses done—eg analyses of subgroups and interactions, and sensitivity analyses	N/A
**Discussion**			
Key results	18	Summarise key results with reference to study objectives	Discussion
Limitations	19	Discuss limitations of the study, taking into account sources of potential bias or imprecision. Discuss both direction and magnitude of any potential bias	Discussion
Interpretation	20	Give a cautious overall interpretation of results considering objectives, limitations, multiplicity of analyses, results from similar studies, and other relevant evidence	Discussion
Generalisability	21	Discuss the generalisability (external validity) of the study results	Discussion
**Other information**			
Funding	22	Give the source of funding and the role of the funders for the present study and, if applicable, for the original study on which the present article is based	Funding statement

*Give information separately for exposed and unexposed groups.

Note: An Explanation and Elaboration article discusses each checklist item and gives methodological background and published examples of transparent reporting. The STROBE checklist is best used in conjunction with this article (freely available on the Web sites of PLoS Medicine at http://www.plosmedicine.org/, Annals of Internal Medicine at http://www.annals.org/, and Epidemiology at http://www.epidem.com/). Information on the STROBE Initiative is available at http://www.strobe-statement.org.

For peer review only

BMJ Open

FutureMS Cohort Profile: A Scottish Multi-Centre Inception Cohort Study of Relapsing-Remitting Multiple Sclerosis.

Journal:	*BMJ Open*
Manuscript ID	bmjopen-2021-058506.R1
Article Type:	Cohort profile
Date Submitted by the Author:	06-Apr-2022
Complete List of Authors:	Kearns, Patrick; The University of Edinburgh Centre for Clinical Brain Sciences, Anne Rowling Regenerative Neurology Clinic; The University of Edinburgh MRC Human Genetics Unit, Chromatin Lab, Genome Regulation Section<br>Martin, Sarah; The University of Edinburgh Centre for Clinical Brain Sciences; NHS Greater Glasgow and Clyde, Institute of Clinical Neurosciences<br>Chang, Jessie; The University of Edinburgh Centre for Clinical Brain Sciences, Anne Rowling Regenerative Neurology Clinic<br>Meijboom, Rozanna; The University of Edinburgh Centre for Clinical Brain Sciences, Anne Rowling Regenerative Neurology Clinic<br>York, Elizabeth; The University of Edinburgh Centre for Clinical Brain Sciences, Anne Rowling Regenerative Neurology Clinic<br>Chen, Yingdi; The University of Edinburgh Centre for Clinical Brain Sciences, Anne Rowling Regenerative Neurology Clinic<br>Weaver, Christine; The University of Edinburgh Centre for Clinical Brain Sciences, Anne Rowling Regenerative Neurology Clinic<br>Stenson, Amy; The University of Edinburgh Centre for Clinical Brain Sciences, Anne Rowling Regenerative Neurology Clinic<br>Hafezi, Katarzyna; Wellcome Trust Clinical Research Facility Edinburgh<br>Thomson, Stacey; The University of Edinburgh MRC Institute of Genetics and Molecular Medicine<br>Freyer, Elizabeth; The University of Edinburgh MRC Institute of Genetics and Molecular Medicine<br>Murphy, Lee; Wellcome Trust Clinical Research Facility Edinburgh<br>Harroud, Adil; Weill Institute of Clinical Neuroscience, Department of Neurology<br>Foley, Peter; The University of Edinburgh Centre for Clinical Brain Sciences, Anne Rowling Regenerative Neurology Clinic<br>Hunt, David; The University of Edinburgh Centre for Clinical Brain Sciences, Anne Rowling Regenerative Neurology Clinic<br>McLeod, Margaret; Aberdeen Royal Infirmary, Department of Neurology<br>O'Riordan, Jonathon; University of Dundee Division of Neuroscience, Tayside Centre for Clinical Neurosciences<br>Carod-Artal, F.J.; Raigmore Hospital, Department of Neurology<br>MacDougall, Niall; Wishaw General Hospital, Department of Neurology<br>Baranzini, Sergio; Weill Institute of Clinical Neuroscience, Department of Neurology<br>Waldman, Adam; The University of Edinburgh Centre for Clinical Brain Sciences<br>Connick, Peter; University of Edinburgh, Centre for Clinical Brain

	Sciences<br>Chandran, Siddharthan ; The University of Edinburgh Centre for Clinical Brain Sciences, Anne Rowling Regenerative Neurology Clinic
**Primary Subject Heading**:	Neurology
Secondary Subject Heading:	Epidemiology, Genetics and genomics
Keywords:	Neurology < INTERNAL MEDICINE, EPIDEMIOLOGY, Neurogenetics < NEUROLOGY, Multiple sclerosis < NEUROLOGY, Neuroradiology < NEUROLOGY, INTERNAL MEDICINE

I, the Submitting Author has the right to grant and does grant on behalf of all authors of the Work (as defined in the below author licence), an exclusive licence and/or a non-exclusive licence for contributions from authors who are: i) UK Crown employees; ii) where BMJ has agreed a CC-BY licence shall apply, and/or iii) in accordance with the terms applicable for US Federal Government officers or employees acting as part of their official duties; on a worldwide, perpetual, irrevocable, royalty-free basis to BMJ Publishing Group Ltd ("BMJ") its licensees and where the relevant Journal is co-owned by BMJ to the co-owners of the Journal, to publish the Work in this journal and any other BMJ products and to exploit all rights, as set out in our licence.

The Submitting Author accepts and understands that any supply made under these terms is made by BMJ to the Submitting Author unless you are acting as an employee on behalf of your employer or a postgraduate student of an affiliated institution which is paying any applicable article publishing charge ("APC") for Open Access articles. Where the Submitting Author wishes to make the Work available on an Open Access basis (and intends to pay the relevant APC), the terms of reuse of such Open Access shall be governed by a Creative Commons licence – details of these licences and which Creative Commons licence will apply to this Work are set out in our licence referred to above.

Other than as permitted in any relevant BMJ Author's Self Archiving Policies, I confirm this Work has not been accepted for publication elsewhere, is not being considered for publication elsewhere and does not duplicate material already published. I confirm all authors consent to publication of this Work and authorise the granting of this licence.

For peer review only

FutureMS Cohort Profile: A Scottish Multi-Centre Inception Cohort Study of Relapsing-Remitting Multiple Sclerosis.

AUTHORS:

P.K.A. Kearns*[1,2,3,4], S.J. Martin[2,5], J. Chang[1,2], R. Meijboom[2], E.N. York[2], Y. Chen[1,2], C. Weaver[1,2], A. Stenson[1,2], K. Hafezi[6], S. Thomson[3], E. Freyer[3], L. Murphy[6], A. Harroud[7], P.Foley[1,2], D. Hunt[1,2], M. McLeod[8], J. O'Riordan[9], F.J. Carod-Artal[10], N.J.J. MacDougall[1,11], S.E. Baranzini[7], A.D. Waldman[2], P. Connick[1,2,4] S. Chandran*[1,2,4]

Affiliations:

1. Anne Rowling Regenerative Neurology Clinic, University of Edinburgh, UK
2. Centre for Clinical Brain Sciences, University of Edinburgh, UK
3. Institute of Genetics and Cancer, University of Edinburgh, UK
4. Department of Clinical Neurosciences, Edinburgh Royal Infirmary, NHS Lothian, UK
5. Department of Neurology, NHS Greater Glasgow and Clyde, UK
6. Wellcome Trust Clinical Research Facility, University of Edinburgh, UK
7. Weill Institute for Clinical Neuroscience, Department of Neurology, University of California San Francisco, USA.
8. Department of Neurology, Aberdeen Royal Infirmary, NHS Grampian, UK
9. Tayside Centre for Clinical Neurosciences, University of Dundee, UK
10. Department of Neurology, Raigmore hospital, NHS Highlands, UK
11. Department of Neurology, University Hospital Hairmyres, NHS Lanarkshire, UK

Corresponding authors*: Dr Patrick Kearns (P.Kearns@ed.ac.uk) & Prof S.Chandran (Siddharthan.chandran@ed.ac.uk)

ABSTRACT:

Purpose: Multiple sclerosis (MS) is an immune-mediated, neuroinflammatory disease of the central nervous system and in industrialised countries is the most common cause of progressive neurological disability in working age persons. Whilst treatable, there is substantial interindividual heterogeneity in disease activity and response to treatment. Currently, the ability to predict at diagnosis who will have a benign, intermediate, or aggressive disease course is very limited. There is therefore a need for integrated predictive tools to inform individualised treatment decision-making.

Participants: Established with the aim of addressing this need for individualised predictive tools, FutureMS is a nationally representative, prospective observational cohort study of 440 adults with a new diagnosis of relapsing-remitting MS living in Scotland at the time of diagnosis between May 2016 and March 2019.

Findings to date: The study aims to explore the pathobiology and determinants of disease heterogeneity in MS and combines detailed clinical phenotyping with imaging, genetic and biomarker metrics of disease activity and progression. Recruitment, baseline assessment and follow up at year one is complete. Here we describe the cohort design and present a profile of the participants at baseline and one year of follow up.

For peer review only

Future plans: A third follow up wave for the cohort has recently begun at five years after first visit and a further wave of follow up is funded for year ten. Longer term follow up is anticipated thereafter.

Main strengths and limitations:

Strengths:

- To our knowledge, this is the largest inception cohort (n=440) of individuals prospectively recruited with Relapse-Remitting Multiple Sclerosis.
- Participants are recruited as soon as possible following diagnosis and before starting on disease modifying therapies and substantial effort has been made to minimise barriers to participation to recruit a nationally representative cohort.
- Alongside the collection of demographic, socio-economic, lifestyle and psychosocial data, state-of-the-art clinical, radiological, biomarker, immunological, and genetic/transcriptomic phenotyping has been conducted on all participants at baseline and samples biobanked.

Limitations

- Follow-up time is currently limited (each participant has been followed for only one year) and only two time points are available so trends in disease trajectory will not yet be fully apparent.
- Some findings of the study may not be generalisable beyond the population eligible for recruitment: adults, living in Scotland, diagnosed with relapsing-remitting MS, who have not commenced a DMT at baseline.

For peer review only

Introduction

Multiple sclerosis (MS) is the leading cause of progressive neurological disability in young and working age persons in middle- and high-income countries pathophysiologically combining immune-mediated neuroinflammatory demyelination and neurodegeneration[1]. The majority (85-90%) of incident cases have a relapsing-remitting disease course (RRMS) at onset, characterised by periods of clinical symptoms emerging and resolving. After a median of 20 years, the disease enters a phase of progressively accumulating irreversible disability called secondary progressive MS (SPMS). The other 10-15% of incident cases experience this progressive phase from onset (primary progressive MS [PPMS]). In the RRMS group, both inflammation and neuronal injury are present throughout the disease course, with multifocal inflammatory demyelination dominant in the relapsing phase and neurodegeneration the key pathological substrate of the progressive phase[1–3]. MS remains incurable and untreated typically results in accumulation of substantial disability and a 5–15-year reduction in life expectancy. The emergence of effective disease modifying therapies (DMTs) for the early phase of disease has transformed the outlook for people living with RRMS in recent years[4–11].

However, RRMS has a markedly heterogenous natural history: cases of aggressive and relatively indolent disease occur on a spectrum even in untreated individuals[3]. The ability to prognosticate for an individual is limited and reactive in practice, relying on retrospective radiological or clinical evidence of disease activity[12–15]. Informed treatment and lifestyle decision making by people newly diagnosed with MS requires predictive tools available at or close to the point of diagnosis. The increase in DMT options, some of which carry serious potential side effects, emphasises the urgent need for accurate and personalised prognostic tools.

Licensed DMTs are considered to be more effective at preventing neuroinflammation than at halting neurodegeneration[16]. However, neurodegeneration is challenging to measure over short time periods, complicating both early prediction and the measurement of intervention efficacy on this aspect of MS pathology. It may therefore be essential for personalised predictive tools to be capable of discriminating between different biological contributions to disability progression and to do so longitudinally. Early predictors and determinants of neuroinflammation may differ from those predicting the rate of neurodegeneration. Long-term longitudinal follow up of adequately-powered and representative clinical cohorts, starting as early as possible in the disease course, which are resourced to "deeply phenotype" participants, will be important in deconvoluting this complexity. These promise to make a substantial contribution towards achieving this personalised decision making.

FutureMS, described here, is one such cohort. The study is now fully recruited and the first follow-up wave at one year has been completed. FutureMS is a large (n = 440) prospective inception cohort of newly diagnosed persons with relapsing-remitting MS (RRMS) living in Scotland at the time of their diagnosis. With a high incidence of MS, a stable population of 5.4. million, low rates of migration, and a national single-payer universal healthcare system free at the point of use, Scotland offers an ideal setting for a long-term longitudinal study of MS[17].

The FutureMS study hypothesis is that inter-individual variability in disease activity in RRMS is determined and will be predictable by a combination of clinical, laboratory, imaging, and genetic parameters. The primary aim is to develop predictive tools for focal neuroinflammatory disease activity based on clinical, laboratory, magnetic resonance imaging (MRI) and genomic assessment in patients with RRMS. Secondary outcomes include the development of predictive tools for a) neurodegenerative disease activity, b) clinical measures of disease activity, and c) clinical measures of quality of life. The study is structured in cross-sectional waves. Study visits take place at baseline (within six months of diagnosis), at month 12 (baseline + 12 months), and further follow-up is now underway at five years (baseline +5 years). Long-term follow up is planned thereafter.

For peer review only

Future MS aims to reduce uncertainty in predicting an individual's disease trajectory, and to allow for more tailored and personalised care for persons living with MS (pwMS). This paper provides an overview of the study design and introduces a profile of the study participants.

Cohort description

Between May 2016 and March 2019, 440 adult patients (age ≥ 18 years) were recruited as a nationally representative incidence sample within six months of their diagnosis (median time since diagnosis at first study visit: 60 days, IQR: 61 days). To ensure a study cohort representative of the population of persons newly diagnosed with MS in Scotland, the study was designed to support inclusion of any adult newly diagnosed with RRMS wherever they may live in Scotland, aiming to establish both geographically and socioeconomically representative coverage of the Scottish mainland and islands.

[Figure 1 approx. here]

Participants were recruited from the five tertiary Scottish clinical neurology centres: 185 from Edinburgh (42.0%), 164 from Glasgow (37.3%), 46 from Dundee (10.5%), 35 from Aberdeen (8.0%), and 8 from Inverness (1.8%). This roughly reflects the geographic distribution of the population of Scotland, and the geographic incidence burden of MS[17] (Fig. 1). Analysis of the Scottish Multiple Sclerosis Register (SMSR)), a national incidence register with mandatory reporting at the time of referral for newly diagnosed persons, reveals that 45% of all persons diagnosed with RRMS in Scotland over this period were recruited to FutureMS. Comparison with the demographic characteristics from the SMSR suggests a broadly representative sample was recruited (Table 1)[17]. FutureMS participants were slightly younger on average, less represented at the extremes of age distribution (Fig. S1) and more likely to be female (all non-statistically significant tendencies). As has been observed in the SMSR data[17], there was a significant excess of persons living in affluent Scottish Index of Multiple Deprivation[5] (SIMD) quintiles relative to deprived quintiles (SMSR: $X^2 = 14.06$, 4d.f., $p<0.01$; and FutureMS: $X^2 = 12.2$, 4 d.f., $p < 0.05$) (Fig. S2).

Amongst FutureMS participants who listed their ethnicity, 426/440 (96.8%) recorded their primary ethnicity as White Scottish/British. For those with recorded ethnicity in the first eight years of the SMSR (2010-2018), the proportion recorded as Scottish, British or Irish was similar 862/919 (93.8%)[17].

[Table 1 approximately here]

Diagnostic inclusion criteria

Diagnosis in all cases was confirmed by the treating consultant neurologist as fulfilling the most recent McDonald Criteria[18,19]. Participants were referred by the treating clinical teams and must not have commenced on DMT prior to baseline assessment. They must have had capacity to give informed consent and had no contraindication to MR brain imaging at the time of their baseline visit.

Only patients with a diagnosis of RRMS were eligible for inclusion in FutureMS. Those with progressive disease at diagnosis were excluded. The rationale for this decision was that diagnosis of progressive forms of MS requires a period of observation of sustained progression. Further, epidemiological studies have suggested that relapsing and progressive forms of MS are not clearly

For peer review only

demarcated clinical entities, but rather, that they reflect different stages of the same disease with progressive forms of MS being later manifestations of the same disease process[20–23].

Controls for laboratory and biomarker studies

103 healthy volunteers were recruited from the Lothian area to donate blood and DNA for the biomarker and genetic analyses. These persons were age- and sex- frequency matched to the study population. All were recruited in the Anne Rowling Regenerative Neurology Clinic in Edinburgh, and so are mainly drawn from the surrounding Edinburgh and Lothian areas.

Study visits

Study visits were not in place of standard neurological care. Consequently, only the timing of the first visit is likely to be associated with temporal fluctuations in the participants' disease activity, as diagnosis is likely to be temporally linked to a clinical relapse. Subsequent visits at fixed intervals are expected to be as independent as possible of clinical disease activity. This cross-sectional aspect of the study design is expected to reduce clinically triggered follow up biases in future waves of the study.

All clinical management decisions were made by the treating team. Participation in FutureMS is not a barrier to participating in any other research study including interventional trials, and we anticipate that a substantial number of participants will choose to engage with other research studies.

[Figure 2 approximately here]

Clinical and demographic data collection

Demographic and clinical variables collected at baseline included date of birth, sex, ethnicity, occupation, comorbidities, medication history (including 'over the counter' and supplements), and family history. Data pertaining to the diagnosis of RRMS (description of initial symptoms, number of clinical relapses, hospitalisations, and steroid use) were recorded at baseline visit, and all data were also updated at the twelve-month review (Fig. 2 and Table S1).

Sub-studies

Alongside the main study, four additional 'opt in' substudies allowed deeper phenotyping of participants. Substudies included consenting participants to approach them with opportunities for future research/cross-linkage with other studies (substudy 1); biobanking an additional large volume blood sample at baseline visit (substudy 2); retinal imaging with optical coherence tomography at baseline and twelve-month follow-up visit (substudy 3); and additional advanced magnetic resonance (MR) imaging sequences (substudy 4, baseline n = 78, follow up n = 74, complete pairs n = 67). A detailed description of the MR protocol and substudy is now published[24].

Clinical observations

Patient-reported and assessor-measured clinical observations were collected at each study visit. Source data from clinical assessments at local sites was captured using a web-based electronic case report form (eCRF). Both participants and study staff entered data directly. Clinical data were entered by participants via questionnaires. There was a high level of engagement with these questionnaires and assessments. Data completeness was >99% across all clinical measured and reported variables at both the baseline and year 1 visits.

Questionnaires included the multiple sclerosis impact scale (MSIS-29)[25], NICE domain activity & impairment, CDC Health Related Quality of Life (HRQOL-4)[26], Patient Determined Disease Steps

For peer review only

(PDDS)[27], Fatigue Severity Scale (FSS)[28], Generalized Anxiety Disorder Assessment (GAD-7)[29], depression assessment scoring (PHQ-9)[30], Baecke Habitual Physical activity[31], cognitive, leisure, social and lifestyle questionnaires. Clinical measures included the Expanded Disability Severity Score (EDSS)[32], the components of the Multiple Sclerosis Functional Composite score (MSFC)[33], mean arterial blood pressure (BP) and Body Mass Index (BMI).

[Table 2 approximately here]

Patient and public involvement

A focus group comprising of invited study participants meets regularly with the research team and has been involved in setting study priorities and the design of all waves. Written information is regularly sent to study participants with research updates, and participants are invited to join a voluntary network where they can be kept up to date with research progress if they wish to be. Principles of research transparency with study participants and shared priority setting for research agenda has been incorporated into the design of the study. Study participants will be invited to in person research update presentations, these are planned to recommence in the near future having been delayed due to COVID-19 restrictions and concerns about infection control during indoor gatherings.

Findings to date

At the time of writing, 392 of 440 participants (89.1%) had completed the return visit at one year and the first twenty patients have returned for follow up at year five with this third study wave now under way. At the end of the year one wave, six patients had withdrawn consent (1.4%), 23 (5.2%) were lost to follow up but will be invited to participate in subsequent visits. 19 (4.3%) participants had their return visit at one year prevented by the COVID-19 pandemic (visits due in March or April 2020). These participants will be invited for future follow up.

We present some illustrative results for the purpose of introducing the cohort and the data available to the research community.

Clinical observations

On some clinical measures participants improve on average over the first year (Table 2). This is not unexpected as diagnosis often coincides with a clinical event/relapse and we expect regression toward the mean over the course of the first year which may be amplified by effective treatment for some participants. Many of these clinical measures and questionnaires capture overlapping phenomena and are correlated. This provides both opportunities and challenges for asking causal questions of longitudinal repeated measures (see Fig. S3).

Lifestyle and social factors

[Figure 3 approximately here]

Lifestyle and social factors are known to influence MS disease course[34]. While some of these factors have been identified, there is much non-heritable variability that remains unexplained[35,36]. Amongst the strongest known environmental factor is smoking[37,38]. There is strong evidence that smoking both modifies the risk of MS incidence and affects the rate of disability progression. Smoking also interacts

For peer review only

statistically with disease risk loci[39]. In this cohort, 14.7% of participants were current smokers at the baseline visit and more than half (50.7%) declared themselves to be "ever smokers". By year one, there was a modest (1.7%) reduction in participants who currently smoke (13.0%). Figure 3A demonstrates that at baseline, the distribution of disability differs by smoking status.

As MS is associated with significant disability in working-age persons, it can impact employment and the quality of working life. Figure 3B demonstrates that at baseline the distribution of depression scores (PHQ-9) for persons who are employed and unemployment differs. The bimodal distribution of those who are unemployed suggests that a significant proportion of pwMS who are unemployed at baseline are at risk of depression.

Physical disability

[Figure 4 approximately here]

The distribution of measures of physical disability follows similar patterns across the two waves of the study cohort with little change over this early period (Fig. 4). This demonstrates the relatively insensitive nature of these measures to detect pathological disease activity early in disease course and over shorter study periods, at least at population level. However, FutureMS is sufficiently powered to allow meaningful comparison of sub-cohorts and of outlier individuals whose measured scores have worsened or improved in the first year. Figure 5 demonstrates an example of such an analysis: the group who have worsened over the first year are older and very few have low fatigue severity scores at baseline. Further hypothesis-driven analyses of these patterns may define clinically identifiable groups that explain some of the heterogeneity in disease course.

[Figure 5 approximately here]

Fatigue, mood and cognition

Fatigue has been described as the most disabling MS symptom by as many as 60% of patients in some studies[40]. The fatigue severity scale is important in the study assessment of MS disease impact. The biological basis of MS fatigue is poorly understood[41]. The distribution of participants suffering fatigue changes between the baseline and month 12 in our cohort. At follow up, the group does not appear to be monomodal which may reflect underlying biological heterogeneity or discrete sub-populations (Fig. 6A). Previous work has attempted to stratify fatigue into central or peripheral fatigue, and it may be that fatigue is a composite symptom with multiple pathogenic mechanisms[40,41]. Investigation of the natural history, burden and biology of fatigue will be a focus of study in the FutureMS cohort given the importance of this symptom to quality of life in pwMS.

[Figure 6 approximately here]

We observed a significant burden of depression in the study cohort, as measured by PHQ-9, highlighting the important contribution of mental health to MS burden early in the disease process (Fig. 6B). Median depression scores improved statistically significantly over the course of the first year from 7 to 4 ($p<10^{-12}$, two-tailed Wilcoxon signed-rank test).

SDMT and PASAT-3 tests revealed marked heterogeneity in the burden and trajectory of cognitive impairment between the study waves (Fig. 6C&D). Whilst the SDMT and PASAT-3 scores are significantly correlated (Spearman's rho 0.447 at baseline and 0.487 at follow up, both $p<10^{-15}$), some participants described at study visit that the PASAT-3 instrument was challenging or stressful. Those

who fail the trial run are recorded as zero contributing to a distribution of scores that was non-Gaussian. Cognitive scores at baseline and follow up correlated statistically significantly ($p<10^{-15}$) for both tests: SDMT (rho = 0.8) and PASAT-3 (rho = 0.76).

Adjusted measures of MS severity

[Figure 7 approximately here]

The multiple sclerosis severity score (MSSS) and age-related multiple sclerosis severity score (ARMSS) have been validated on large independent cohorts to attempt to standardise physical MS-related disability for disease duration[42,43]. The MSSS does this by normalising the EDSS for patient-reported disease duration. However, factors that influence recall of disease duration and are associated with EDSS may confound this measure. ARMSS normalises EDSS for patient age which is correlated with disease duration imperfectly but is not susceptible to recall biases. These measures, ARMSS and MSSS, correlate strongly and statistically significantly (all comparisons $p<10^{-15}$) in both study waves: rho = 0.69 at baseline and rho = 0.71 at year one (Fig. 7). The patient determined disease steps is a patient-reported outcome of disease severity validated for use in MS[27] and figure 7 demonstrates that there is an imperfect tendency for agreement with the objective measures. Further exploration is intended to explore the characteristics of participants whose reported disease severity and measured disability severity is discordant.

MR imaging

MR brain image protocols and processing have been described in detail elsewhere[24] , but in brief, participants from all centres were invited to undergo a standard protocol of structural 3T MRI sequences, including T1-weighted, T2-weighted and fluid attenuated inversion recovery (FLAIR) images. The study was powered to detect changes in brain imaging outcomes - not necessarily changes in clinical measures - at year one, as MR brain imaging measures have higher sensitivity over short time frames compared to clinical measures. The primary endpoint was new and/or enlarging T2 hyperintense lesions, as qualitatively (visually) assessed by expert neuroradiologists using brain imaging software (Table 2). The secondary endpoint was automated measurement of global brain volume change. In addition to the standard structural sequences, participants in Edinburgh were invited to undergo an advanced MR imaging protocol, comprising diffusion MRI (dMRI) and magnetisation transfer imaging (MTI). These measures allow for quantitative assessment of brain microstructure and therefore provide the opportunity to study brain myelin and axonal damage, which are prominent features of MS. dMRI and MTI metrics were used as exploratory endpoints of microstructural change in MS.

Optical coherence tomography

Participants of FutureMS were offered the opportunity to enrol in a sub-study of retinal imaging and optical coherence tomography. Proof of concept has been established in MS for the utility of retinal imaging with optical coherence tomography (OCT) to measure thinning of the retinal nerve fibre layer (RNFL), inner nuclear layer (INL), and the ganglion cell and inner plexiform layer (GCIP), all of which have been shown to correlate with clinical activity and disability[44].

Laboratory investigations

Blood sampling was performed at the baseline visit for routine laboratory testing, genetic testing, cell subsets, and biobanking for future studies. 'Routine' analysis included eGFR, HbA1c, CRP, vitamin D, albumin, cholesterol, high-density lipoprotein (HDL), low-density lipoprotein (LDL), very-low density

For peer review only

lipoprotein (VLDL), haemoglobin concentration (Hb), white cell count (WCC), and platelet count. All clinical assessments and procedures (e.g. blood draw) were performed in a standard sequence by the assessing neurologist or clinical research nurse. All samples were transported to laboratories for analysis immediately after venepuncture. Routine laboratory predictors were analysed in local NHS labs using their standard local protocols (Fig S4.).

Blood was collected at baseline for peripheral blood mononuclear cell (PBMC) isolation and deoxyribonucleic acid (DNA) extraction. DNA was extracted from 9ml Ethylenediaminetetraacetic acid (EDTA) whole blood using Nucleon BACC3 kit. DNA samples were re-suspended in 1ml TE buffer pH 7.5 (10mM Tris-Cl pH 7.5, 1mM EDTA pH 8.0). PBMCs were isolated from Lithium Heparin blood at each hub and samples shipped to the Edinburgh Clinical Research Facility (CRF) Genetics Core Laboratory for storage.

Immune cell subsets were isolated by fluorescence activated cell sorting (FACS) of PBMCs. Samples and sorted cell populations were kept on ice at all times. Prior to sorting on the BD FACSAriaII SORP cell sorter, the instrument was set-up using the internal Cytometer Set-Up and Tracking (CS&T) system, the drop delay was set to >99.9% with Accudrop beads to ensure sort quality. The Aria was set up with the 85um nozzle and 45psi pressure. Single stained controls were analysed with every run before a sort and compensation adjusted if necessary. The sample chamber and collection tube holder were cooled to 4°C. Collection tubes were pre-coated with 500uL cold medium. 5uL of 7-AAD were added to the cell sample 5 minutes before sorting, and the samples were filtered through 35um nylon mesh cell-strainers to avoid sample clumping. Gates were set on FSC-H and FSC-A to determine single cells, SSC-A and FSCA to exclude debris and 7-AAD negative population to exclude dead cells. The populations sorted were CD3+ CD4+ T-cells, CD3+ CD8+ T-cells, CD14+ Monocytes and CD19+ B-cells. The cells were run with a flowrate of 6.000 – 8.000 events per second. The maximum number of cells sorted per population was set to 1.5 x 10^{6} for the larger populations, and as many as possible for the smaller populations. Upon completion of the sort, a different sorted population from each sample was reanalysed on the instrument to evaluate the post-sort purity of the fractions across the samples. The number of cells sorted were recorded and sorted populations passed for RNA extraction (Fig. S5).

The fluorescent channels used were: 7-AAD excitation laser 488nm, 685/35nm BP filter, CD3-APC excitation laser 640nm, 670/14nm BP filter, CD14-FITC excitation laser 488nm, 525/50nm BP filter, CD19-BV excitation laser 405nm, 450/50nm BP filter, CD4-PE excitation laser 561nm, 582/15nm BP filter and CD8-BUV excitation laser 355nm, 450/50nm BP filter.

Bulk RNA-sequencing has been performed on these cell subsets and aliquots of cells also frozen for future analyses. RNA was extracted from sorted cell fractions using Qiagen miRNeasy. Yield and RIN were measured by Qubit RNA HS and Agilent Fragment Analyser. 1ng of each total RNA sample was fragmented and first-strand cDNA was generated using the SMARTer Stranded Total RNA-Seq Kit - Pico Input Mammalian kitIllumina-compatible adapters and indexes were added via 5 cycles of PCR. AMPure XP beads (Beckman Coulter) were used to purify the cDNA library followed by ribosomal RNA depletion using ZapR and R-Probes. Uncleaved fragments were enriched by 15 cycles of PCR before a final library purification using AMPure XP beads and sequencing on an Illumina NovaSeq.

Summary results for cell proportions suggested significant inter-individual variability in the proportion of viable cells that were B cells, monocytes, or T cells (Table S2) despite the lack of DMTs in this cohort. Future work is intended to explore whether these are meaningful parameters predictive of current or future neuroinflammatory activity or treatment response.

Additional (fluid) biomarkers of neuroinflammation have been analysed at baseline and will be described in detail elsewhere. These include neurofilament light chains (NfL), GFAP, Tau, UCH-L1 measured using digital ELISA/Single Molecule Array (SIMOA). CSF biomarkers have also been analysed for a subset of study participants.

For peer review only

SNP genotyping

Although environmental factors (particularly EBV infection, smoking, obesity during adolescence) are known to make important contributions to MS risk[45], there is an important heritable component evidenced from correlation between relatives[46]. The strongest known contribution to this heritability is for the HLA region of chromosome 6[47]. Despite the remarkable allelic heterogeneity observed at this region, HLA DRB1*15:01 (marked by rs3135388 and SNPs in high linkage disequilibrium with this allele) is known to dominate the contribution to this risk[48]. Additionally, over 200 non-HLA loci are associated with disease risk[49]. Less is known about genetic contributions to the variance of disease course. Figure S6 demonstrates the high confidence in calling SNP genotypes linked to HLADRB1*15:01 in FutureMS and table 3 demonstrates the expected finding of significant overrepresentation of the HLA DRB1*15:01 risk loci.

[Table 3 approximately here] [Figure 8 approximately here]

However, as shown in Figure 8, despite dominating the contribution to MS risk, the HLA-DRB1*15:01 genotype does not explain much, if any, of the baseline heterogeneity in the age at diagnosis, measured disability severity, or participant reported disease impact in the FutureMS cohort. This underscores that risk genes (e.g. HLA-DRB1*15:01) may not necessarily intersect the gene set that influences disease course. After quality control, 713,026 SNPs are available for genome-wide analyses in the FutureMS cohort from successful genotyping of 427/428 cases and 100/100 controls for whom PBMCs were available for DNA extraction (see Table S3). Analysis of genetic stratification within the cohort demonstrated little evidence of population stratification by study site and broad overlap between cases and controls with a small number of outliers in the control population relative to the cohort population (Fig. S7). These outliers are likely to reflect representation of persons with recent non-Scottish ancestry in the control population who will be relatively less likely to appear as cases, consistent with the high incidence of MS in Scotland and the findings of migration studies[50,51]. Investigation of the genetic and gene-environment interactions that explain heterogeneity and personal disease trajectories is a focus of ongoing analyses.

Genotyping methods

Extracted DNA was normalised to 50ng/ul after quantification using Qubit. Samples were genotyped using Infinium HTS chemistry and Infinium Global Screening Array-24 kit. Arrays were scanned on an Illumina iScan system and genotypes were called using GenomeStudio v2.0.3. Genotype calls using GenCall (v6.3.0) with a cut-off specified at 0.15, were then manually reviewed within Genome Studio, using a rigorous multi-step appraisal of cluster fit based on cluster separation score, call frequency, heterozygous excess, heterozygous mean normalised intensity and theta, and minor allele frequency. This was in line with manufacturer published instructions. Further QC and analysis of genetic stratification was performed using PLINK v1.9 and v2.0 and R v3.5.2.

DMT usage

At first follow up visit participants were asked to detail their DMT usage since the previous study wave. It is anticipated that these data will become increasingly more complex in subsequent waves and, where consent is granted, DMT medication histories will be confirmed using multiple data sources for each participant including electronic prescription records, and by contacting the patient's treating neurology team and general practice. Table 4 summarises the DMT usage in the first year of the cohort as reported by the participants. These data reveal that 65.6% of participants have been prescribed any DMT in the first year. The most common early treatment used in Scotland is Dimethyl Fumarate with 131 participants (32.9%) prescribed this treatment as a first line therapy. The maximum number of DMTs

For peer review only

in the cohort at year one was two, with 23 participants (5.5%) having been prescribed two DMTs within the 12 months of follow up. Alemtuzumab was the most common high efficacy DMT used.

[Table 4 approximately here]

Data Management

Participants were identified with a unique non-identifiable study number, which was used to label all paperwork, biological samples and imaging obtained throughout the duration of the study. Questionnaires and clinical data were entered in real time to a FutureMS electronic case report form via an online platform. Data were managed in accordance with the Data Protection Act (DPA 1998), NHS Scotland, and University of Edinburgh policies.

Missing Data Handling

Most (395/440) participants in the study have complete (100%) baseline records comprising 189 variables in the core clinical dataset. Similar completeness of data was observed for month 12: >99% across all clinical measured and reported variables at both baseline and month 12 follow up. Where missing, source data were carefully inspected and the likely cause for missingness was appraised by a multidisciplinary study team (study nurses and statistically-trained clinicians). Where data were missing at random, multiple imputation with chained equations (MICE) by predictive mean matching (PMM) was used to impute baseline measures from across the cohort. Data missing not at random were left missing where appropriate (e.g. for smoking status) or substituted where appropriate (e.g. when missing due to disability, a low z-score was substituted for the missing a timed 25ft walk test to reflect this disability).

For data missing at month 12, a similar approach was employed, with the participant's data at the baseline wave also incorporated as additional multivariable predictors alongside cohort performance at year one in the PMM approach. It is intended that this approach combining prediction based on prior individual performance, and cohort performance adjusting for correlated characteristics, will minimise bias and will be employed in future waves for data missing at random. This approach will also be employed to handle missing data for those individuals who are lost to follow up. However, the exact method of handling missing data from loss to follow up in future waves will be determined by the analysis questions and it is anticipated that sensitivity analyses using other methods (e.g. complete cases and inverse probability weights) will be employed and reported to determine the sensitivity of results to the analysis methods if a significant proportion of the cohort are eventually lost to follow-up.

Data Retention

Data acquired in FutureMS may be of potential long-term scientific value. All data collected will be retained for a minimum of 30 years after study completion. Collected data will also be retained after the withdrawal of participants for any reason including loss of capacity. No identifiable data will be shared with third parties, but proposals for collaborative, ethically-approved research projects utilising these data will be welcomed.

Statistical analyses

Multivariable mixed effects regression models, latent class/transition models, and network-based analyses are planned for subsequent investigation of relationships between variables and will be

For peer review only

explained in detail elsewhere. Prior to receiving access to study data, all investigators proposing analyses will be required to formally pre-specify statistical analysis plans and to justify hypothesis testing with appropriate pre-declaration of clinically meaningful effect sizes and power calculations where appropriate. Research proposals will be appraised by a committee with suitable multidisciplinary expertise for the proposed project: this may include clinical, statistical, bioinformatic, genetic, immunology and other subject matter expertise. All research proposals will be considered on their merits ensuring that appropriate prior justification of hypotheses exists to reduce the risk of spurious research findings arising from indiscriminate multiple testing.

The large number of recorded variables available to FutureMS researchers will allow multivariable adjustment for important confounders. However, proposed research projects will be expected to explicitly justify the statistical approach to confounding in their proposed analysis and to document evidence and a rational approach based on subject matter prior knowledge and the published literature. Statistical adjustment for confounding and non-adjustment for other relevant variables (e.g. mediators and colliders) in analyses will be expected to be pre-specified and justified to avoid producing misleading results, or the introduction bias or overfitting.

Ethics

The study is conducted in conformity with the declaration of Helsinki, ICH guidelines for Good Clinical Practice (CPMP/ICH/135/95), and with ethical approval granted from the National Health Service (NHS) South East Scotland Research Ethics Committee (02) (Reference: 15/SS/0233) and approval granted from individual NHS Board Research and Development departments (IRAS Project ID: 169955). Control samples were collected under approval granted from the NHS East of Scotland Research Ethics committee (REC01) as part of the Scottish Regenerative Neurology Tissue Bank Project (Reference: 15/ES/0094).

Discussion

We have designed and recruited a large cohort of persons with RRMS across Scotland. The prospective nature of FutureMS enables longitudinal assessment of clinical, imaging, genomic and fluid biomarkers in all participants prior to and during disease modifying treatments. As the number of available treatment options increases, so too must our understanding of the heterogeneity of disease course for persons living with RRMS. Substantial effort has been made to ensure that the study has recruited a geographically, socioeconomically, and clinically representative national cohort. The results presented here give us confidence that this has been achieved such that findings from this study may be generalised to clinical practice.

Scotland has long been known to have a high incidence of multiple sclerosis[52]. The reasons for this remain unknown despite long-running speculation[17,53–65]. The Scottish northern isles for many decades have been recognised as particularly burdened[66]. Our early exploration of genetic results confirms expected findings of an excess of HLA-DRB1*15:01 (OR 3.90, 95% CI: 2.50 – 6.34) in the Scottish MS population. This provides a useful prevalence benchmark by which this (and other) genetic loci can be assessed and compared to other MS populations. The risk allele frequency (RAF) in the FutureMS cases of 0.34 is high by previously published standards[67–70], but not extremely so with numerous published case control studies reporting higher frequencies of this gene[71]. The frequency in controls (0.12), is similarly high, but not excessively so. Taken together, the excess frequency in cases underscores the highly probable importance of this gene's contribution to MS risk in Scotland (as elsewhere) but leaves room for other genetic or environmental factors to explain why Scotland has a particularly high incidence of MS. Substantial further exploration is required and is intended to address this issue.

For peer review only

Recent and historical studies have noted regional variation in the distribution of the burden of MS across Scotland[17,53,64,72], consistent with findings in many other countries where regional analyses have been performed[73–77]. A strength of this study is that in being geographically representative of the national population it may be well positioned to investigate genetic and environmental hypotheses for this spatial heterogeneity in disease burden.

In comparison to other diseases with strong environmental risk factors, MS seems to be less associated with indices of socioeconomic deprivation than might be expected. This has been recognised in multiple epidemiological studies in Scotland[52,78,79] and has not been explained to date. In fact, it is intriguingly paradoxical given established MS environmental risk factors (e.g. Vitamin D deficiency, obesity in adolescence and smoking) are strongly associated with deprivation in Scotland[80–83].

The exploratory analyses presented here demonstrate that our cohort can be considered nationally representative. However, we suggest caution generalising any findings from this population to individuals who fall outside of the remit of our study. For example, to those pwMS who experience such aggressive disease at onset that DMT is initiated emergently (as these individuals would not have been eligible for recruitment), or to those diagnosed at extremes of the age distribution (particularly <18). Similarly, caution may be necessary if attempting to generalise to populations with more heterogenous recent ancestry and to those whose initial presentation is with progressive disease.

Multiple sclerosis is a clinically heterogenous disease, presenting with variable symptoms affecting different parts of the central nervous system, which may be interspersed by prolonged periods without overt disease[1]. This heterogeneity can make the diagnosis a challenging one and delayed diagnosis is common. Variability between patients in care-seeking behaviours and between clinicians can compound this heterogeneity. Although we used six months as a proxy for 'newly diagnosed' this does not necessarily equate to 'early' disease from a pathophysiological view, and this is an important limitation of our study. This is shown by the time taken from first symptom to diagnosis ranging from a single day to 33.5 years in the FutureMS cohort. It is perhaps inevitable, therefore, that in an inception cohort like FutureMS, biological markers at baseline will be variably reflective of their true levels at disease onset. However, a strength of this study is that participants were enrolled as early as possible after diagnosis. Whilst date of disease diagnosis will not be equivalent to date of disease onset, it is a best practical compromise possible for a study of this size.

Our early exploration of the association with disability severity and demographic and lifestyle factors highlighted an obvious difference, observable at baseline, in measures of physical disability between current and non-smokers. Importantly, the proportion of current smokers changed by a very small proportion over the first year of the study, despite strong evidence of the risk of smoking worsening disease activity[84]. This brings into focus the need to counsel all persons newly-diagnosed with MS who smoke, as early as possible, and to provide information on the benefits of cessation and the MS-specific harm of smoking, including passive smoking. It is likely that for some pwMS, particularly those for whom smoking is compounded with genetic predisposition, the benefit of smoking cessation may be very substantial.

Fortunately, depression as measured by PHQ-9 is one of the clinical measures that improves most in the first year following diagnosis. However, we noted a high burden of depression by this measure at baseline, and particularly in persons diagnosed with MS who are also unemployed. Numerous possible explanations for the relationship between depression and employment are plausible, and further work will be necessary and is intended to delineate the causal structure of this relationship to guide effective interventions. These findings underscore the importance of considering the burden of mental health early in MS. It is reassuring that these scores improve on average in the early phase of the condition, and this may be of reassurance to some patients, and may encourage mental health treatment where mood is not improving.

In conclusion, we anticipate that long-term follow up of the FutureMS cohort will lead to the development of clinically useful tools for predicting future disability in patients with multiple sclerosis.

For peer review only

Statements

Contributorship

P.K.A.K. performed data cleaning (assisted by C.W.,) analysed the data and wrote the first draft of the manuscript. P.K.A.K., S.J.M., J.C., R.M., E.N.Y., contributed figures and/or analyses to the paper. P.K.A.K., S.J.M., Y.C., C.W., A.S., P.C., S.C., conducted patient visits and collected primary data. K.H., S.T., E.F., L.M., analysed laboratory data and performed genetics experiments. R.M., E.N.Y., Y.C., A.D.W., analysed radiological/imaging data. P.K.A.K., S.J.M., R.M., E.N.Y., C.W., A.S., A.H., P.F., D.H., A.D.W., P.C., S.C., planned analyses and managed data collection. P.C., M.McL., J.O.R., F.J.C-A., N.J.J. MacD., S.C., were responsible for study sites. S.E.B., A.H., L.M., planned and conducted genetics and transcriptomics experiments. P.C., A.W., S.C., conceived the idea for cohort, designed, secured funding, approvals for the study. All authors contributed to revising drafts of the initial manuscript and satisfy ICMJE 2018 criteria for authorship.

Conflicts

The authors declare no conflicts of interest.

Funding

FutureMS has been funded by Precision Medicine Scotland Innovation Centre (PMS_IC: R44346 & R44800), the Rowling Clinic, and Biogen Idec. Ltd. Insurance provided by the Co-Sponsors: NHS Lothian and the University of Edinburgh. The funders had no role in the design of the study, collection of the data, analysis of the data, or the decision to publish.

Collaboration and data sharing

Data (and/or samples) are available after approval of a research proposal via an established subcommittee. The study team welcomes enquiries for collaboration or external research proposals using the cohort data and/or samples.

REFERENCES

1. Compston A, McDonald I, Noseworthy J, et al. *McAlpine's Multiple Sclerosis*. 4th ed. Churchill Livingstone Elsevier; 2006. doi:10.1016/B978-0-443-07271-0.X5001-0
2. Confavreux C, Vukusic S. Natural history of multiple sclerosis: a unifying concept. *Brain*. 2006;129(3):606-616. doi:10.1093/brain/awl007
3. Benedikz J, Stefánsson M, Guomundsson J, et al. The natural history of untreated multiple sclerosis in Iceland. A total population-based 50 year prospective study. *Clin Neurol Neurosurg*. 2002;104(3):208-210. http://www.ncbi.nlm.nih.gov/pubmed/12127656. Accessed March 2, 2017.
4. Kappos L, Li D, Calabresi PA, et al. Ocrelizumab in relapsing-remitting multiple sclerosis: a phase 2, randomised, placebo-controlled, multicentre trial. *Lancet*. 2011;378(9805):1779-1787. doi:10.1016/S0140-6736(11)61649-8
5. Hauser SL, Waubant E, Arnold DL, et al. B-Cell Depletion with Rituximab in Relapsing–Remitting Multiple Sclerosis. *N Engl J Med*. 2008;358(7):676-688. doi:10.1056/NEJMoa0706383
6. Comi G, Kappos L, Selmaj KW, et al. Safety and efficacy of ozanimod versus interferon beta-1a in relapsing multiple sclerosis (SUNBEAM): a multicentre, randomised, minimum 12-month, phase 3 trial. *Lancet Neurol*. 2019;18(11):1009-1020. doi:10.1016/S1474-4422(19)30239-X

For peer review only

7. Polman CH, O'Connor PW, Havrdova E, et al. A Randomized, Placebo-Controlled Trial of Natalizumab for Relapsing Multiple Sclerosis. *N Engl J Med*. 2006;354(9):899-910. doi:10.1056/NEJMOA044397/SUPPL_FILE/NEJM_POLMAN_899SA1.PDF

8. Khan OA, Tselis AC, Kamholz JA, Garbern JY, Lewis RA, Lisak RP. A prospective, open-label treatment trial to compare the effect of IFN beta-1a (Avonex), IFNbeta-1b (Betaseron), and glatiramer acetate (Copaxone) on the relapse rate in relapsing-remitting multiple sclerosis. *Eur J Neurol*. 2001;8(2):141-148. doi:10.1046/J.1468-1331.2001.00189.X

9. Giovannoni G, Comi G, Cook S, et al. A Placebo-Controlled Trial of Oral Cladribine for Relapsing Multiple Sclerosis. *N Engl J Med*. 2010;362(5):416-426. doi:10.1056/NEJMOA0902533/SUPPL_FILE/NEJM_GIOVANNONI_416SA1.PDF

10. Coles AJ, Twyman CL, Arnold DL, et al. Alemtuzumab for patients with relapsing multiple sclerosis after disease-modifying therapy: a randomised controlled phase 3 trial. *Lancet (London, England)*. 2012;380(9856):1829-1839. doi:10.1016/S0140-6736(12)61768-1

11. Cohen JA, Coles AJ, Arnold DL, et al. Alemtuzumab versus interferon beta 1a as first-line treatment for patients with relapsing-remitting multiple sclerosis: a randomised controlled phase 3 trial. *Lancet (London, England)*. 2012;380(9856):1819-1828. doi:10.1016/S0140-6736(12)61769-3

12. Renoux C. Natural history of multiple sclerosis: long-term prognostic factors. *Neurol Clin*. 2011;29(2):293-308. doi:10.1016/J.NCL.2011.01.006

13. Confavreux C, Vukusic S, Adeleine P. Early clinical predictors and progression of irreversible disability in multiple sclerosis: an amnesic process. *Brain*. 2003;126(Pt 4):770-782. doi:10.1093/BRAIN/AWG081

14. Tintore M, Rovira À, Río J, et al. Defining high, medium and low impact prognostic factors for developing multiple sclerosis. *Brain*. 2015;138(Pt 7):1863-1874. doi:10.1093/BRAIN/AWV105

15. Brown FS, Glasmacher SA, Kearns PKA, et al. Systematic review of prediction models in relapsing remitting multiple sclerosis. *PLoS One*. 2020;15(5). doi:10.1371/JOURNAL.PONE.0233575

16. Hauser SL, Cree BAC. Treatment of Multiple Sclerosis: A Review. *Am J Med*. 2020;133(12):1380-1390.e2. doi:10.1016/J.AMJMED.2020.05.049

17. Kearns PKA, Paton M, O'Neill M, et al. *Regional Variation in the Incidence Rate and Sex Ratio of Multiple Sclerosis in Scotland 2010–2017: Findings from the Scottish Multiple Sclerosis Register*.; 2019. doi:10.1007/s00415-019-09413-x

18. Thompson AJ, Banwell BL, Barkhof F, et al. Diagnosis of multiple sclerosis: 2017 revisions of the McDonald criteria. *Lancet Neurol*. 2018;17(2):162-173. doi:10.1016/S1474-4422(17)30470-2

19. Polman CH, Reingold SC, Banwell B, et al. Diagnostic criteria for multiple sclerosis: 2010 revisions to the McDonald criteria. *Ann Neurol*. 2011;69(2):292-302. doi:10.1002/ANA.22366

20. Kremenchutzky M, Rice GPA, Baskerville J, Wingerchuk DM, Ebers GC. The natural history of multiple sclerosis: a geographically based study 9: observations on the progressive phase of the disease. *Brain*. 2006;129(Pt 3):584-594. doi:10.1093/BRAIN/AWH721

21. Confavreux C, Vukusic S. Natural history of multiple sclerosis: a unifying concept. *Brain*. 2006;129(Pt 3):606-616. doi:10.1093/BRAIN/AWL007

22. Tutuncu M, Tang J, Zeid NA, et al. Onset of progressive phase is an age-dependent clinical milestone in multiple sclerosis. *Mult Scler J*. 2013;19(2):188-198. doi:10.1177/1352458512451510

23. Tremlett H, Zhao Y, Devonshire V. Natural history of secondary-progressive multiple sclerosis. *Mult Scler*. 2008;14(3):314-324. doi:10.1177/1352458507084264

24. Meijboom R, Wiseman SJ, York EN, et al. Rationale and design of the brain magnetic resonance

For peer review only

imaging protocol for FutureMS: a longitudinal multi-centre study of newly diagnosed patients with relapsing-remitting multiple sclerosis in Scotland. *Wellcome Open Res 2022 794*. 2022;7:94. doi:10.12688/wellcomeopenres.17731.1

25. Hobart J, Lamping D, Fitzpatrick R, Riazi A, Thompson A. The multiple sclerosis impact scale (MSIS-29) a new patient-based outcome measure. *Brain*. 2001;124(5):962-973. doi:10.1093/brain/124.5.962

26. Richardson LC, Wingo PA, Zack MM, Zahran HS, King JB. Health-related quality of life in cancer survivors between ages 20 and 64 years: Population-based estimates from the behavioral risk factor surveillance system. *Cancer*. 2008;112(6):1380-1389. doi:10.1002/cncr.23291

27. Learmonth YC, Motl RW, Sandroff BM, Pula JH, Cadavid D. Validation of patient determined disease steps (PDDS) scale scores in persons with multiple sclerosis. *BMC Neurol*. 2013;13. doi:10.1186/1471-2377-13-37

28. Krupp LB, Larocca NG, Muir Nash J, Steinberg AD. The fatigue severity scale: Application to patients with multiple sclerosis and systemic lupus erythematosus. *Arch Neurol*. 1989;46(10):1121-1123. doi:10.1001/archneur.1989.00520460115022

29. Spitzer RL, Kroenke K, Williams JBW, Löwe B. A brief measure for assessing generalized anxiety disorder: The GAD-7. *Arch Intern Med*. 2006;166(10):1092-1097. doi:10.1001/archinte.166.10.1092

30. Kroenke K, Spitzer RL, Williams JBW. The PHQ-9: Validity of a brief depression severity measure. *J Gen Intern Med*. 2001;16(9):606-613. doi:10.1046/j.1525-1497.2001.016009606.x

31. Baecke JAH, Burema J, Frijters JER. A short questionnaire for the measurement of habitual physical activity in epidemiological studies. *Am J Clin Nutr*. 1982;36(5):936-942. doi:10.1093/ajcn/36.5.936

32. Cohen RA, Kessler HR, Fischer M. The extended disability status scale (EDSS) as a predictor of impairments of functional activities of daily living in multiple sclerosis. *J Neurol Sci*. 1993;115(2):132-135. doi:10.1016/0022-510X(93)90215-K

33. Polman CH, Rudick RA. The multiple sclerosis functional composite a clinically meaningful measure of disability. *Neurology*. 2010;74(17 SUPPL. 3). doi:10.1212/WNL.0b013e3181dbb571

34. Hagan KA, Munger KL, Ascherio A, Grodstein F. Epidemiology of Major Neurodegenerative Diseases in Women: Contribution of the Nurses' Health Study. *Am J Public Health*. 2016;106(9). doi:10.2105/AJPH.2016.303324

35. Fagnani C, Neale MC, Nisticò L, et al. Twin studies in multiple sclerosis: A meta-estimation of heritability and environmentality. *Mult Scler*. 2015;21(11):1404-1413. doi:10.1177/1352458514564492

36. International Multiple Sclerosis Genetics Consortium TIMSGC, Wellcome Trust Case Control Consortium 2 WTCCC 2, Sawcer S, et al. Genetic risk and a primary role for cell-mediated immune mechanisms in multiple sclerosis. *Nature*. 2011;476(7359):214-219. doi:10.1038/nature10251

37. Belbasis L, Bellou V, Evangelou E, Ioannidis JPA, Tzoulaki I. Environmental risk factors and multiple sclerosis: an umbrella review of systematic reviews and meta-analyses. *Lancet Neurol*. 2015;14(3):263-273. doi:10.1016/S1474-4422(14)70267-4

38. O 'gorman C, Lucas R, Taylor B. Environmental Risk Factors for Multiple Sclerosis: A Review with a Focus on Molecular Mechanisms. *Int J Mol Sci*. 2012;13:11718-11752. doi:10.3390/ijms130911718

39. Hedström AK, Katsoulis M, Hössjer O, et al. The interaction between smoking and HLA genes in multiple sclerosis: replication and refinement. *Eur J Epidemiol*. 2017;32(10):909-919. doi:10.1007/s10654-017-0250-2

For peer review only

40. Newland P, Starkweather A, Sorenson M. Central fatigue in multiple sclerosis: a review of the literature. *J Spinal Cord Med*. 2016;39(4):386-399. doi:10.1080/10790268.2016.1168587

41. Manjaly ZM, Harrison NA, Critchley HD, et al. Pathophysiological and cognitive mechanisms of fatigue in multiple sclerosis. *J Neurol Neurosurg Psychiatry*. 2019;90(6):642-651. doi:10.1136/JNNP-2018-320050

42. Manouchehrinia A, Westerlind H, Kingwell E, et al. Age Related Multiple Sclerosis Severity Score: Disability ranked by age. *Mult Scler*. 2017;23(14):1938-1946. doi:10.1177/1352458517690618

43. Weideman AM, Barbour C, Tapia-Maltos MA, et al. New Multiple Sclerosis Disease Severity Scale Predicts Future Accumulation of Disability. *Front Neurol*. 2017;8:598. doi:10.3389/fneur.2017.00598

44. Petzold A, de Boer JF, Schippling S, et al. Optical coherence tomography in multiple sclerosis: A systematic review and meta-analysis. *Lancet Neurol*. 2010;9(9):921-932. doi:10.1016/S1474-4422(10)70168-X

45. Belbasis L, Bellou V, Evangelou E, Ioannidis JPA, Tzoulaki I. Environmental risk factors and multiple sclerosis: an umbrella review of systematic reviews and meta-analyses. *Lancet Neurol*. 2015;14(3):263-273. doi:10.1016/S1474-4422(14)70267-4

46. Dyment DA, Yee IML, Ebers GC, Sadovnick AD, Canadian Collaborative Study Group. Multiple sclerosis in stepsiblings: recurrence risk and ascertainment. *J Neurol Neurosurg Psychiatry*. 2006;77(2):258-259. doi:10.1136/jnnp.2005.063008

47. Sawcer S, Hellenthal G, Pirinen M, et al. Genetic risk and a primary role for cell-mediated immune mechanisms in multiple sclerosis. *Nature*. 2011;476(7359):214-219. doi:10.1038/nature10251

48. De Bakker PIW, McVean G, Sabeti PC, et al. A high-resolution HLA and SNP haplotype map for disease association studies in the extended human MHC. *Nat Genet*. 2006;38(10):1166-1172. doi:10.1038/ng1885

49. Baranzini SE, Oksenberg JR. The Genetics of Multiple Sclerosis: From 0 to 200 in 50 Years. *Trends Genet*. 2017;33(12):960-970. doi:10.1016/j.tig.2017.09.004

50. Cabre P, Signate A, Olindo S, et al. Role of return migration in the emergence of multiple sclerosis in the French West Indies. *Brain*. 2005;128(12):2899-2910. doi:10.1093/brain/awh624

51. Munk Nielsen N, Corn G, Frisch M, et al. Multiple sclerosis among first- and second-generation immigrants in Denmark: a population-based cohort study. *Brain*. 2019;142(6):1587-1597. doi:10.1093/brain/awz088

52. Bramwell B. The Relative Frequency of Disseminated Sclerosis in this country (Scotland and the North of England) and in America. *Rev Neurol Psychiatry R Coll Physicians Edinburgh*. 1903;1:12.

53. Dean G, Goodall J, Downie A. The prevalence of multiple sclerosis in the Outer Hebrides compared with north-east Scotland and the Orkney and Shetland Islands. *J Epidemiol Community Health*. 1981;35(2):110-113. http://www.ncbi.nlm.nih.gov/pubmed/7299334. Accessed January 2, 2019.

54. Cruz PMR, Matthews L, Boggild M, et al. Time-and region-specific season of birth effects in multiple sclerosis in the United Kingdom. *JAMA Neurol*. 2016;73(8):954-960. doi:10.1001/jamaneurol.2016.1463

55. Sutherland JM. Observations on the prevalence of multiple sclerosis in Northern Scotland. *Brain*. 1956;79(4):635-654. http://www.ncbi.nlm.nih.gov/pubmed/13396068. Accessed January 7, 2019.

56. Ebers G. Month of Birth and Multiple Sclerosis Risk in Scotland. *Eur Neurol*. 2010;63:41-42. doi:10.1159/000268164

57. Poser CM. The Dissemination of Multiple Sclerosis: A Viking Saga? A Hihstorical Essay.

For peer review only

https://eurekamag.com/pdf.php?pdf=009556512. Accessed July 11, 2017.

58. Donnan PT, Parratt J DE, Wilson S V, Forbes RB, O'Riordan JI, Swingler RJ. Multiple sclerosis in Tayside, Scotland: detection of clusters using a spatial scan statistic. *Mult Scler J*. 2005;11(4):403-408. doi:10.1191/1352458505ms1191oa

59. Taylor R, Illsley R, Poskanzer DC. *Multiple Sclerosis in the Orkney and Shetland Islands VI: The Effects of Migration and Social Structure*. Vol 34.; 1980. https://www.ncbi.nlm.nih.gov/pmc/articles/PMC1052090/pdf/jepicomh00261-0043.pdf. Accessed January 2, 2019.

60. Mackenzie IS, Morant S V., Bloomfield GA, MacDonald TM, O'Riordan J. Incidence and prevalence of multiple sclerosis in the UK 1990-2010: a descriptive study in the General Practice Research Database. *J Neurol Neurosurg Psychiatry*. 2014;85(1):76-84. doi:10.1136/jnnp-2013-305450

61. Phadke JG, Downie AW. Epidemiology of multiple sclerosis in the north-east (Grampian Region) of Scotland-an update. *J Epidemiol Community Health*. 1987;41:5-13. doi:10.1136/jech.41.1.5

62. Handel AE, Jarvis L, McLaughlin R, Fries A, Ebers GC, Ramagopalan S V. The epidemiology of multiple sclerosis in Scotland: inferences from hospital admissions. *PLoS One*. 2011;6(1):e14606. doi:10.1371/journal.pone.0014606

63. Visser EM, Wilde K, Wilson JF, Yong KK, Counsell CE. A new prevalence study of multiple sclerosis in Orkney, Shetland and Aberdeen city. *J Neurol Neurosurg Psychiatry*. 2012;83(7):719-724. doi:10.1136/jnnp-2011-301546

64. Shepherd DI, Downie AW. Prevalence Of Multiple Sclerosis In North-East Scotland. *BMJ*. 1978;2:314-316. https://www.jstor.org/stable/25428452?seq=1#metadata_info_tab_contents. Accessed February 6, 2021.

65. Rothwell PM, Charlton D. High incidence and prevalence of multiple sclerosis in south east Scotland: evidence of a genetic predisposition. *J Neurol Neurosurg Psychiatry*. 1998;64:730-735. https://www.ncbi.nlm.nih.gov/pmc/articles/PMC2170112/pdf/v064p00730.pdf. Accessed January 25, 2018.

66. Poskanzer DC, Prenney LB, Sheridan JL, Kondy JY. *Multiple Sclerosis in the Orkney and Shetland Islands I: Epidemiology, Clinical Factors, and Methodology*. Vol 34.; 1980. https://www.ncbi.nlm.nih.gov/pmc/articles/PMC1052085/pdf/jepicomh00261-0001.pdf. Accessed December 22, 2018.

67. International Multiple Sclerosis Genetics Consortium, Hafler DA, Compston A, et al. Risk Alleles for Multiple Sclerosis Identified by a Genomewide Study. *N Engl J Med*. 2007;357(9):851-862. doi:10.1056/NEJMoa073493

68. Alcina A, de Abad-Grau MM, Fedetz M, et al. Multiple sclerosis risk variant HLA-DRB1*1501 associates with high expression of DRB1 gene in different human populations. *PLoS One*. 2012;7(1). doi:10.1371/journal.pone.0029819

69. Benešová Y, Vašků A, Štourač P, Hladíková M, Fiala A, Bednařík J. Association of HLA-DRB1*1501 tagging rs3135388 gene polymorphism with multiple sclerosis. *J Neuroimmunol*. 2013;255(1-2):92-96. doi:10.1016/j.jneuroim.2012.10.014

70. George MF, Briggs FBS, Shao X, et al. Multiple sclerosis risk loci and disease severity in 7,125 individuals from 10 studies. *Neurol Genet*. 2016;2(4). doi:10.1212/NXG.0000000000000087

71. Schmidt H, Williamson D, Ashley-Koch A. Human Genome Epidemiology (HuGE) Review HLA-DR15 Haplotype and Multiple Sclerosis: A HuGE Review. doi:10.1093/aje/kwk118

72. Roberts DF, Roberts MJ, Poskanzer DC. Genetic analysis of multiple sclerosis in Shetland. *J*

For peer review only

Epidemiol Community Health. 1983;37(4):281-285. doi:10.1136/jech.37.4.281

73. Bihrmann K, Nielsen NM, Magyari M, Koch-Henriksen N, Nordsborg RB, Ersbøll AK. Small-scale geographical variation in multiple sclerosis: A case-control study using Danish register data 1971-2013. *Mult Scler Relat Disord*. 2018;23:40-45. doi:10.1016/j.msard.2018.04.021

74. Kurtzke JF. Multiple sclerosis in time and space--geographic clues to cause. *J Neurovirol*. 2000;6 Suppl 2:S134-40. http://www.ncbi.nlm.nih.gov/pubmed/10871801. Accessed October 2, 2017.

75. Wallin MT, Culpepper WJ, Coffman P, et al. The Gulf War era multiple sclerosis cohort: age and incidence rates by race, sex and service. *Brain*. 2012;135(6):1778-1785. doi:10.1093/brain/aws099

76. Van Der Mei IAF, Ponsonby AL, Blizzard L, Dwyer T. Regional variation in multiple sclerosis prevalence in Australia and its association with ambient ultraviolet radiation. *Neuroepidemiology*. 2001;20(3):168-174. doi:10.1159/000054783

77. Beck CA, Metz LM, Svenson LW, Patten SB. Regional variation of multiple sclerosis prevalence in Canada. *Mult Scler*. 2005;11(5):516-519. doi:10.1191/1352458505ms1192oa

78. Murray S, Bashir K, Penrice G, Womersley SJ. Epidemiology of multiple sclerosis in Glasgow. *Scott Med J*. 2004;49(3):100-104. doi:10.1177/003693300404900310

79. Kearns PKA, Paton M, O'Neill M, et al. Regional variation in the incidence rate and sex ratio of multiple sclerosis in Scotland 2010–2017: findings from the Scottish Multiple Sclerosis Register. *J Neurol*. 2019;266(10). doi:10.1007/s00415-019-09413-x

80. Ascherio A, Munger KL. Environmental risk factors for multiple sclerosis. Part II: Noninfectious factors. *Ann Neurol*. 2007;61(6):504-513. doi:10.1002/ana.21141

81. Tod E, Bromley C, Millard AD, Boyd A, Mackie P, McCartney G. Obesity in Scotland: a persistent inequality. *Int J Equity Health*. 2017;16(1):135. doi:10.1186/s12939-017-0599-6

82. NHS Health Scotland, ISD Scotland. *An Atlas of Tobacco Smoking In Scotland: A REPORT PRESENTING ESTIMATED SMOKING PREVALENCE AND SMOKING-ATTRIBUTABLE DEATHS WITHIN SCOTLAND*.; 2007. http://www.healthscotland.com/uploads/documents/7882-AnAtlasOfTobaccoSmokingInScotland2.pdf. Accessed January 11, 2019.

83. Purdon G, Comrie F, Rutherford L, Marcinkiewicz A. *Vitamin D Status of Scottish Adults: Results from the 2010 & 2011 Scottish Health Surveys*.; 2013. https://www.foodstandards.gov.scot/downloads/Report_Final.pdf. Accessed January 7, 2019.

84. Hedström AK. Smoking and disability progression in multiple sclerosis. *Expert Rev Neurother*. 2020;20(8):739-741. doi:10.1080/14737175.2020.1743176

For peer review only

ACKNOWLEDGEMENTS

This study is indebted to the FutureMS participants. We would like to thank non-author contributors of the FutureMS Consortium and clinical collaborators within neurology departments across NHS Scotland. With thanks to FutureMS, hosted by Precision Medicine Scotland Innovation Centre (PMSIC) and funded by a grant from the Scottish Funding Council to Precision Medicine Scotland Innovation Centre (PMS IC) and Biogen Idec Ltd Insurance. PK is supported by ECAT/Wellcome fellowship. We are grateful to Olivia Fleming for critical review and proofing of the draft manuscript.

FIGURE LEGENDS AND TABLES

Figure 1: Map of FutureMS participants by approximate location of residence at the time of diagnosis. Participant locations are not precise, located at the population centroid of the nearest SIMD intermediate zone (mean population ~4000). FutureMS cases (purple) are displayed alongside intermediate zone of residence of a random selection of 440 individuals from the SMSR (green). All map positions have latitudinal and longitudinal random noise added to prevent personal identifiability.

Figure 2 – FutureMS cohort design. BP – blood pressure; BMI – Body mass index; EDSS – Extended disability status scale; T25W – Timed 25foot walk test; PASAT – paced auditory serial addition test 3; 9HPT – nine hole peg test; SDMT – symbol digit modality test; PBMC – Peripheral blood mononuclear cells; FBC – full blood count; U&E – urea and electrolytes and renal function tests; LFT – liver function test panel; CRP – C-Reactive protein; HbA1C – glycosylated haemoglobin A1C; vit D – 25-OH-vitamin D; MRI – magnetic resonance imaging; OCT – optical coherence tomography.

Figure 3: Density plots stratifying the cohort at baseline visit. A – Distribution of EDSS (a measure of physical disability) by smoking status. B – Evidence of greater burden of depression as detected by PHQ9 in those who are unemployed at baseline.

Figure 4: Physical measures of disability across the cohort at baseline and month 12. 9-HPT is the mean between hands of the mean of two attempts at the 9-hole peg test with each hand and is a measure of upper limb disability measured in seconds (longer time reflects less dexterity). EDSS is an ordinal scale where higher scores reflect greater disability. MSFC is a continuous scale (z-score) where lower values reflect greater disability, participants who are unable to walk are arbitrarily attributed very low Z-scores for the walking component of their test (-13.7) as per published instructions. This gives a long negative tail to the distribution as the -13.7 is chosen to allow for the cohort to progress in disability with time and still capture variance in walking ability.

Figure 5: Individual level change in physical disability between the waves. A - Outlier participants who have worsened or improved over the course of wave 1 are compared in B&C for their fatigue severity score (FSS) at baseline and age at diagnosis. Circle size reflects size of difference between MSFC measurements between study visits (squared residual from least squares regression line of MSFC at year one on MSFC at year two). Outlier groups defined as above the 90th and below the 10th centile for regression residual.

Figure 6 – Fatigue Severity Score (FSS), PHQ-9 screening tool for depression, Symbol Digit Modality Tool (SDMT), Paced Serial Addition Tool (PASAT-3). Higher scores on Fatigue Severity Scale indicate worse fatigue. Higher scores on PHQ-9 indicate risk of depression. Higher scores on PASAT and SDMT indicate better performance on cognition testing and less impairment.

Figure 7 – Correlation between adjusted measures of MS disease severity (MSSS and ARMSS). Size and colour of the points reflects the patient determined diseases steps a patient reported outcome. In these figures, points are study individuals, and the size and colour of the points are scaled using the PDDS (range 0 – 7, where 7 is most severe).

*Figure 8: Clinical and radiological measures at baseline visit stratified by HLA-DRB1*15:01 genotype.*

For peer review only

	FutureMS	Scottish Multiple Sclerosis Register	p-value
**Female (n, %)**	332 (75.4%)	1916 (71.3%)	
**Male (n, %)**	108 (24.6%)	772 (28.7%)	.07
**SIMD Quintile**			
**1 (most deprived)**	71 (16.1%)	671 (18.2%)	
**2**	75 (17.0%)	718 (19.5%)	
**3**	84 (19.1%)	719 (19.5%)	
**4**	102 (23.2%)	802 (21.8%)	
**5 (least deprived)**	108 (24.5%)	770 (20.9%)	.29
**Age at symptom onset (mean (range))**	33.8 (50.69)	Data not available	
**Age at diagnosis (mean (range))**	37.7 (48.3)	38.1 (64.8)	.49

Table 1. Comparison of the baseline demographics. Data for persons with RRMS recorded between Jan 1st 2010 and Dec 31st 2017 Scottish MS Register[17]. P-values for test hypothesis that there is no difference in proportions or means, between the two study populations, calculated by Chi-squared test (proportions) or two-sided t-test (age).

For peer review only

Variable	Baseline (n = 440)	Month 12 (n = 392)
**BMI (mean, SD)**	27.9 (6.9)	28.0 (6.8)
**In employment (%)**	82.9%	82.9%
**Taking Vitamin Supplements (n, (%))**	353 (81.3%)	340 (86.7%)
**Smoking status**		
**Current (n, (%))**	64 (14.7%)	51 (13.0%)
**Ever (n, (%))**	220 (50.7%)	196 (50.1%)
**Never (n, (%))**	214 (49.3%)	195 (49.9%)
**Unknown**	6	1
**PASAT-3 (mean, (SD))**	42.4 (14.5)	44.7 (14.2)
**SDMT (mean, (SD))**	59.0 (11.5)	60.6 (12.8)
**9-hole peg test, seconds (mean, max)**	21.2 (64.8)	20.5 (121.1)
**Timed 25-foot walk, seconds (mean, max)**	5.63 (19.1)	5.55 (40.0)
**EDSS (median, (IQR))**	2.0 (1.5)	2.5 (1)
**MSFC (mean, (SD))**	-0.04 (0.86)	0.13 (0.94)
**PHQ-9 (median, IQR)**	7 (9)	4 (7)
**GAD-7 (median, IQR)**	4 (6)	4 (6)
**FSS (median, IQR)**	35 (23)	35 (30)
**MSIS-29 (median, IQR)**	47 (28)	44 (27)
**MSSS (median, range)**	5.58 (0.21-9.97)	5.58 (0.19-9.97)
**ARMSS (median, range)**	4.53 (0.49 - 9.79)	4.93 (0.43 - 9.58)
**WBV/ICV (%, (SD)) †**	78.35 (6.03)	73.76 (3.77)
**T2 WMH-V (median (range))* †**	0.69% (0.04 – 8.5)	0.82% (0.07 – 7.2)
**T2 WMH-N (yes/no) †**	N/A	192/190
**ΔWBV (Mean) †***	N/A	-0.4454%

Table 2 – Summary of baseline and month 12 clinical, radiological, and lifestyle measures. T2 WMH-V = T2 weighted white matter hyperintensity volume as a percentage of intracranial volume. T2 WMH-N = New lesions on T2 weighted MR brain (new since last study scan). WBV = Whole brain volume. ICV = Intracranial volume. ΔWBV/ICV = within individual change in WBV normalised to ICV across the study waves, a measure of brain volume loss.

**Values normalised for head size by dividing by ICV.*

†N = 328 for these analyses, the number of participants who have scans at baseline and follow up with both scans passing quality control.

SNP	Gene	Chr	Risk Allele		AA n (%)	AG n (%)	GG n (%)	RAF	Adjusted OR (95% CI)*	p-value
Rs3135388	HLA-DRB1*15:01	6	**A** (A/G)	Cases (n = 428)	42 (9.8)	204 (47.7)	182 (42.5)	0.34	3.90 (2.50 – 6.34)	8.8x10^{-9}
				Controls (n = 100)	3	18	79	0.12		

*Additive model, adjusted for sex.

*Table 3: Frequency of HLA-DRB1*15:01 in FutureMS Cases and Controls. Calculated odds ratio (OR) assumes an additive logistic regression model. RAF = Risk Allele Frequency. A = deoxyadenosine and G = deoxyguanosine at the risk loci. A is the risk allele. AA = homozygous for risk allele. Chr = Chromosome. HLA = Human leukocyte antigen. SNP = Single nucleotide polymorphism.*

For peer review only

Disease modifying therapy	Total (2nd line)	% (n = 416)
**Any DMT**	273	65.6
**>1 DMT**	23	5.5
**Dimethyl fumarate**	137 (6)	32.9*
**Interferon beta**	33 (5)	7.9*
**Glatiramer acetate**	50 (3)	12.0*
**Teriflunomide**	11 (1)	2.6*
**Cladribine**	14 (2)	3.4*
**Fingolimod**	8 (3)	1.9*
**Natalizumab**	15 (2)	3.6*
**Alemtuzumab**	28 (1)	6.7*

*Table 4 – Frequency of disease modifying therapy use at month 12 for 416 participants who have data at month 12 on DMT usage. *Percentage of cohort who used this DMT first line. DMT = Disease modifying therapy.*

For peer review only

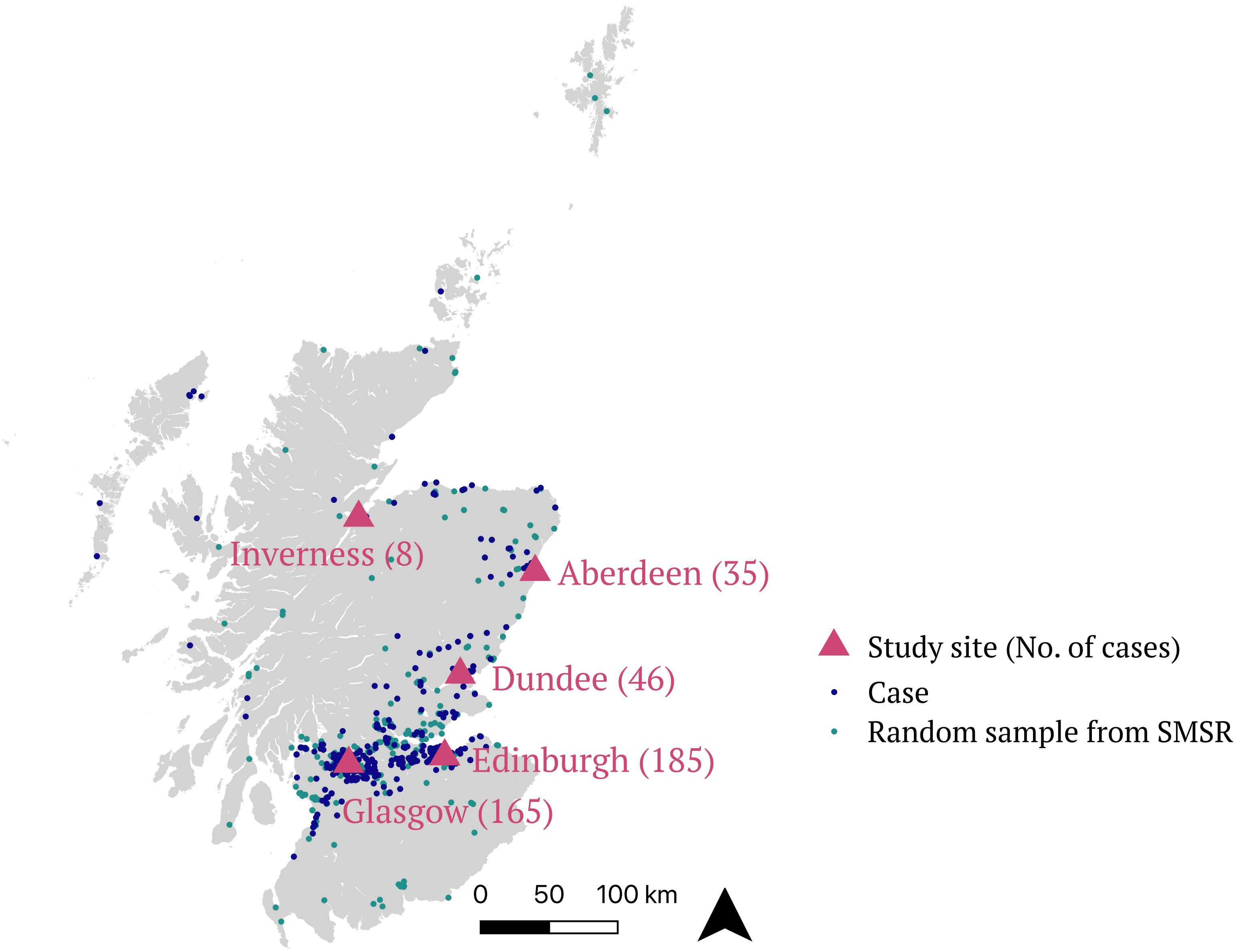
Inverness (8)
Aberdeen (35)
Dundee (46)
Edinburgh (185)
Glasgow (165)
0 50 100 km
Study site (No. of cases)
Case
Random sample from SMSR

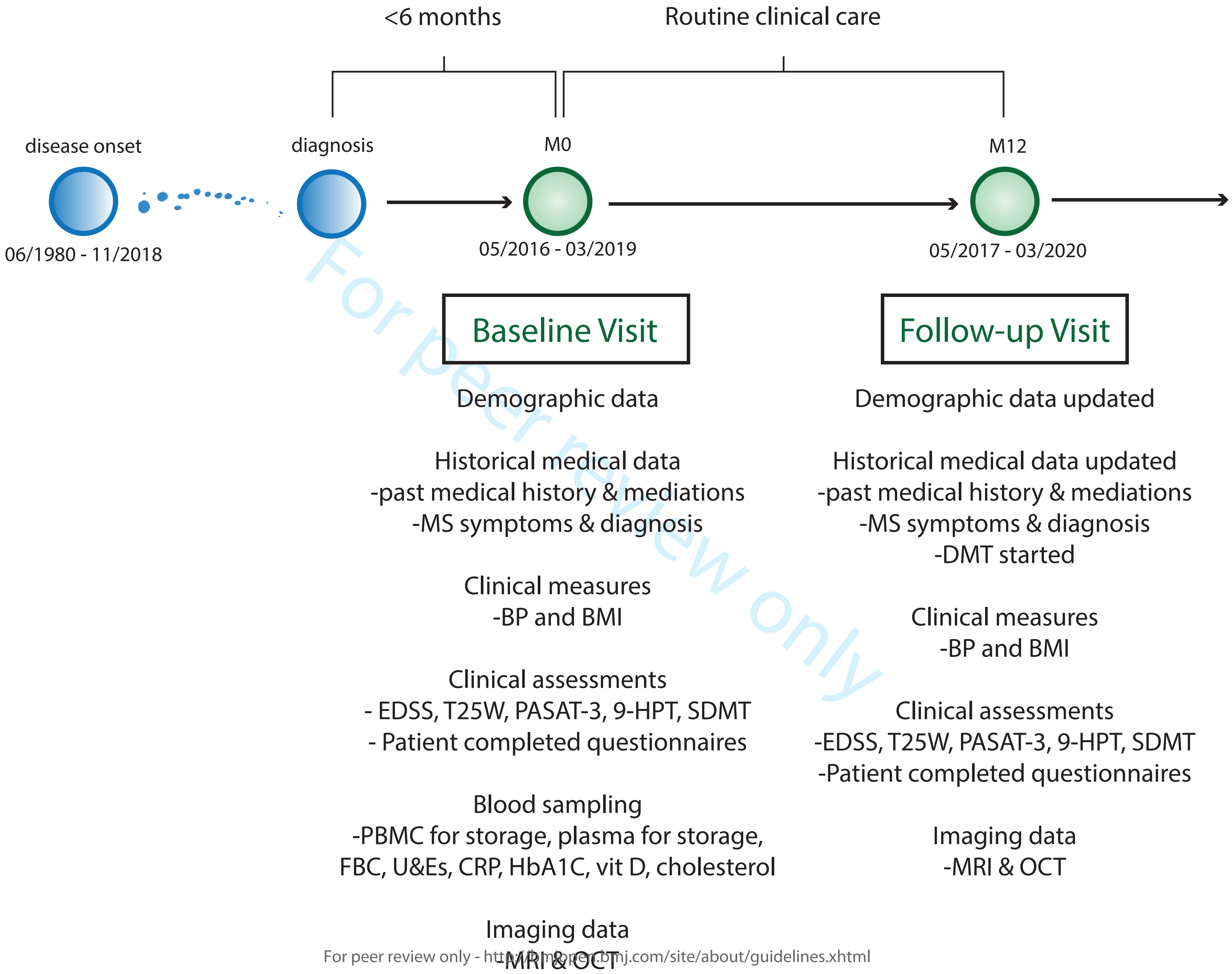

For peer review only

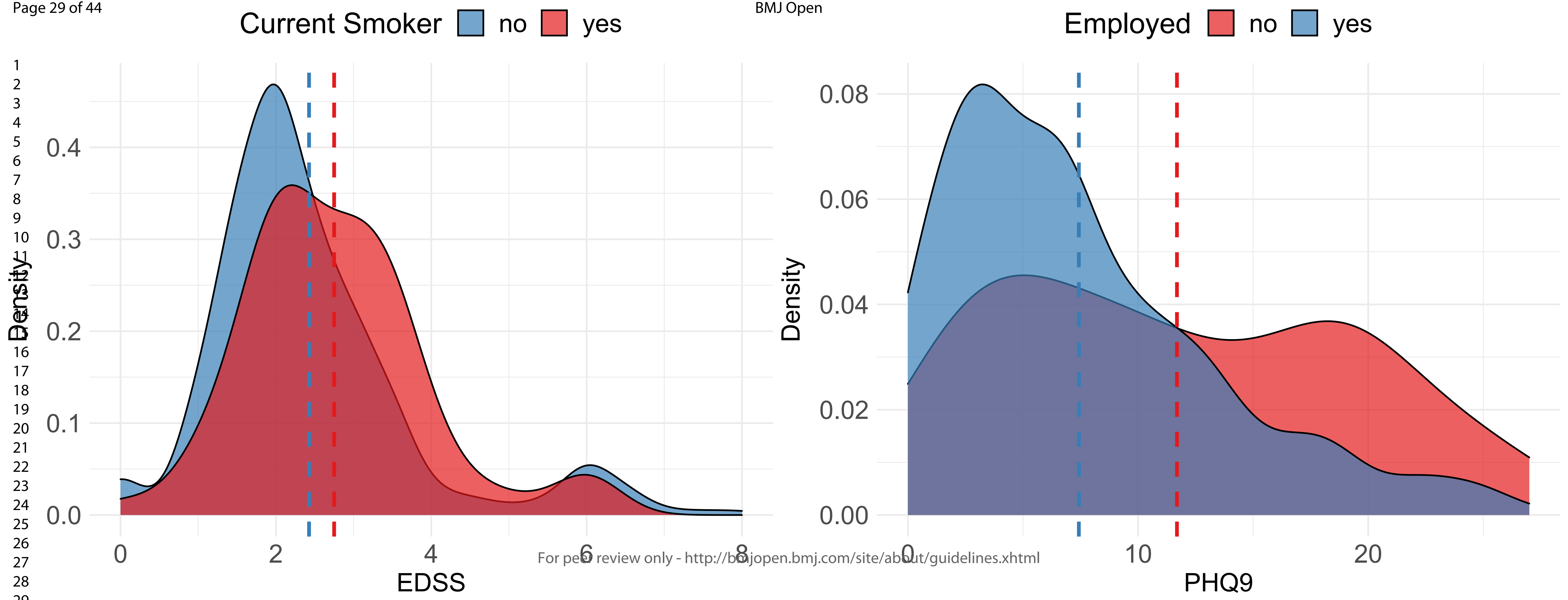
Current Smoker
no
yes
Density
0.0
0.1
0.2
0.3
0.4
0
2
4
6
8
EDSS
Employed
no
yes
Density
0.00
0.02
0.04
0.06
0.08
0
10
20
PHQ9

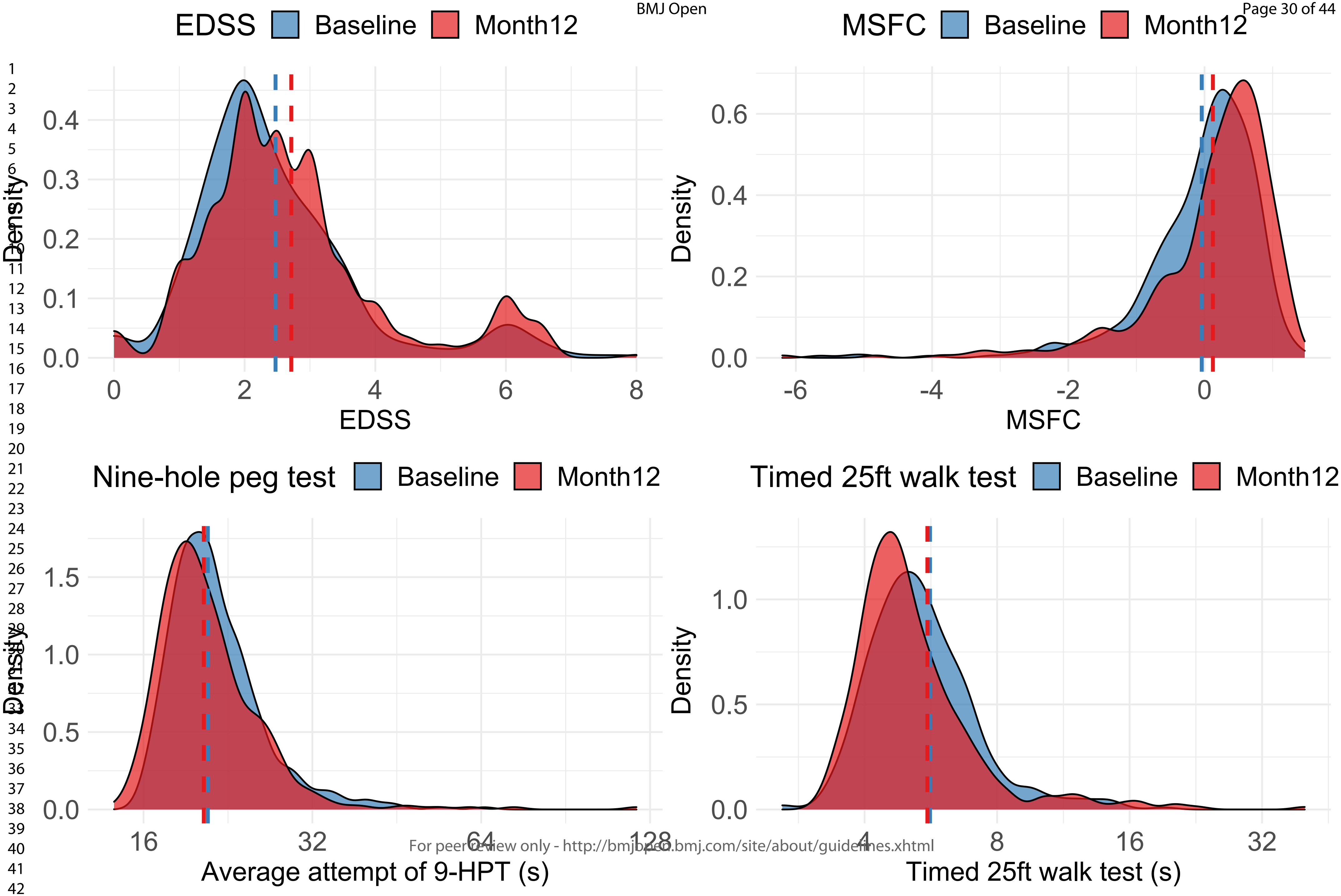
EDSS Baseline Month12
Density
0.0
0.1
0.2
0.3
0.4
0
2
4
6
8
EDSS
MSFC Baseline Month12
Density
0.0
0.2
0.4
0.6
-6
-4
-2
0
MSFC
Nine-hole peg test Baseline Month12
Density
0.0
0.5
1.0
1.5
16
32
64
128
Average attempt of 9-HPT (s)
Timed 25ft walk test Baseline Month12
Density
0.0
0.5
1.0
4
8
16
32
Timed 25ft walk test (s)

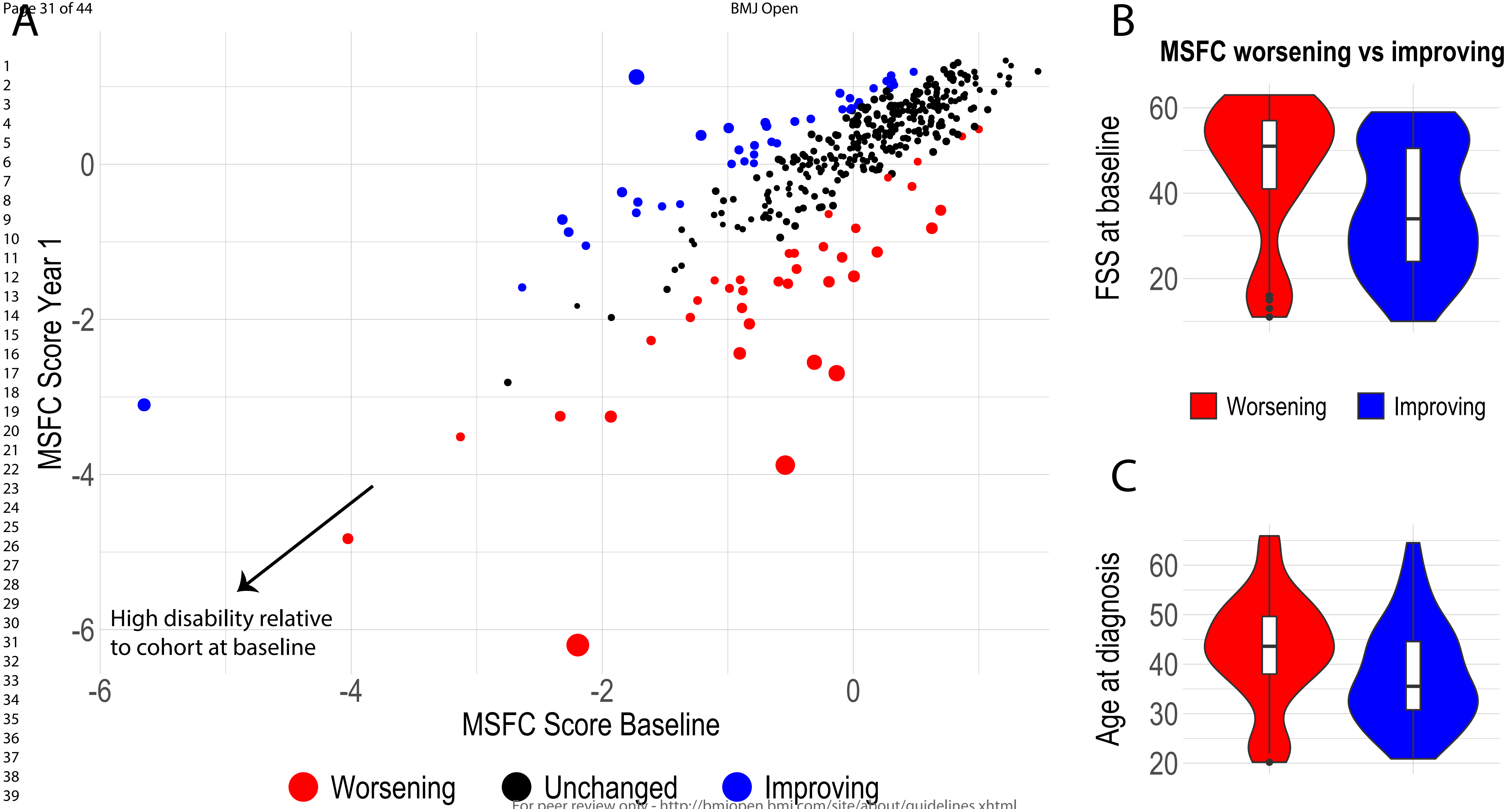
A
MSFC Score Year 1
0
-2
-4
-6
-6
-4
-2
0
MSFC Score Baseline
High disability relative
to cohort at baseline
Worsening
Unchanged
Improving
B
MSFC worsening vs improving
FSS at baseline
60
40
20
Worsening
Improving
C
Age at diagnosis
60
50
40
30
20

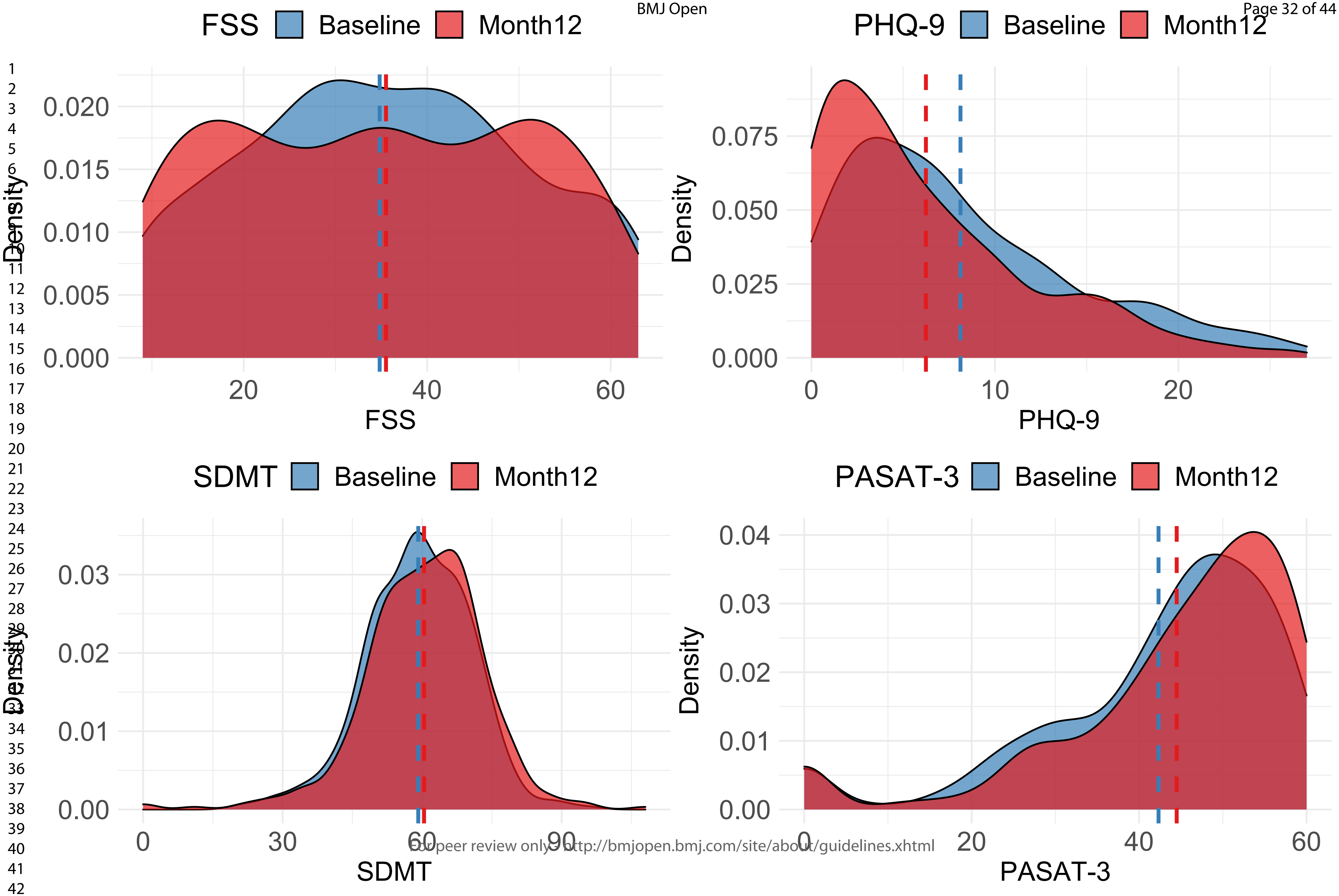

For peer review only - http://bmjopen.bmj.com/site/about/guidelines.xhtml

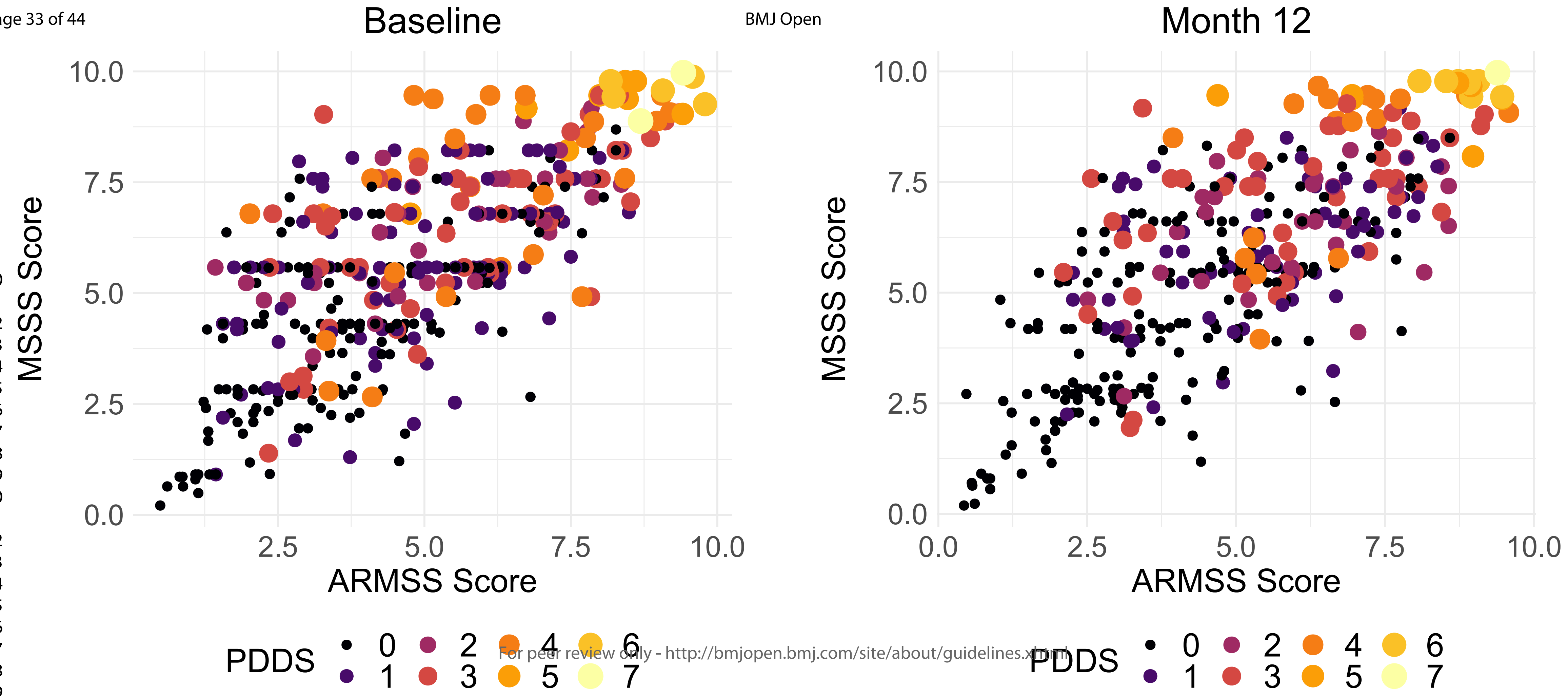

For peer review only - http://bmjopen.bmj.com/site/about/guidelines.xhtml

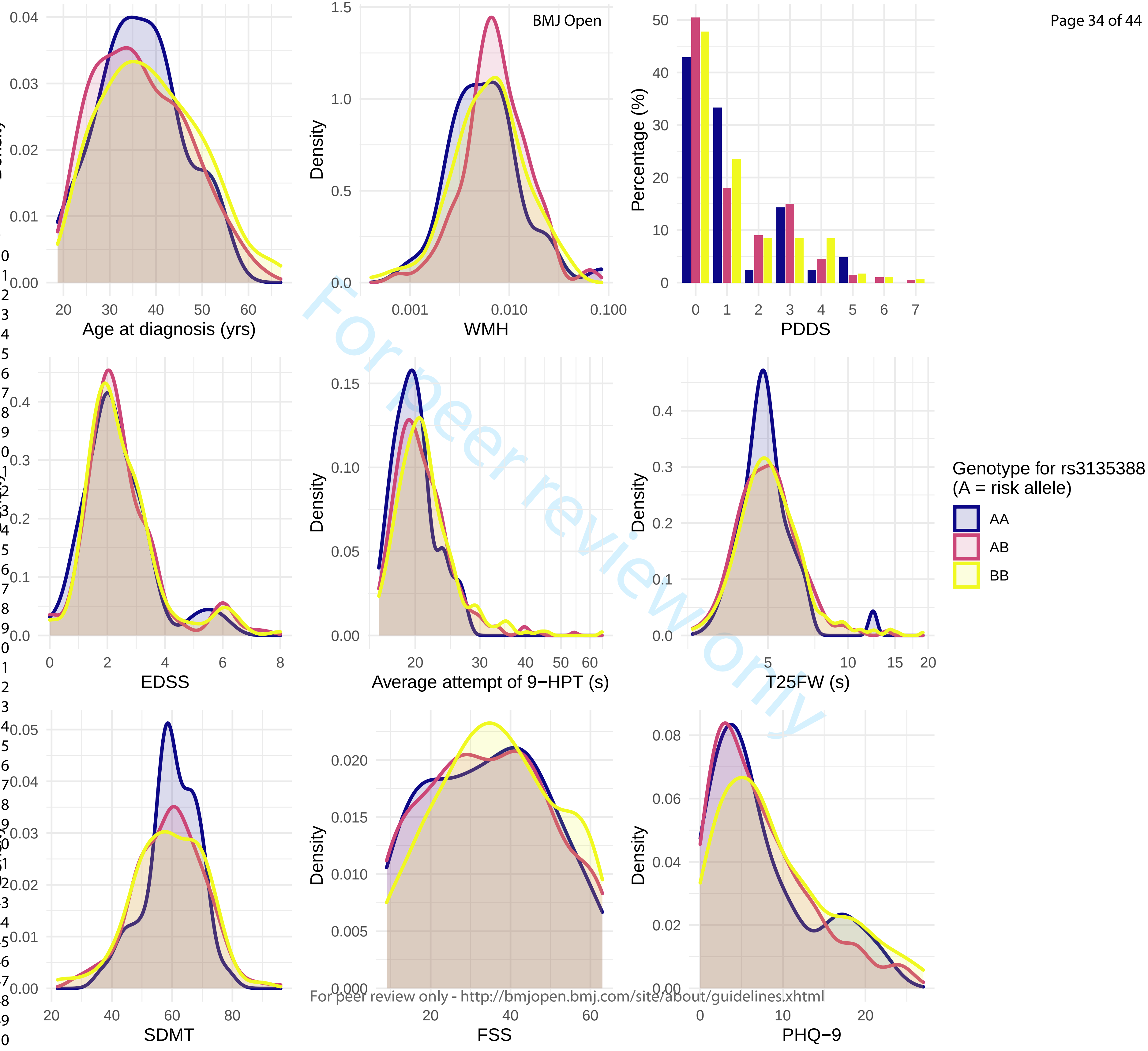

For peer review only

SUPPLEMENTARY FIGURES AND TABLES

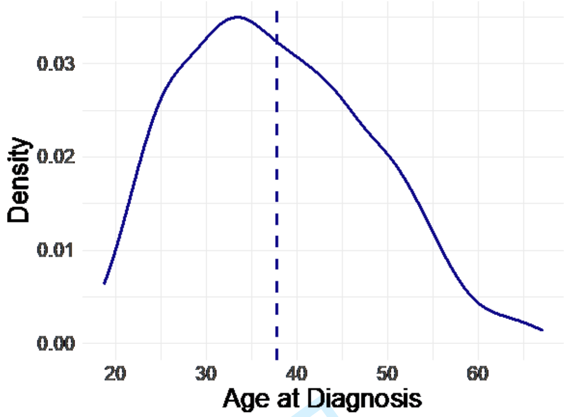

Figure S1. Age at diagnosis profile of the FutureMS participants. Demonstrating monomodal distribution and truncation at extremes of age (particularly <18) due to inclusion criteria. Dotted line is the mean age at diagnosis.

For peer review only

ASSESSMENT	Baseline	Year 1	Year 5
Informed Consent	X		X
Demographics	X		
Medical History	X	X	X
Social History	X	X	X
Family History	X	X	X
Migration History			X
Relapse/Progression History	X	X	X
Medication History	X	X	X
Infectious Mononucleosis History			X
Structured Neurological Examination	X	X	X
BMI, Height, Weight, BP	X	X	X
Measures of Physical Disability (EDSS, T25FTWT, 9HPT)	X	X	X
Cognitive tests (PASAT-3, SDMT)	X	X	X
PHQ-9 and GAD-7	X	X	X
MSIS-29	X	X	X
Fatigue Severity Score	X	X	X
Patient Derived Disease Severity Score	X	X	X
Visual Acuity	X	X	X
Standard MRI Protocol	X	X	X
Disease Modifying Therapy History	N/A	X	X
Lymphocyte subsets stored (CD3+CD4+, CD3+CD8+, CD19+, CD14+)	X		
Serum stored	X		X
Saliva stored			X
DNA Genotyping	X		
Advanced MRI Imaging (SS3)	X	X	X
Optical Coherence Tomography (SS4)	X	X	X

Table S1. Summary of measures at each study visit. SS3 = Substudy 3. SS4 = Substudy 4.

For peer review only

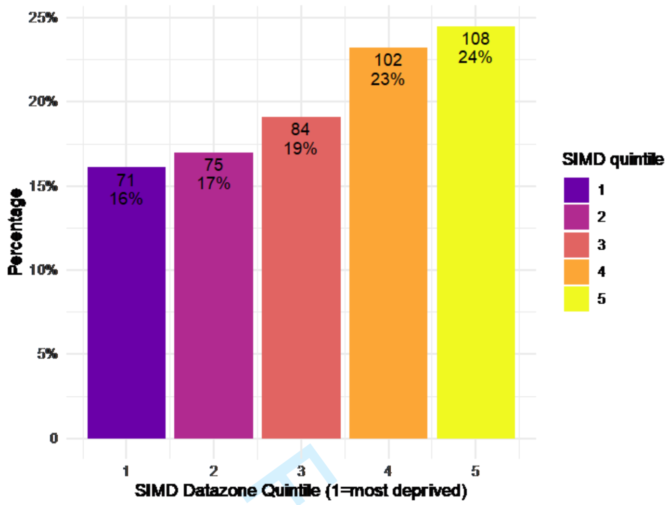

Figure S2. Scottish Index of Multiple Deprivation (SIMD) quintile of study participants.

	Parent/Grandparent	% of Parent				% of Grandparent			
		Mean	SD	LQ10	UQ10	Mean	SD	LQ10	UQ10
Single cells	Events	56.05	15.98	35.60	75.00				
Viable (7AAD -ve)	Single cells	91.77	10.11	97.80	84.93.				
CD14+ Monocyes	CD3-/viable cells	22.03	11.67	34.30	10.40				
CD19+ B cells	CD3-/viable cells	8.11	3.69	4.08	12.9				
CD4+ T cells	CD3+/viable cells	69.44	11.67	81.47	57.00	38.97	10.48	51.27	26.56
CD8+ T cells	CD3+/viable cells	20.27	8.31	30.37	11.22	20.27	8.31	30.37	11.22

Table S2. Proportion of PBMCs expressing markers for monocytes (CD14+), B/plasma cells (CD19+), helper (CD3+CD4+) and cytotoxic (CD3+CD8+) t cells in the peripheral blood mononuclear cells donated by patients to the study.

For peer review only

	With PBMCs	FACs sorted	Total
Edinburgh (01)	184	184	185
Glasgow (02)	163	137	165
Dundee (03)	46	46	46
Aberdeen (04)	35	32	35
Inverness (05)	8	6	8
Controls	100	78	103
**Totals (patients only)**	**436**	**405**	**439**
**Totals**	**536**	**483**	**542**

Table S3: Summary of numbers of FutureMS study subjects and controls with peripheral blood mononuclear cells available for analysis.

For peer review only

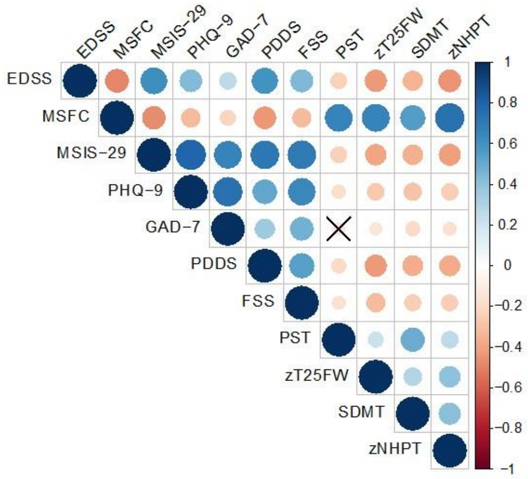

Figure S3. Correllelogram of clinical measures at baseline visit. zT25FW is the z-score of the timed 25-foot walk test. zNHPT is the z-score of the 9-hole peg test. Other acronyms as defined in the main text.

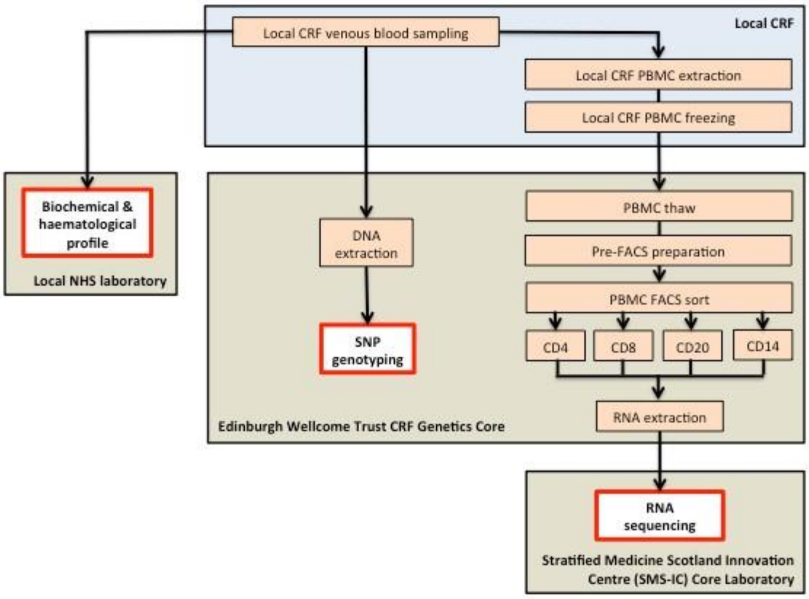

Figure S4: Schematic explaining laboratory pathway for tissue collection for biomarker processing.

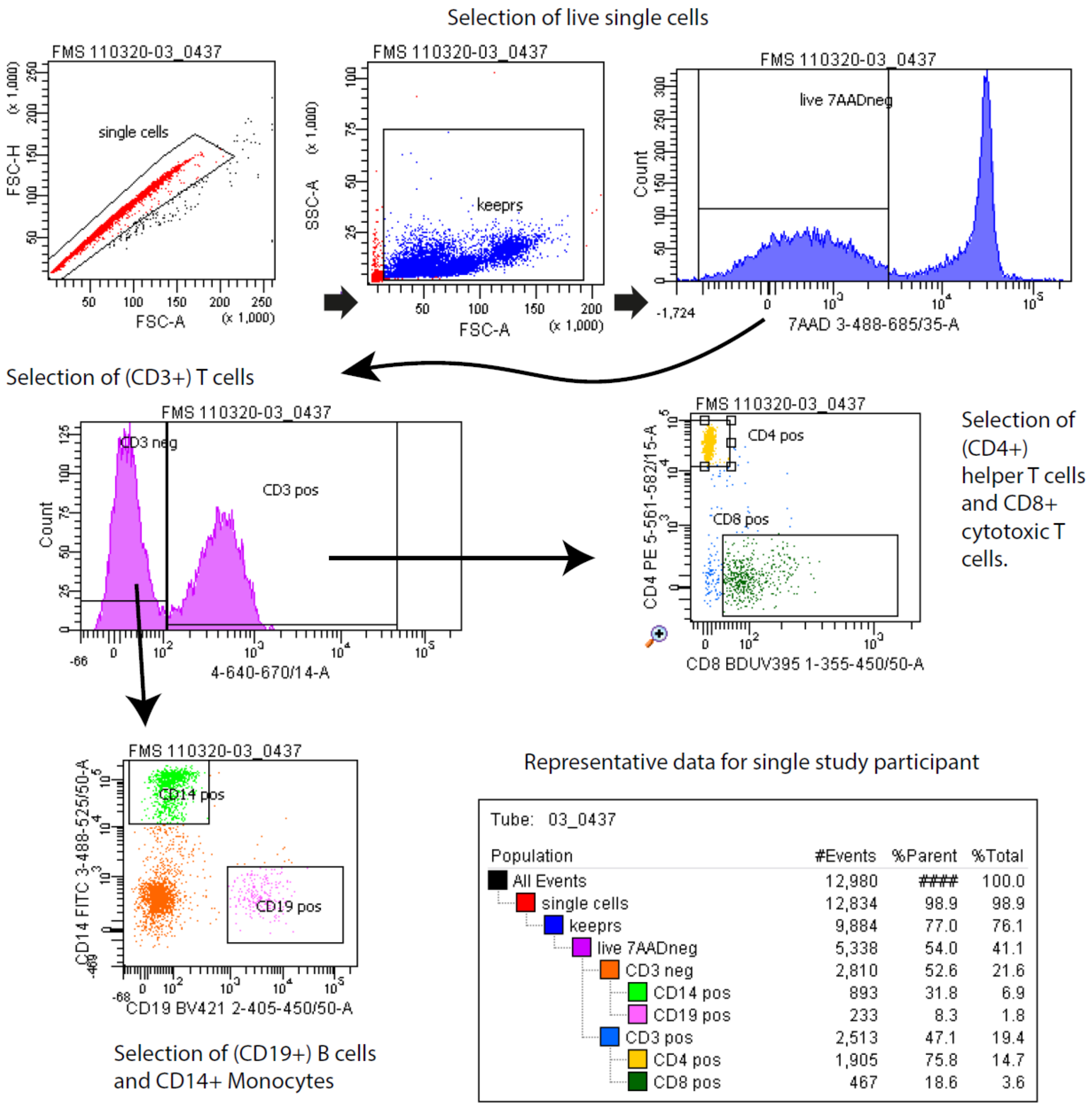

Population	#Events	%Parent	%Total
All Events	12,980	####	100.0
single cells	12,834	98.9	98.9
keeprs	9,884	77.0	76.1
live 7AADneg	5,338	54.0	41.1
CD3 neg	2,810	52.6	21.6
CD14 pos	893	31.8	6.9
CD19 pos	233	8.3	1.8
CD3 pos	2,513	47.1	19.4
CD4 pos	1,905	75.8	14.7
CD8 pos	467	18.6	3.6

Figure S5. Example of the fluorescence-activated cell sorting undertaken for a representative member of the FMS cohort. CD3 – T cell marker. CD4+ helper T cells, CD8+ cytotoxic T cells. CD19+ B cell marker and CD14+ monocyte marker. 7AAD – 7-Aminoactinomycin D uptake associated with loss of cell viability.

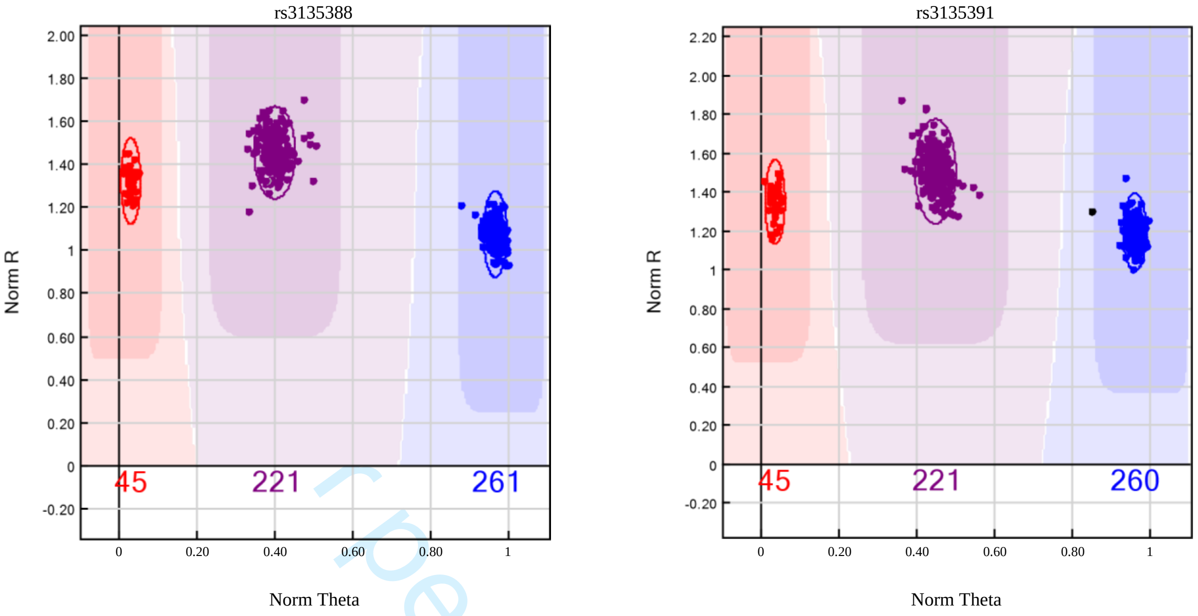

*Figure S6. Demonstration of two SNPs in linkage disequilibrium from the FutureMS cohort demonstrating clear separation of clusters resulting in high confidence of genotype calls. These two SNPs mark HLA-DRB1*15:01, which contributes the largest single gene effect for multiple sclerosis.*

For peer review only

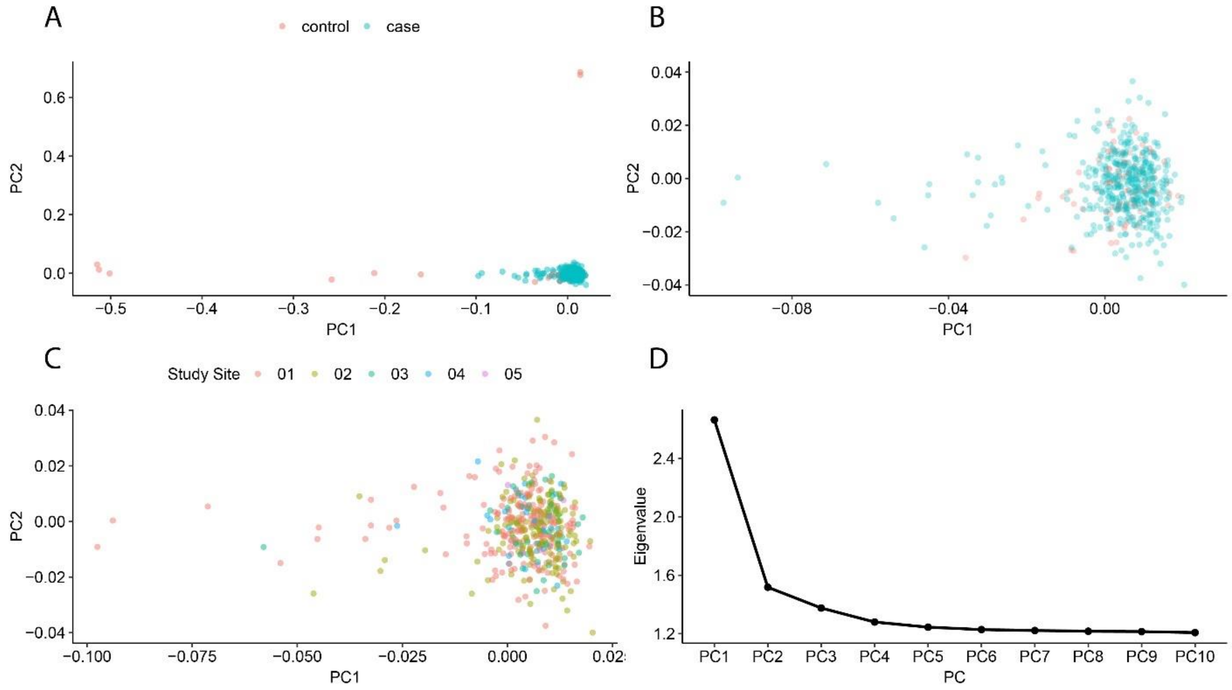

Figure S7. Genetic stratification analysis of the FutureMS cohort and controls. A – plot of first two principal components, comparing cases and controls. B – enlarged region of A with most cases and controls tightly clustered. Note difference of scale in PCs in A&B and A&C. C – Cases demonstrate little evidence of genetic stratification within the cohort based on the study site they were recruited from (scaled as in B). D – Scree plot of Eigenvalues of first ten principal components.

STROBE Statement—Checklist of items that should be included in reports of ***cohort studies***

	Item No	Recommendation	Page No
**Title and abstract**	1	(*a*) Indicate the study's design with a commonly used term in the title or the abstract	1
		(*b*) Provide in the abstract an informative and balanced summary of what was done and what was found	1
**Introduction**			
Background/rationale	2	Explain the scientific background and rationale for the investigation being reported	2
Objectives	3	State specific objectives, including any prespecified hypotheses	2
**Methods**			
Study design	4	Present key elements of study design early in the paper	3
Setting	5	Describe the setting, locations, and relevant dates, including periods of recruitment, exposure, follow-up, and data collection	3
Participants	6	(*a*) Give the eligibility criteria, and the sources and methods of selection of participants. Describe methods of follow-up	3
		(*b*) For matched studies, give matching criteria and number of exposed and unexposed	N/A
Variables	7	Clearly define all outcomes, exposures, predictors, potential confounders, and effect modifiers. Give diagnostic criteria, if applicable	3
Data sources/ measurement	8*	For each variable of interest, give sources of data and details of methods of assessment (measurement). Describe comparability of assessment methods if there is more than one group	3
Bias	9	Describe any efforts to address potential sources of bias	5
Study size	10	Explain how the study size was arrived at	3
Quantitative variables	11	Explain how quantitative variables were handled in the analyses. If applicable, describe which groupings were chosen and why	5
Statistical methods	12	(*a*) Describe all statistical methods, including those used to control for confounding	6
		(*b*) Describe any methods used to examine subgroups and interactions	6
		(*c*) Explain how missing data were addressed	6
		(*d*) If applicable, explain how loss to follow-up was addressed	6
		(*e*) Describe any sensitivity analyses	6
**Results**			
Participants	13*	(a) Report numbers of individuals at each stage of study—eg numbers potentially eligible, examined for eligibility, confirmed eligible, included in the study, completing follow-up, and analysed	4
		(b) Give reasons for non-participation at each stage	4
		(c) Consider use of a flow diagram	Fig2
Descriptive data	14*	(a) Give characteristics of study participants (eg demographic, clinical, social) and information on exposures and potential confounders	Tab1
		(b) Indicate number of participants with missing data for each variable of interest	5
		(c) Summarise follow-up time (eg, average and total amount)	Fig2
Outcome data	15*	Report numbers of outcome events or summary measures over time	Results

For peer review only - http://bmjopen.bmj.com/site/about/guidelines.xhtml

Main results	16	(*a*) Give unadjusted estimates and, if applicable, confounder-adjusted estimates and their precision (eg, 95% confidence interval). Make clear which confounders were adjusted for and why they were included	N/A
		(*b*) Report category boundaries when continuous variables were categorized	N/A
		(*c*) If relevant, consider translating estimates of relative risk into absolute risk for a meaningful time period	N/A
Other analyses	17	Report other analyses done—eg analyses of subgroups and interactions, and sensitivity analyses	N/A
**Discussion**			
Key results	18	Summarise key results with reference to study objectives	Discussion
Limitations	19	Discuss limitations of the study, taking into account sources of potential bias or imprecision. Discuss both direction and magnitude of any potential bias	Discussion
Interpretation	20	Give a cautious overall interpretation of results considering objectives, limitations, multiplicity of analyses, results from similar studies, and other relevant evidence	Discussion
Generalisability	21	Discuss the generalisability (external validity) of the study results	Discussion
**Other information**			
Funding	22	Give the source of funding and the role of the funders for the present study and, if applicable, for the original study on which the present article is based	Funding statement

*Give information separately for exposed and unexposed groups.

Note: An Explanation and Elaboration article discusses each checklist item and gives methodological background and published examples of transparent reporting. The STROBE checklist is best used in conjunction with this article (freely available on the Web sites of PLoS Medicine at http://www.plosmedicine.org/, Annals of Internal Medicine at http://www.annals.org/, and Epidemiology at http://www.epidem.com/). Information on the STROBE Initiative is available at http://www.strobe-statement.org.

For peer review only
